# Safety and efficacy of 24 weeks of pemvidutide in metabolic dysfunction-associated steatotic liver disease: A randomized, controlled clinical trial

**DOI:** 10.1016/j.jhepr.2025.101483

**Published:** 2025-06-18

**Authors:** Sarah K. Browne, John J. Suschak, Shaheen Tomah, Julio A. Gutierrez, Jay Yang, Bertrand Georges, M. Scot Roberts, M. Scott Harris

**Affiliations:** 1 Altimmune, Inc, Gaithersburg, MD, USA; 2 Center for Organ Transplant, Scripps Clinic, La Jolla, CA, USA

**Keywords:** Metabolic dysfunction-associated steatotic liver disease, Metabolic dysfunction-associated steatohepatitis, ALT-801, Pemvidutide, GLP-1, Glucagon, Efruxifermin, Resmetirom

## Abstract

**Background & Aims:**

This was a double-blind 12-week extension of a randomized, placebo-controlled, 12-week trial of pemvidutide, a glucagon-like peptide-1/glucagon dual receptor agonist, in individuals with metabolic dysfunction-associated steatotic liver disease (MASLD).

**Methods:**

Completers of a double-blind trial of pemvidutide in MASLD, who were previously randomized 1:1:1:1 to pemvidutide at 1.2 mg, 1.8 mg, or 2.4 mg, or placebo administered subcutaneously once weekly for 12 weeks, were offered an additional 12 weeks of treatment at their originally assigned dose for a total of 24 weeks of treatment. Participants were stratified by the presence or absence of type 2 diabetes mellitus (T2DM). The primary efficacy endpoint was relative reduction (%) from baseline in liver fat content by magnetic resonance imaging-proton density fat fraction after 24 weeks of treatment.

**Results:**

There were 64 participants in the extension trial. Baseline mean values for BMI and liver fat content were 36.7 kg/m^2^ and 22.2%; 26.6% of participants had T2DM. After 24 weeks of treatment, pemvidutide achieved relative reductions in liver fat content from baseline of 56.3%, 75.2%, and 76.4% for the pemvidutide 1.2 mg, 1.8 mg, and 2.4 mg groups respectively, *vs.* 14.0% for placebo (*p* <0.001 *vs.* placebo, all treatment groups), with 84.6% of participants achieving 50% reductions in liver fat content and 53.8% achieving normalization (≤5% liver fat content) at the 1.8 mg dose. Body weight was also reduced by 6.2% (*p* <0.001 *vs.* placebo) over 24 weeks of treatment. Pemvidutide was well-tolerated at all doses, with low incidences of side effects.

**Conclusions:**

In individuals with MASLD, 24 weeks of pemvidutide treatment resulted in significant reductions in liver fat content and body weight that further improved upon the effects observed at 12 weeks.

**Impact and implications:**

Overweight and obesity are strongly associated with metabolic dysfunction-associated steatotic liver disease and metabolic dysfunction-associated steatohepatitis (MASH), as the excess liver fat associated with obesity is a known driver of these conditions. Glucagon-like peptide-1 receptor (GLP-1R) agonists elicit weight loss through centrally and peripherally mediated effects on appetite, whereas G-coupled glucagon receptor (GCGR) agonists act directly on the liver to stimulate fatty acid oxidation and inhibit lipogenesis, providing a more potent mechanism for reducing liver fat content than weight loss alone. We previously showed that once-weekly treatment with pemvidutide, a dual GLP-1R/GCGR agonist, significantly reduced liver fat content, hepatic inflammatory activity, and body weight over 12 weeks. The current trial demonstrates that continued treatment with pemvidutide further improves these clinical markers of MASH.

**Clinical Trials Registration:**

The study is registered at ClinicalTrials.gov (NCT05292911).

## Highlights

• Pemvidutide is a GLP-1/glucagon dual receptor agonist.
• Significant reductions in liver fat content following 24 weeks of treatment.
• Significant reductions in non-invasive biomarkers of liver inflammation.
• Pemvidutide treatment significantly reduced body weight.
• Pemvidutide may be an effective treatment for MASH.

## Graphical abstract

**Figure:**
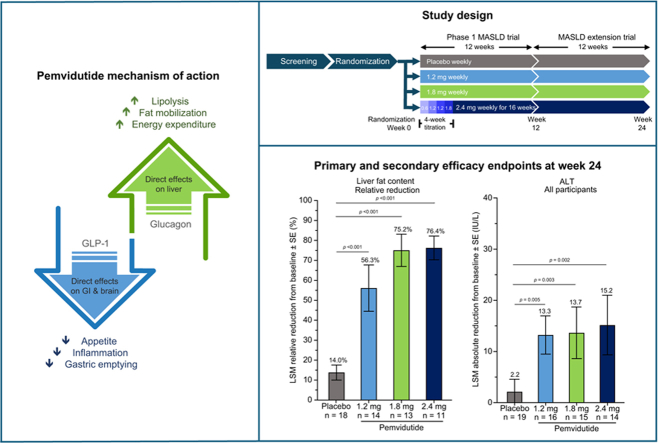

## Introduction

Metabolic dysfunction-associated steatotic liver disease (MASLD) is characterized by hepatic steatosis >5% in individuals with no or minimal alcohol use and is associated with cardiometabolic risk factors such as obesity, dyslipidemia, insulin resistance, and type 2 diabetes.^1^ Between 20% and 30% of patients with MASLD progress to metabolic dysfunction-associated steatohepatitis (MASH), which is characterized by inflammation, and hepatocyte ballooning, with or without fibrosis.^2^^,^^3^ Approximately 20% of patients with MASH will progress to end-stage liver disease and may require liver transplantation. Until the year 2024, there were no approved treatments for MASH. Although the approved therapy, a thyroid hormone receptor-β agonist, demonstrated efficacy against MASH and improvements in hepatic fibrosis, only a small, but statistically significant, proportion of the participants realized a reduction in hepatic fibrosis and treatment did not reduce body weight, a primary driver of MASH and the cardiometabolic complications of obesity.^4^ These data suggest that agents that can improve the treatment response rate and address the underlying obesity and obesity-related comorbidities are needed as these are the primary causes of morbidity and mortality in patients with MASH.^5^

Glucagon-like peptide-1 receptor (GLP-1R) agonist monotherapy has demonstrated resolution of MASH, but it has not shown statistically significant improvements in fibrosis, possibly because reductions in liver fat content (LFC), a key driver of MASH, are indirect and the result of body weight loss.[6], [7], [8] Conversely, the direct effects of glucagon on the liver are well known and include the stimulation of hepatic β-oxidation of fatty acids and a reduction of *de novo* lipogenesis.^9^^,^^10^ It was hypothesized that combining GLP-1R and G-coupled glucagon receptor (GCGR) agonism in the same molecule could achieve levels of liver fat reduction greater than those achievable by GLP-1R activation alone. Dual GLP-1R and GCGR agonism may also yield greater weight loss than GLP-1R monotherapy alone as glucagon-like peptide-1 (GLP-1) reduces food consumption through anorectic effects while glucagon stimulates energy expenditure, potentially mimicking the effects of diet and exercise.[11], [12], [13], [14]

We recently reported on the design of pemvidutide, a unimolecular, peptide-based GLP-1R/GCGR dual receptor agonist with balanced potency against the GLP-1 and GCG receptors.^15^ In a preclinical mouse model of MASH, pemvidutide demonstrated robust weight loss and significant reductions in liver fat, liver inflammation, and liver fibrosis compared with the GLP-1R agonist semaglutide.^16^ In a 12-week randomized, double-blind, placebo-controlled clinical trial in individuals with MASLD, pemvidutide-treated participants exhibited statistically significant reductions in LFC, markers of hepatic inflammation including alanine aminotransferase (ALT), iron-corrected T1 (cT1) magnetic resonance imaging (MRI), and body weight.^17^

Here, we present the results of a 12-week double-blind extension trial (NCT05292911) in participants who completed the parent 12-week MASLD trial (NCT05006885). The trial evaluated the safety, tolerability, and efficacy of pemvidutide in a MASLD population and measured reduction in LFC and associated markers of inflammation and fibrosis following 24 weeks of treatment.

## Patients and methods

### Trial design and participant selection

This was a 12-week extension to a 12-week multicenter, randomized, double-blind, placebo-controlled trial to assess the safety of pemvidutide in participants with MASLD. The trial was conducted in accordance with the principles of the Declaration of Helsinki and the International Conference on Harmonization Good Clinical Practice. The trial protocol and amendments were approved by the relevant institutional review boards and ethics committees at each trial site. All participants provided written informed consent before participation in the trial.

To be eligible for this extension trial, participants were required to complete the parent 12-week phase I MASLD trial (NCT05006885) and have remained on treatment for the 12-week period. The eligibility criteria of the parent and extension trials were the same. Participants in the parent trial were required to be 18–65 years of age, have a BMI ≥28.0 kg/m^2^ and an LFC of ≥10% by MRI-proton density fat fraction (MRI-PDFF). Individuals with type 2 diabetes mellitus (T2DM) were permitted if they had a glycated hemoglobin (HbA1c) level <9.5% and were on a stable regimen of one or more of the following for at least 3 months before screening: diet and exercise, metformin with no more than mild gastrointestinal symptoms (nausea, vomiting, or diarrhea), and/or a sodium glucose transporter-2 (SGLT-2) inhibitor therapy. Individuals with significantly elevated serum ALT levels, defined as >75 IU/L, or significant hepatic fibrosis, defined as FibroScan® (Echosens, Paris, France) liver stiffness measurement of ≥10 kPa, suggestive of more advanced disease, were excluded. Use of insulin or GLP-1R-based therapies was also excluded.

### Trial procedures

Participants who enrolled in the extension trial remained on the same treatment to which they were initially randomized 1:1:1:1 by interactive web response system at the start of the 12-week parent trial (NCT05006885). Participants continued to receive either 1.2 mg pemvidutide, 1.8 mg pemvidutide, 2.4 mg pemvidutide, or saline placebo (without titration) administered weekly by subcutaneous injection for an additional 12 weeks, with blinding maintained for the investigator, participant, and trial staff. Dose reduction for intolerability was not used and participants were instructed to maintain their normal diet and activities, without diet or exercise interventions, for the duration of the 24-week combined trial duration.

### Efficacy endpoints

The primary efficacy endpoints were the absolute and relative (percent, %) reduction in LFC from baseline values in the parent trial by MRI-PDFF. Secondary efficacy endpoints included absolute change from baseline in serum ALT in the parent trial and percent change from baseline in body weight in the parent trial. Additional endpoints included liver volume by MRI-PDFF, FibroScan controlled attenuation parameter (CAP) score, FibroScan vibration-controlled transient elastography (VCTE), enhanced liver fibrosis (ELF®), pro-peptide of type III collagen (PRO-C3), and lipid metabolism (total cholesterol, LDL, HDL, and triglycerides). A subset of 22 participants participated in a cT1 imaging study to assess the effects of pemvidutide on this additional marker of hepatic inflammation. Responder analyses included the proportions of participants achieving 30% or 50% relative reductions in LFC or normalization, defined as LFC ≤5%, and the proportion of participants with a ≥80 ms reduction in cT1 relaxation time. Safety endpoints included adverse events (AEs), electrocardiograms (ECGs), vital signs (systolic blood pressure [BP], diastolic BP, heart rate), and glucose homeostasis as assessed by fasting serum glucose and HbA1c.

### Statistical analysis

The efficacy analysis population included all randomized participants who received at least one dose of study medication and completed at least one post-baseline assessment. Continuous variables limited to a single pre- and post-treatment measurement, including changes from baseline in LFC, cT1 relaxation time, fibrosis markers, inflammation markers, and lipids, were compared between pemvidutide and placebo using statistical tests based on analysis of covariance (ANCOVA), with the treatment arm as a factor and stratification for presence or absence of T2DM and the corresponding baseline demographic characteristics (sex, race, BMI) as covariates. Continuous measures with multiple measurements, such as weekly changes in body weight, serum ALT, serum aspartate aminotransaminase (AST), BP, and heart rate, were analyzed using mixed model of repeated measures (MMRM) with the same covariates as ANCOVA under the assumption of missing values at random, using a pattern mixture model under the assumption of missing not at random as a sensitivity analysis. The results of ANCOVA and MMRM analyses were expressed as least squares mean (LSM). The Cochrane–Mantel–Haenszel test or Fisher’s exact test was applied to secondary endpoints that were categorical in nature, while considering the stratification of presence or absence of T2DM, at a one-sided significance level of 0.025. Only non-missing values were included in these analyses. The numbers of participants experiencing treatment-emergent AEs were summarized by Medical Dictionary for Regulatory Activities System Organ Class, Preferred Term, seriousness, severity, and relationship to study medication. All statistical analyses refer to the respective treatment group *vs.* placebo unless otherwise stated.

The sample size of the parent 12-week trial was designed to enable a qualitative evaluation for safety imbalances between placebo and study drug recipients and supports adequate descriptive statistical analyses of pharmacodynamic parameters (*i.e.* efficacy endpoints).

## Results

### Trial participants

**Fig. 1:**
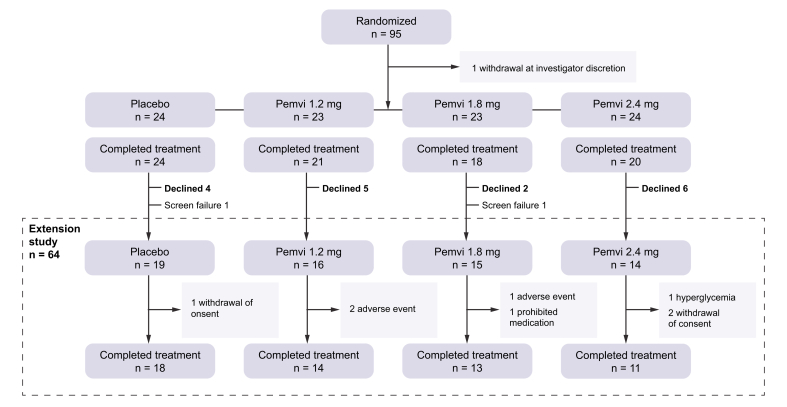
CONSORT diagram.

The extension study was conducted between March 2022 and October 2022 at 13 investigative sites in the United States. Of the 94 participants treated in the parent study, 83 completed 12 weeks of treatment and 66 consented to participate in the 12-week extension, of whom 64 met eligibility criteria to participate. The 17 individuals who declined participation in the extension trial had similar responses to treatment at 12 weeks as the 64 participants who extended treatment to 24 weeks and primarily cited scheduling conflicts (Table S1). The disposition of trial participants is shown in Fig. 1.

The baseline characteristics of the participants enrolled in the extension trial, determined at the initiation of the parent 12-week trial, are provided in Table 1. Overall, the baseline characteristics for the 64 participants in the extension trial were similar to those of the 94 participants treated in the parent trial (Table S2). The mean age of trial participants across the four treatment groups was 49.0 years. The trial was balanced between males and females. Across all trial groups, 26.6% of participants in the extension trial had T2DM at baseline and 73.4% of participants were of Hispanic ethnicity. Mean body weight was 103.1 kg, and mean BMI was 36.7 kg/m^2^. Mean baseline LFC was 22.2%, mean baseline ALT was 37.2 IU/L, and mean cT1 was 914.9 ms.

**Table 1:** Participant demographics and baseline characteristics (safety population).

Characteristic	Treatment			
	Placebo (n = 19)	Pemvidutide 1.2 mg (n = 16)	Pemvidutide 1.8 mg (n = 15)	Pemvidutide 2.4 mg (n = 14)
Age, mean years (SD)	49.0 (15)	48.6 (11)	49.9 (10)	48.4 (8)
Sex, n (%)				
Female	11 (57.9)	7 (43.8)	8 (53.3)	8 (57.1)
Ethnicity, n (%)				
Hispanic	11 (57.9)	15 (93.8)	12 (80.0)	9 (64.3)
Not Hispanic	8 (42.1)	1 (6.3)	3 (20.0)	5 (35.7)
Body weight, kg (SD)	104.4 (21.2)	101.4 (16.3)	100.9 (13.2)	107.4 (17.2)
BMI, kg/m^2^ (SD)	37.1 (4.9)	36.7 (6.1)	36.0 (3.8)	37.0 (5.3)
Liver fat content, % (SD)	24.0 (9.6)	20.1 (7.7)	23.9 (7.4)	20.5 (6.5)
ALT, IU/L (SD)	41.0 (21.3)	32.4 (14.2)	35.3 (13.0)	39.6 (26.6)
AST, IU/L (SD)	25.1 (10.5)	24.4 (6.7)	23.6 (6.9)	29.4 (15.5)
Diabetes status, n (%)				
Type 2 diabetes	5 (26.3%)	3 (18.8%)	6 (40.0%)	3 (21.4%)
Non-diabetes				
Fasting glucose, mmol/L (SD)	5.3 (0.7)	5.5 (0.7)	5.3 (0.7)	5.5 (0.8)
HbA1c, % (SD)	5.8 (0.2)	5.7 (0.3)	5.7 (0.2)	5.5 (0.4)
Diabetes				
Fasting glucose, mmol/L (SD)	6.2 (1.1)	7.3 (1.6)	6.7 (2.1)	8.2 (2.2)
HbA1c, % (SD)	6.1 (0.6)	7.8 (1.4)	6.4 (0.5)	6.8 (1.3)
Baseline Lipids, mg/dl (SD)				
Triglycerides	181.9 (96.2)	231.5 (127.1)	216.9 (133.8)	210.0 (146.1)
Total cholesterol	181.2 (35.6)	183.7 (46.7)	196.5 (38.6)	187.0 (36.0)
LDL cholesterol	97.6 (36.9)	95.3 (38.8)	110.7 (36.3)	104.7 (29.6)
HDL cholesterol	47.1 (7.3)	43.1 (10.2)	45.4 (8.4)	47.0 (6.7)
Blood pressure, mmHg (SD)				
Systolic	122.7 (10.3)	128.6 (16.0)	123.8 (17.4)	127.6 (9.9)
Diastolic	79.4 (6.0)	79.4 (9.5)	77.0 (10.9)	82.4 (8.7)
Heart rate, bpm (SD)	69.2 (9.4)	71.3 (10.3)	70.5 (9.4)	72.3 (8.2)
CAP score, dB/m (SD)	342.0 (30.3)	332.9 (34.1)	347.2 (37.3)	347.1 (32.6)
PRO-C3, ng/ml (SD)	37.5 (6.9)	36.8 (8.3)	36.7 (7.3)	35.1 (6.3)
ELF score, (SD)	8.9 (0.6)	8.6 (0.6)	8.7 (0.7)	9.0 (1.1)
FibroScan VCTE, kPa (SD)	6.7 (0.9)	6.9 (2.0)	6.1 (1.3)	6.5 (1.8)
cT1, ms (SD)∗	933.4 (114.7)	892.1 (96.3)	909.4 (162.0)	933.7 (21.9)

Data are presented as n (%) for categorical variables and mean (SD) for continuous variables. ∗Sub-study of randomized participants. Baseline participant numbers: placebo (n = 7), 1.2 mg (n = 7), 1.8 mg (n = 5), 2.4 mg (n = 3). ALT, alanine aminotransferase; AST, aspartate aminotransferase; CAP, controlled attenuation parameter; cT1, corrected T1; ELF, enhanced liver fibrosis; HbA1c, hemoglobin A1c; PRO-C3, pro-peptide of type III collagen; VCTE, vibration-controlled transient elastography.

### Primary endpoint

**Fig. 2:**
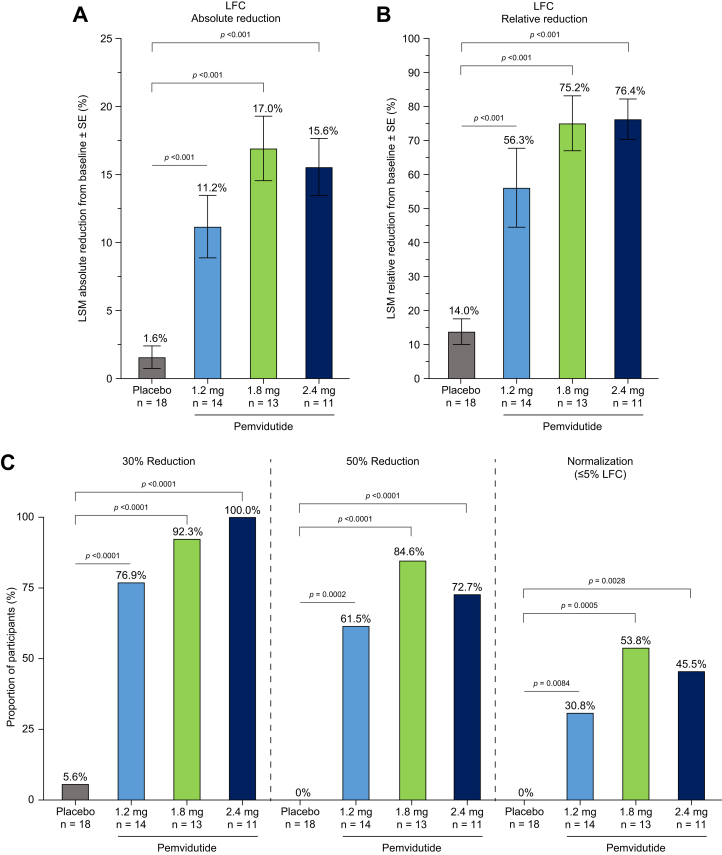
Primary efficacy endpoint after 24 weeks of treatment. (A) LSM (SE) absolute reduction from baseline in LFC; (B) LSM (SE) relative reduction from baseline in LFC; (C) proportion of participants with relative reductions from baseline in LFC of ≥30%, ≥50%, or normalization (≤5%). Statistical significance in (A) and (B) was assessed by ANCOVA. Statistical significance in (C) was assessed by Cochran–Mantel–Haenszel analysis. LFC, liver fat content; LSM, least squares mean.

The primary efficacy endpoint for this trial, a reduction in absolute and relative LFC by MRI-PDFF, was achieved. Compared with baseline values before commencing the parent trial, all pemvidutide-treated groups achieved statistically significant reductions in LFC compared with placebo following 24 weeks of treatment (Week 12 of the extension trial). Absolute reductions in LFC for pemvidutide-treated groups were (LSM [95% CI; *p* value *vs.* placebo]) (1.2 mg) 11.2% [-16.2 to -6.2; *p* <0.001], (1.8 mg) 17.0% [-21.2 to -12.7; *p* <0.001], and (2.4 mg) 15.6% [-20.9 to -10.3; *p* <0.001] compared with 1.6% [-5.8 to 2.6] in those receiving placebo, exceeding the findings at Week 12 (Fig. 2A, Table S3). Relative reductions in LFC were (1.2 mg) 56.3% [-78.2 to -34.4; *p* <0.001], (1.8 mg) 75.2% [-93.8 to -56.7; *p* <0.001], and (2.4 mg) 76.4% [-99.7 to -53.2; *p* <0.001] *vs.* 14.0% [-32.2 to 4.3] in participants receiving placebo (Fig. 2B), likewise exceeding the findings at Week 12 (Table S3). Responder analyses showed the proportions of participants with ≥30% relative reduction in LFC following 24 weeks of pemvidutide treatment were (1.2 mg) 76.9%, (1.8 mg) 92.3%, and (2.4 mg) 100.0% *vs.* 5.6% in placebo (*p* <0.0001 *vs.* placebo, respectively). Furthermore, 61.5% (*p* = 0.0002), 84.6% (*p* <0.0001), and 72.7% (*p* <0.0001) of participants at the 1.2 mg, 1.8 mg, and 2.4 mg dose groups of pemvidutide, respectively, achieved a ≥50% reduction in liver fat compared with no participants in placebo. Normalization of liver fat, defined as a post-treatment liver fat fraction of 5% or less, was achieved in 30.8% (1.2 mg; *p* = 0.0084), 53.8% (1.8 mg; *p* = 0.0005), and 45.5% (2.4 mg; *p* = 0.0028) of participants compared with no participants in placebo after 24 weeks of treatment (Fig. 2C). Changes in LFC were accompanied by relative reductions in liver volume of 12.6% [-20.4 to -4.7; *p* = 0.011], 19.1% [-25.8 to -12.4; *p* <0.001], and 18.0% [-26.3 to -9.7; *p* <0.001] at the 1.2 mg, 1.8 mg, and 2.4 mg doses of pemvidutide, respectively, compared with a 3.3% [-9.9 to 3.3] relative reduction in liver volume in participants receiving placebo (Fig. S1, Table S3).

### Key secondary endpoints

**Fig. 3:**
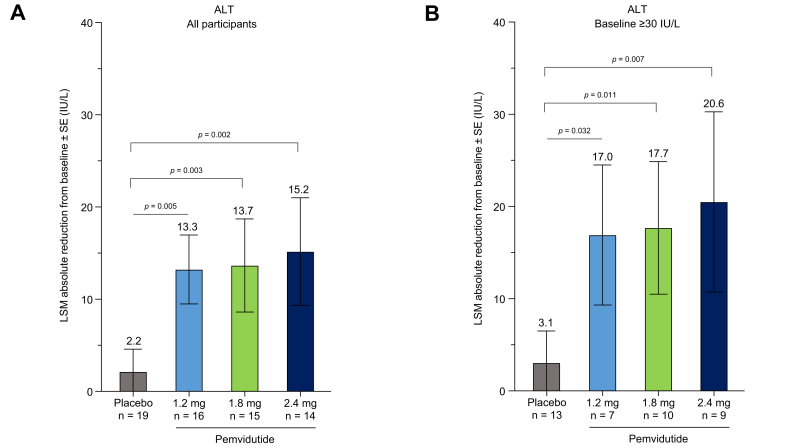
Changes in serum ALT after 24 weeks of treatment. (A) LSM (SE) absolute reduction from baseline in ALT; (B) LSM (SE) absolute ALT reduction in participants with a baseline ALT ≥30 IU/L; Statistical significance was assessed by MMRM. ALT, alanine aminotransferase; LSM, least squares mean; MMRM, mixed model of repeated measures.

In addition to reductions in LFC, participants treated with pemvidutide for 24 weeks exhibited significant improvements in non-invasive biomarkers of hepatic inflammation compared to baseline values. ALT levels were significantly reduced in all pemvidutide groups [1.2 mg (-13.3 [-20.0 to -6.6; *p* = 0.005]), 1.8 mg (-13.7 IU/L [-20.2 to -7.3; *p* = 0.003]), 2.4 mg (-15.2 IU/L [-22.6 to -7.9; *p* = 0.002])] compared with placebo (-2.2 IU/L [-8.0 to 3.6]) (Fig. 3A, Table 2, Table S4). In a sub-analysis of participants with elevated baseline ALT levels ≥30 IU/L, pemvidutide treatment reduced ALT by at least 17 IU/L in all pemvidutide treatment groups (Fig. 3B). Similar reductions were seen in aspartate aminotransaminase (AST) (Table 2). As expected for a MASLD trial population selected for an absence of marked liver fibrosis, significant changes in ELF and Pro-C3 were not observed in this MASLD population; however, pemvidutide treatment did result in a significant decrease in liver stiffness measurement at the 2.4 mg dose (Table 2).

**Table 2:** LSM change in key endpoints following 24 weeks of treatment.

Endpoint	LSM (95% CI)				LSM difference *vs.* placebo (95% CI; *p* value)		
	Placebo (n = 19)	Pemvidutide 1.2 mg (n = 16)	Pemvidutide 1.8 mg (n = 15)	Pemvidutide 2.4 mg (n = 14)	Pemvidutide 1.2 mg	Pemvidutide 1.8 mg	Pemvidutide 2.4 mg
LFC, % absolute∗	-1.6 (-5.8 to 2.6)	-11.2 (-16.2 to -6.2)	-17.0 (-21.2 to -12.7)	-15.6 (-20.9 to -10.3)	-9.6 (-14.1 to -5.1; <0.001)	-15.3 (-19.5 to -11.1; <0.001)	-14.0 (-18.5 to -9.4; <0.001)
LFC, % relative∗	-14.0 (-32.2 to 4.3)	-56.3 (-78.2 to -34.4)	-75.2 (-93.8 to -56.7)	-76.4 (-99.7 to -53.2)	-42.3 (-61.9 to -22.7; <0.001)	-61.3 (-79.6 to -42.9; <0.001)	-62.5 (-82.3 to -42.6; <0.001)
Weight loss, %^†^	-1.4 (-3.5 to 0.7)	-5.1 (-7.3 to -2.8)	-6.2 (-8.4 to -3.9)	-5.2 (-7.8 to -2.6)	-3.7 (-6.1 to -1.3; 0.003)	-4.8 (-7.3 to -2.3; <0.001)	-3.9 (-6.4 to -1.3; 0.003)
Non-diabetes							
Fasting glucose, mmol/L†	-0.2 (-0.6 to 0.2)	0.0 (-0.4 to 0.4)	0.0 (-0.5 to 0.4)	0.0 (-0.5 to 0.4)	0.2 (-0.2 to 0.7; 0.334)	0.2 (-0.4 to 0.7; 0.533)	0.2 (-0.4 to 0.7; 0.533)
HbA1c, %∗	-0.1 (-0.3 to 0.2)	0.1 (-0.1 to 0.3)	0.1 (-0.2 to 0.3)	-0.1 (-0.4 to 0.2)	0.2 (-0.1 to 0.4; 0.134)	0.1 (-0.1 to 0.4; 0.353)	0.0 (-0.3 to 0.2; 0.813)
Diabetes							
Fasting glucose, mmol/L†	-1.7 (-4.2 to 0.8)	1.0 (-2.7 to 4.7)	-0.2 (-2.6 to 2.2)	-1.1 (-4.5 to 2.3)	2.7 (-2.0 to 7.4; 0.251)	1.5 (-1.2 to 4.2; 0.265)	0.6 (-3.1 to 4.4; 0.733)
Lipids							
Triglycerides, mg/dl∗	-69.8 (-133.4 to -6.1)	-71.0 (-144.4 to 2.4)	-74.1 (-138.2 to -10.1)	-105.0 (-184.2 to -25.8)	-1.2 (-68.0 to 65.6; 0.971)	-4.4 (-68.3 to 59.6; 0.891)	-35.2 (-102.3 to 31.9; 0.296)
Total cholesterol, mg/dl∗	-28.1 (-48.2 to -8.1)	-30.0 (-53.3 to -6.6)	-34.9 (-55.5 to -14.4)	-33.6 (-58.6 to -8.6)	-1.9 (-22.9 to 19.2; 0.860)	-6.8 (-27.4 to 13.7; 0.508)	-5.5 (-26.9 to 15.9; 0.607)
LDL cholesterol, mg/dl∗	-11.7 (-27.2 to 3.8)	-7.5 (-26.3 to 11.3)	-18.3 (-34.2 to -2.5)	-13.8 (-33.9 to 6.4)	4.2 (-13.1 to 21.5; 0.626)	-6.6 (-22.3 to 9.1; 0.398)	-2.1 (-19.0 to 14.9; 0.808)
HDL cholesterol, mg/dl∗	-4.0 (-7.8 to -0.2)	-3.7 (-8.0 to 0.7)	-6.3 (-10.1 to -2.5)	-5.3 (-9.9 to -0.6)	0.4 (-3.6 to 4.4; 0.850)	-2.2 (-6.0 to 1.5; 0.233)	-1.2 (-5.2 to 2.7; 0.530)
Blood pressure							
Systolic, mmHg†	-2.3 (-7.6 to 3.0)	-10.1 (-16.3 to -3.9)	-5.5 (-11.6 to 0.6)	-12.0 (-18.7 to -5.2)	-7.8 (-15.5 to -0.1; 0.047)	-3.2 (-10.9 to 4.5; 0.409)	-9.7 (-17.8 to -1.6; 0.019)
Diastolic, mmHg†	-2.5 (-6.4 to 1.4)	-2.9 (-7.4 to 1.6)	-4.0 (-8.4 to 0.4)	-3.8 (-8.7 to 1.1)	-0.4 (-6.0 to 5.2; 0.898)	-1.5 (-7.1 to 4.1; 0.600)	-1.3 (-7.2 to 4.6; 0.671)
Heart rate, bpm†	-1.0 (-4.4 to 2.5)	3.7 (-0.4 to 7.7)	0.5 (-3.4 to 4.5)	-0.1 (-4.5 to 4.3)	4.6 (-0.4 to 9.7; 0.072)	1.5 (-3.5 to 6.6; 0.557)	0.8 (-4.5 to -6.2; 0.757)
ALT, IU/L†	-2.2 (-8.0 to 3.6)	-13.3 (-20.0 to -6.6)	-13.7 (-20.2 to -7.3)	-15.2 (-22.6 to -7.9)	-11.1 (-18.7 to -3.4; 0.005)	-11.5 (-19.2 to -3.9; 0.003)	-13.0 (-21.1 to -5.0; 0.002)
AST, IU/L†	1.9 (-1.4 to 5.1)	-6.9 (-10.7 to -3.2)	-6.6 (-10.3 to -3.0)	-6.6 (-10.7 to -2.5)	-8.8 (-13.3 to -4.3; <0.001)	-8.5 (-13.0 to -4.0; <0.001)	-8.4 (-13.1 to -3.7; <0.001)
CAP score, dB/m∗	-32.1 (-61.7 to -2.5)	-36.5 (-70.8 to -2.3)	-52.9 (-83.0 to -22.9)	-90.0 (-126.7 to -53.3)	-4.5 (-34.1 to 25.2; 0.764)	-20.8 (-49.9 to 8.3; 0.156)	-57.9 (-88.9 to -26.9; <0.001)
PRO-C3, ng/ml∗	-6.4 (-12.1 to -0.7)	-8.2 (-14.9 to -1.5)	-4.2 (-10.2 to 1.6)	-12.2 (-19.4 to -5.0)	-1.8 (-7.8 to 4.2; 0.552)	2.2 (-3.6 to 7.9; 0.447)	-5.8 (-12.0 to 0.4; 0.067)
ELF score∗	0.3 (-0.1 to 0.7)	0.1 (-0.4 to 0.6)	0.0 (-0.4 to 0.4)	0.4 (-0.1 to 0.9)	-0.2 (-0.6 to 0.3; 0.451)	-0.3 (-0.7 to 0.2; 0.202)	0.1 (-0.3 to 0.6; 0.641)
FibroScan VCTE, kPa∗	0.3 (-1.1 to 1.7)	-1.1 (-2.7 to 0.6)	0.0 (-1.4 to 1.5)	-1.4 (-3.1 to 0.4)	-1.4 (-2.8 to 0.1; 0.062)	-0.3 (-1.7 to 1.1; 0.683)	-1.7 (-3.2 to -0.2; 0.027)

LSM HbA1c for patients with diabetes was not calculated because of low participant number. Raw HbA1c mean changes (95% CI) from baseline of 0.3 (-1.0 to 1.6), -1.3 (-1.3 to 0.0), 0.1 (-0.5 to 0.6), and -0.3 (-4.1 to 3.5) for placebo, 1.2 mg, 1.8 mg, and 2.4 mg pemvidutide, respectively, were measured in this cohort.

ALT, alanine aminotransferase; AST, aspartate aminotransferase; CAP, controlled attenuation parameter; ELF, enhanced liver fibrosis; HbA1c, hemoglobin A1c; HDL, high-density lipoprotein; LDL, low-density lipoprotein; LFC, liver fat content; LSM, least square means; PRO-C3, pro-peptide of type III collagen; VCTE, vibration-controlled transient elastography.

∗ Based on ANCOVA model.

† Based on MMRM model.

**Fig. 4:**
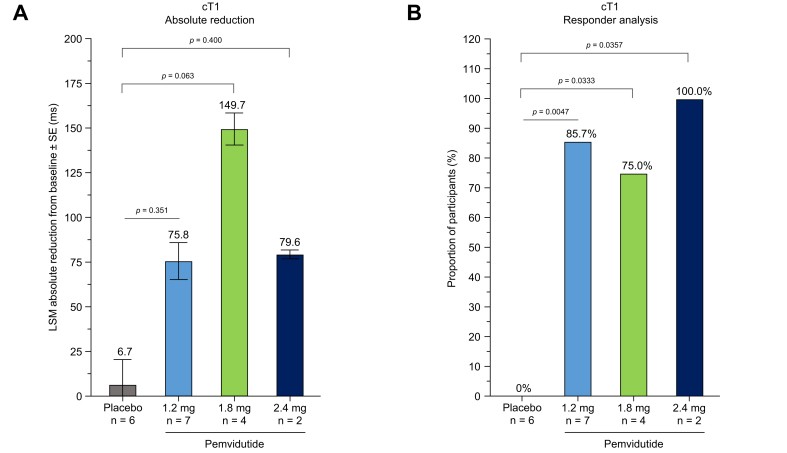
Sub-analysis of cT1 after 24 weeks of treatment. (A) LSM (SE) absolute reduction in cT1 relaxation time; (B) proportion of participants with ≥80 ms reduction in cT1 relaxation time. Statistical significance in (A) was assessed by ANCOVA. Statistical significance in (B) was assessed by Fisher’s exact test. cT1, iron-corrected T1; LSM, least squares mean.

Participants analyzed in a sub-study of cT1 imaging showed absolute changes in cT1 relaxation time across pemvidutide treatment groups of -75.8 ms [-215.0 to 63.3; *p* = 0.351], -149.7 ms [-292.9 to -6.6; *p* = 0.063], and -79.6 ms [-262.5 to 103.4; *p* = 0.400] at the 1.2 mg, 1.8 mg, and 2.4 mg doses of pemvidutide, respectively, *vs.* a decrease of 6.7 ms [-95.1 to 81.8] in the placebo group (Fig. 4A, Table S4). A cT1 responder analysis by treatment group showed 85.7% (*p* = 0.0047), 75.0% (*p* = 0.0333), and 100.0% (*p* = 0.0357) achieved ≥80 ms reductions in relaxation time at the 1.2 mg, 1.8 mg, and 2.4 mg doses of pemvidutide, respectively, after 24 weeks of pemvidutide treatment (Week 12 of the extension trial). No participants in the placebo group achieved an 80 ms reduction in cT1 relaxation time (Fig. 4B).

**Fig. 5:**
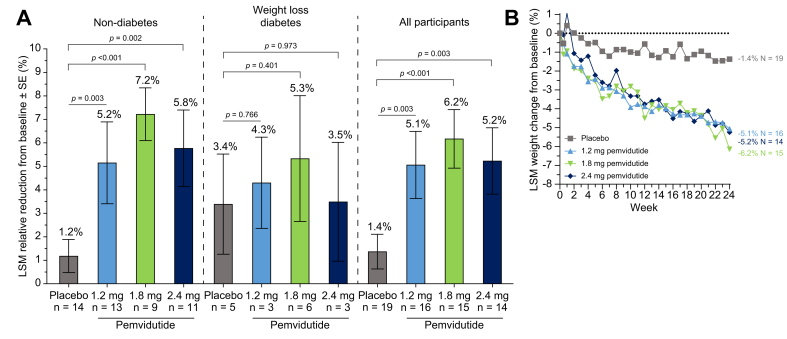
Percent reduction in body weight after 24 weeks of treatment. LSM (SE) percent reductions from baseline at (A) Week 24 and (B) over time. Statistical significance in (A) was assessed by MMRM. LSM, least squares mean; MMRM, mixed model of repeated measures.

Significant weight loss compared to placebo was observed in all pemvidutide dose groups following 24 weeks of pemvidutide treatment (Fig. 5A, Table S4). Reductions in body weight from baseline to Week 24 were 5.1% [-7.3 to -2.8; *p* = 0.003], 6.2% [-8.4 to -3.9; *p* <0.001], and 5.2% [-7.8 to -2.6; *p* = 0.003] for 1.2 mg, 1.8 mg, and 2.4 mg groups, respectively, compared with 1.4% [-3.5 to 0.7] for those receiving placebo. Weight losses were greater in participants without diabetes mellitus, in whom changes of (1.2 mg) 5.2% [-7.5 to -2.8; *p* = 0.003], (1.8 mg) 7.2% [-9.8 to -4.7; *p* <0.001], and (2.4 mg) 5.8% [-8.6 to -3.0; *p* = 0.002] were observed compared with 1.2% [-3.4 to 1.1] in placebo. Weight loss trajectories suggested that weight loss would continue beyond 24 weeks of treatment (Fig. 5B).

### Safety

Pemvidutide remained well-tolerated throughout the 12 weeks of the extension trial, with two severe AEs that were deemed unrelated to study medication (Table 3). One participant in the 1.2 mg dose group had *Salmonella* infection that led to trial discontinuation and one participant in the 1.8 mg dose group exhibited hypertension more than 3 weeks after the last pemvidutide dose. A severe AE was noted in one participant in the placebo group that had chest pain following coronary stent placement. Two (12.5%) participants in the 1.2 mg pemvidutide dose group and one (6.7%) participant in the 1.8 mg pemvidutide dose group discontinued treatment because of AEs. The most common AE during the extension phase was nausea, which was observed in 33.3% of pemvidutide-treated participants, with the majority of events being mild-to-moderate. A small number of participants reported diarrhea and/or constipation that was mild-to-moderate in nature and resolved without treatment. Pemvidutide treatment improved systolic and diastolic BP at all three dose levels (Table 2). Importantly, no significant changes in heart rate were observed.

**Table 3:** Adverse events.

Adverse event	Placebo (n = 19)	Pemvidutide 1.2 mg (n = 16)	Pemvidutide 1.8 mg (n = 15)	Pemvidutide 2.4 mg (n = 14)
AEs related to study drug, n (%)	8 (42.1)	8 (50.0)	12 (80.0)	8 (57.1)
AEs leading to study discontinuation, n (%)	0	0	0	0
AEs leading to treatment discontinuation, n (%)	0	2 (12.5)	1 (6.7)	0
Severe AEs, n (%)	1 (5.3)∗	1 (6.3)†	1 (6.7)‡	0
SAEs, n (%)	0	0	0	0
Nausea, n (%)				
Mild	2 (10.5)	2 (12.5)	3 (20.0)	4 (28.6)
Moderate	0	1 (6.3)	4 (26.7)	1 (7.1)
Vomiting, n (%)				
Mild	0	1 (6.3)	1 (6.7)	1 (7.1)
Moderate	0	0	0	0
Diarrhea, n (%)				
Mild	4 (21.1)	2 (12.5)	5 (33.3)	1 (7.1)
Moderate	0	1 (6.3)	0	0
Constipation, n (%)				
Mild	0	2 (12.5)	3 (20.0)	0
Moderate	1 (5.3)	1 (6.3)	0	0

∗ Chest pain post elective coronary stent placement.

† *Salmonella* infection.

‡ Hypertension >3 weeks post last dose of study medication. AEs, adverse events; SAEs, serious AEs.

## Discussion

This trial was a 12-week extension of a randomized, double-blind, placebo-controlled, 12-week parent clinical trial conducted to evaluate the safety, tolerability, and pharmacodynamic effects of pemvidutide in adults with MASLD and overweight or obesity, with or without T2DM. Twenty-four (24) weeks of treatment with pemvidutide led to significant reductions in LFC, hepatic inflammation (serum ALT, cT1 relaxation time), and body weight, with continued improvement upon the LFC effects, and general improvements in ALT, cT1, and weight loss, observed after 12 weeks of treatment.

Statistically significant decreases in relative LFC of greater than 75% were noted in both the 1.8 mg and 2.4 mg pemvidutide groups following 24 weeks of treatment, establishing the efficacy of pemvidutide. By week 24, 100% of participants in the 2.4 mg pemvidutide group achieved a 30% reduction in LFC, and >61% of participants in each group had a 50% relative reduction in LFC. Clinical studies have shown that a relative reduction in LFC of ≥30% is associated with a ≥2-point improvement in NAFLD activity score (NAS), and a ≥50% relative reduction in LFC correlates with MASH resolution and improvements in fibrosis.[18], [19], [20] Given the potent effects of all three pemvidutide doses on LFC at 24 weeks, continued treatment may result in even greater LFC reduction and higher rates of liver fat normalization.

The reductions in LFC shown here surpass those seen with GLP-1R mono-agonists and gastric inhibitory peptide receptor (GIPR/GLP-1R) dual-agonist compounds with similar or greater levels of weight loss, suggesting a pivotal role for glucagon in lowering LFC.^7^^,^[21], [22], [23], [24] The known direct effects of glucagon on hepatic fatty acid β-oxidation and lipogenesis likely worked in concert with GLP-1 to accelerate the reduction in LFC.^10^

The reduction in LFC was accompanied by decreases in serum biomarkers of liver inflammation such as ALT and AST. The reduction in serum ALT occurred despite the baseline values not being substantially above the normal range. Importantly, in participants with a baseline ALT ≥30 IU/L, absolute reductions of at least 17 IU/L were observed following pemvidutide treatment for 24 weeks. ALT reductions ≥17 IU/L have been proposed as a predictor of histologic response in patients with MASH.^25^ When combined with the improvement in liver stiffness measurements described in this trial, these changes may be indicative of histopathological improvements that will need to be confirmed in a biopsy-driven trial.

Participants treated with pemvidutide for 24 weeks also had clinically meaningful reductions in cT1 relaxation time. As an indicator of regional tissue water content, cT1 imaging reflects the degree of hepatic inflammatory disease and extracellular collagen matrix. A reduction of cT1 ≥80 ms is associated with a 2-point reduction in NAS.^26^ Furthermore, elevated cT1 levels are associated with increased risk of major adverse cardiac events and major adverse liver outcomes independent of LFC.^27^^,^^28^ In this trial, the mean cT1 value at baseline across all groups was ≥914 ms and all pemvidutide-treated groups achieved values <800 ms by Week 24. Overall, 84.6% of measured participants achieved a ≥80 ms reduction in cT1 from baseline, with all participants in the 2.4 mg group having a ≥80 ms decrease.

Finally, body weight loss is correlated with MASH improvement. In this trial, all pemvidutide-treated groups achieved statistically significant weight loss by Week 24 without prescribed lifestyle or dietary modification. This is differentiated from non-incretin-based MASH therapeutics that are either approved or in development that do not yield clinically significant decreases in body weight or even cause weight gain.[29], [30], [31] The incretin-based approaches for MASH elicit significant weight loss, but the reduction in LFC with some of these agents appears less robust than the data reported here.^7^^,^^23^^,^^32^^,^^33^ The inclusion of glucagon receptor agonism likely augments the reduction in LFC in participants with MASLD as noted in this trial and results reported elsewhere.^33^

A limitation in these data may occur in the extrapolation to a MASH cohort as, by design, participants with advanced fibro-inflammatory disease were excluded from this trial, preventing a direct investigation of the effect of pemvidutide in this patient population. In addition, not all of the original 94 participants in the parent trial participated in the extension trial, although analyses demonstrated that the treatment groups were comparable.

In conclusion, pemvidutide led to rapid and potent reductions in LFC, serum ALT, serum AST, cT1, and body weight at 24 weeks. These findings are expected to be predictive of histopathological improvements in MASH and other comorbidities of obesity in late-phase clinical studies.

## Abbreviations

AE, adverse event; ALT, alanine aminotransferase; ANCOVA, analysis of covariance; AST, aspartate aminotransaminase; BP, blood pressure; CAP, controlled attenuation parameter; cT1, iron-corrected T1; ECG, electrocardiogram; ELF®, enhanced liver fibrosis; GCGR, G-coupled glucagon receptor; GIPR, gastric inhibitory polypeptide receptor; GLP-1, glucagon-like peptide-1; GLP-1R, glucagon-like peptide-1 receptor; HbA1c, glycated hemoglobin; LFC, liver fat content; LSM, least squares mean; MASH, metabolic dysfunction-associated steatohepatitis; MASLD, metabolic dysfunction-associated steatotic liver disease; MMRM, mixed model of repeated measures; MRI, magnetic resonance imaging; MRI-PDFF, magnetic resonance imaging proton density fat fraction; NAS, NAFLD activity score; Pro-C3, pro-peptide of type III collagen; SGLT-2, sodium glucose cotransporter-2; T2DM, type 2 diabetes mellitus; VCTE, vibration-controlled transient elastography.

## Financial support

This trial was funded by Altimmune, Inc. The authors (some of whom are Altimmune, Inc. employees) designed, collected, analyzed, and interpreted the data. All authors had full access to the trial data, and the corresponding author had final responsibility for publishing the manuscript.

## Authors’ contributions

Study design: SKB, MSH. Data analysis and interpretation: all authors. Writing – drafting/reviewing/editing: all authors.

## Data availability

Aggregated data that supports the findings of this trial may be available from the authors on reasonable request pending approval from Altimmune, Inc. Individual participant-level data containing confidential or identifiable participant information is covered by patient privacy and cannot be shared.

## Conflicts of interest

SKB, JJS, ST, JAG, JY, BG, MSR, and MSH are employees of Altimmune, Inc. and hold a financial interest in the company.

Please refer to the accompanying ICMJE disclosure forms for further details.
