# Supplementary material for: Safety and efficacy of 24 weeks of pemvidutide in metabolic dysfunction-associated steatotic liver disease: A randomized, controlled clinical trial
Source: JHEP Rep. 2025 Jun 18;7(11):101483. doi: 10.1016/j.jhepr.2025.101483 (PMC12529369; doi:10.1016/j.jhepr.2025.101483)
Supplement: Multimedia component 1 [file mmc1.pdf]

Safety and efficacy of 24 weeks of pemvidutide in metabolic dysfunction-associated steatotic liver disease: A randomized, controlled clinical trial

Sarah K. Browne, John J. Suschak, Shaheen Tomah, Julio A. Gutierrez, Jay Yang, M. Scot M. Roberts, M. Scott Harris

Table of contents

Fig. S1……………………………………………………………………………………2

Table S1…………………………………………………………………………………3

Table S2…………………………………………………………………………………4

Table S3…………………………………………………………………………………5

Table S4…………………………………………………………………………………6

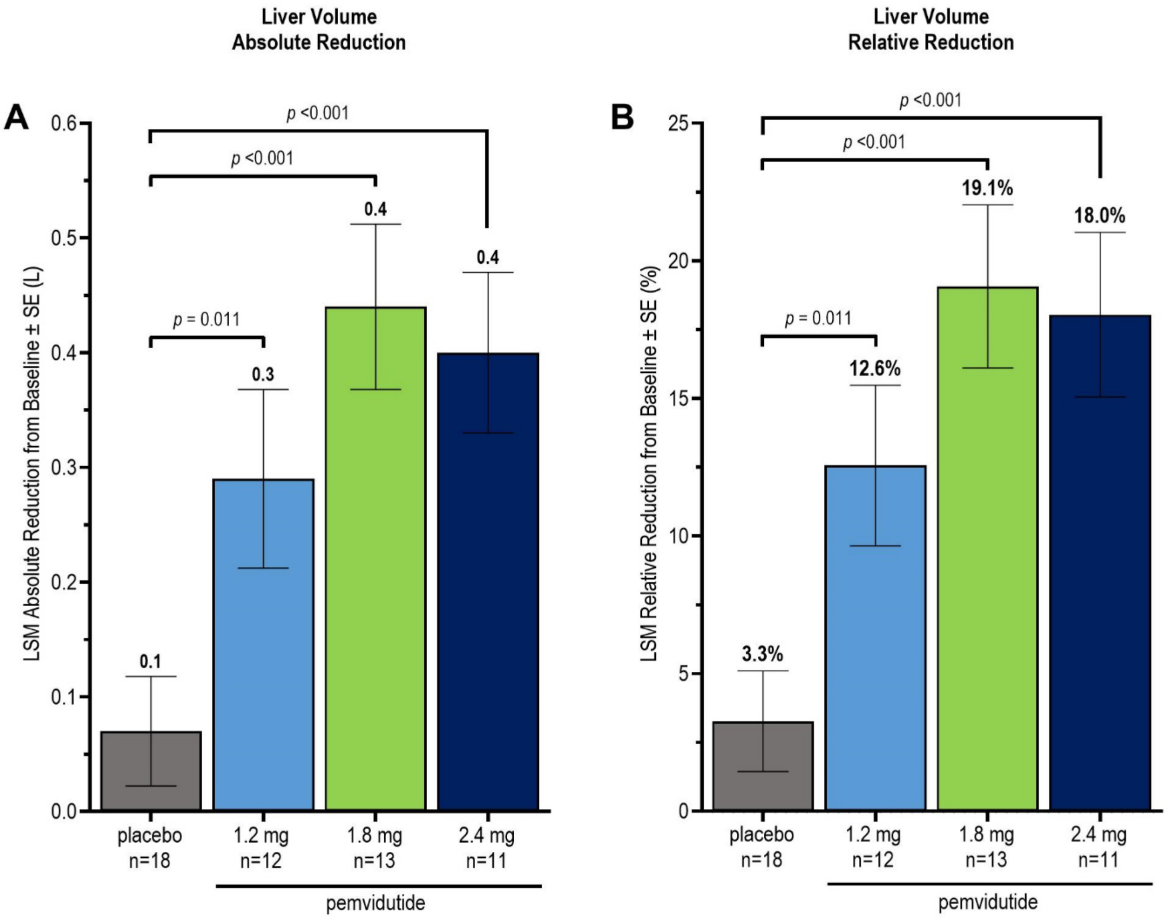

Fig. S1. Changes in liver volume at Week 24. (A) LSM (SE) absolute reduction from baseline in liver volume; (B) LSM (SE) relative reduction from baseline in liver volume. Statistical significance was assessed by ANCOVA.

Table S1: Comparison of responses following 12 weeks of treatment

Characteristic	LSM (95% CI)							
	Did not Extend Treatment to 24 Weeks				Extended Treatment to 24 Weeks			
	placebo (n=4)	1.2 mg pemvidutide (n=5)	1.8 mg pemvidutide (n=2)	2.4 mg pemvidutide (n=6)	placebo (n=19)	1.2 mg pemvidutide (n=16)	1.8 mg pemvidutide (n=15)	2.4 mg pemvidutide (n=14)
**LFC,** % absolute[a]	5.5 (-3.0 to 14.1)	-5.0 (-14.6 to 4.5)	-14.7 (-24.8 to -4.6)	-8.9 (-17.4 to -0.4)	-1.3 (-4.6 to 1.9)	-7.5 (-11.1 to -3.9)	-16.1 (-19.5 to -12.8)	-12.6 (-16.6 to -8.5)
**LFC,** % relative[a]	17.7 (-30.7 to 66.0)	-24.8 (-78.9 to 29.2)	-85.5 (-142.7 to -28.2)	-45.1 (-93.2 to 2.9)	-11.4 (-26.2 to 3.4)	-42.4 (-59.0 to -25.9)	-71.5 (-86.7 to -56.4)	-63.7 (-82.0 to -45.4)
**ALT,** IU/L[b]	-8.0 (-28.6 to 12.5)	-7.1 (-29.8 to 15.6)	-22.8 (-47.6 to 2.1)	-15.5 (-34.6 to 3.7)	-7.1 (-12.1 to -2.0)	-12.0 (-17.5 to -6.5)	-12.2 (-17.6 to -6.8)	-15.3 (-21.2 to -9.4)
**Weight loss,** %[b]	-1.5 (-5.4 to 2.4)	-2.9 (-7.0 to 1.2)	-4.7 (-8.9 to -0.4)	-3.1 (-6.9 to 0.8)	-0.0 (-1.5 to 1.5)	-3.2 (-4.8 to -1.6)	-4.0 (-5.6 to -2.5)	-3.1 (-4.8 to -1.3)

ALT, alanine aminotransferase; LFC, liver fat content; LSM, least square means.
[a]Based on ANCOVA model.
[b]Based on MMRM model.

Table S2: Baseline demographics for participants in the parent and extension trials[1]

Characteristic	Treatment			
	placebo	1.2 mg pemvidutide	1.8 mg pemvidutide	2.4 mg pemvidutide
**Age,** mean years (SD)				
Parent trial	47.9 (14)	48.6 (11)	50.3 (9)	48.8 (8)
Extension trial	49.0 (15)	48.6 (11)	49.9 (10)	48.4 (8)
**Sex**, n (% female)				
Parent trial	14 (58.3)	9 (39.1)	12 (52.2)	15 (62.5)
Extension trial	11 (57.9)	7 (43.8)	8 (53.3)	8 (57.1)
**Ethnicity**, n (% Hispanic)				
Parent trial	14 (58.3)	20 (87.0)	19 (82.6)	18 (75.0)
Extension trial	11 (57.9)	15 (93.8)	12 (80.0)	9 (64.3)
**Body Weight**, kg (SD)				
Parent trial	105.1 (20.8)	102.4 (14.6)	98.9 (19.7)	98.2 (18.9)
Extension trial	104.4 (21.2)	101.4 (16.3)	100.9 (13.2)	107.4 (17.2)
**BMI**, kg/m^2 (SD)				
Parent trial	36.9 (4.7)	36.3 (5.6)	35.4 (3.9)	35.3 (5.0)
Extension trial	37.1 (4.9)	36.7 (6.1)	36.0 (3.8)	37.0 (5.3)
**LFC**, % (SD)				
Parent trial	23.8 (9.2)	21.6 (7.3)	21.8 (8.0)	20.2 (7.0)
Extension trial	24.0 (9.6)	20.1 (7.7)	23.9 (7.4)	20.5 (6.5)
**ALT**, IU/L (SD)				
Parent trial	39.5 (21.4)	32.4 (13.8)	36.4 (15.6)	37.8 (24.4)
Extension trial	41.0 (21.3)	32.4 (14.2)	35.3 (13.0)	39.6 (26.6)
**AST**, IU/L (SD)				
Parent trial	23.8 (10.0)	25.4 (7.5)	24.6 (7.2)	27.2 (13.9)
Extension trial	25.1 (10.5)	24.4 (6.7)	23.6 (7.0)	29.4 (15.5)
**Diabetes status**, n (% type 2 diabetes)				
Parent trial	6 (25.0)	7 (30.4)	7 (30.4)	7 (33.3)
Extension trial	5 (26.3)	3 (18.8)	6 (40.0)	3 (21.4)

[1]Shown are the participant baseline demographics for parent 12-week trial (n=94) and the subset of participants (n=64) in the extension trial. All values are for baseline of the parent trial.

ALT, alanine aminotransferase; AST, aspartate aminotransferase;

Supplementary Table 3: LSM change from baseline in liver fat content and liver volume (data from the 12-week parent and 24-week extension trials for the participants of the extension trial)

Endpoint	LSM (95% CI)				LSM difference vs. placebo (95% CI; *p* value)		
	placebo (n=19)	1.2 mg pemvidutide (n=16)	1.8 mg pemvidutide (n=15)	2.4 mg pemvidutide (n=14)	1.2 mg pemvidutide	1.8 mg pemvidutide	2.4 mg pemvidutide
**LFC,** % absolute[a]							
Week 12	-1.3 (-4.6 to 1.9)	-7.5 (-11.1 to -3.9)	-16.1 (-19.5 to -12.8)	-12.6 (-16.6 to -8.5)	-6.2 (-9.4 to -3.0; <0.001)	-14.8 (-18.1 to -11.5; <0.001)	-11.3 (-14.6 to -7.9; <0.001)
Week 24	-1.6 (-5.8 to 2.6)	-11.2 (-16.2 to -6.2)	-17.0 (-21.2 to -12.7)	-15.6 (-20.9 to -10.3)	-9.6 (-14.1 to -5.1; <0.001)	-15.3 (-19.5 to -11.1; <0.001)	-14.0 (-18.5 to -9.4; <0.001)
**LFC,** % relative[a]							
Week 12	-11.4 (-26.2 to 3.4)	-42.4 (-59.0 to -25.9)	-71.5 (-86.7 to -56.4)	-63.7 (-82.0 to -45.4)	-31.0 (-45.6 to -16.5; <0.001)	-60.1 (-74.9 to -45.3; <0.001)	-52.3 (-67.5 to -37.1; <0.001)
Week 24	-14.0 (-32.2 to 4.3)	-56.3 (-78.2 to -34.4)	-75.2 (-93.8 to -56.7)	-76.4 (-99.7 to -53.2)	-42.3 (-61.9 to -22.7; <0.001)	-61.3 (-79.6 to -42.9; <0.001)	-62.5 (-82.3 to -42.6; <0.001)
**Liver volume,** L absolute[a]							
Week 12	0.0 (-0.1 to 0.2)	-0.2 (-0.3 to -0.0)	-0.4 (-0.6 to -0.3)	-0.3 (-0.5 to -0.2)	-0.2 (-0.3 to -0.1; <0.001)	-0.5 (-0.6 to -0.4; <0.001)	-0.4 (-0.5 to -0.2; <0.001)
Week 24	-0.1 (-0.2 to 0.1)	-0.3 (-0.5 to -0.1)	-0.4 (-0.6 to -0.3)	-0.4 (-0.6 to -0.2)	-0.2 (-0.4 to -0.1; 0.011)	-0.4 (-0.5 to -0.2; <0.001)	-0.3 (-0.5 to -0.2; <0.001)
**Liver volume,** % relative[a]							
Week 12	2.0 (-3.5 to 7.4)	-7.4 (-13.3 to -1.4)	-18.0 (-23.6 to -12.5)	-13.8 (-20.4 to -7.2)	-9.3 (-14.5 to -4.1; <0.001)	-20.0 (-25.5 to -14.5; <0.001)	-15.7 (-21.2 to -10.3;<0.001)
Week 24	-3.3 (-9.9 to 3.3)	-12.6 (-20.4 to -4.7)	-19.1 (-25.8 to -12.4)	-18.0 (-26.3 to -9.7)	-9.3 (-16.3 to -2.2; 0.011)	-15.8 (-22.5 to -9.1; <0.001)	-14.8 (-21.9 to -7.7; <0.001)

LFC, liver fat content; LSM, least square means
[a]Based on ANCOVA model.

Supplementary Table 4: LSM change from baseline in key endpoints (data from the 12-week parent and 24-week extension trials for the participants of the extension trial)

Endpoint	LSM (95% CI)				LSM difference vs. placebo (95% CI; *p* value)		
	placebo (n=19)	1.2 mg pemvidutide (n=16)	1.8 mg pemvidutide (n=15)	2.4 mg pemvidutide (n=14)	1.2 mg pemvidutide	1.8 mg pemvidutide	2.4 mg pemvidutide
**ALT,** IU/L[a]							
Week 12	-7.1 (-12.1 to -2.0)	-12.0 (-17.5 to -6.5)	-12.2 (-17.6 to -6.8)	-15.3 (-21.2 to -9.4)	-4.9 (-11.4 to 1.5; 0.131)	-5.2 (-11.6 to 1.3; 0.116)	-8.3 (-14.9 to -1.7; 0.014)
Week 24	-2.2 (-8.0 to 3.6)	-13.3 (-20.0 to -6.6)	-13.7 (-20.2 to -7.3)	-15.2 (-22.6 to -7.9)	-11.1 (-18.7 to -3.4; 0.005)	-11.5 (-19.2 to -3.9; 0.003)	-13.0 (-21.1 to -5.0; 0.002)
**cT1,** ms[b]							
Week 12	5.4 (-32.7 to 43.5)	-77.6 (131.6 to -23.6)	-133.2 (-193.2 to -73.3)	-53.5 (-134.3 to 27.3)	-83.0 (-136.1 to -29.9; 0.006)	-138.7 (-198.1 to -79.2; <0.001)	-58.9 (-135.1 to 17.3; 0.116)
Week 24	-6.7 (-95.1 to 81.8)	-75.8 (-215.0 to 63.3)	-149.7 (-292.9 to -6.6)	-79.6 (-262.5 to 103.4)	-69.2 (-228.3 to 89.9; 0.351)	-143.1 (-295.8 to 9.7; 0.063)	-72.9 (-259.4 to 113.6; 0.400)
**Weight loss**							
Week 12							
CFB, %[a]	-0.0 (-1.5 to 1.5)	-3.2 (-4.8 to -1.6)	-4.0 (-5.6 to -2.5)	-3.1 (-4.8 to -1.3)	-3.2 (-4.9 to -1.5; <0.001)	-4.0 (-5.7 to -2.3; <0.001)	-3.1(-4.8 to -1.3; <0.001)
5% OR	--	--	--	--	18.7 (0.9 to 369.2)	15.3 (0.8 to 310.0)	16.7 (0.8 to 341.3)
Week 24							
CFB, %[a]	-1.4 (-3.5 to 0.7)	-5.1 (-7.3 to -2.8)	-6.2 (-8.4 to -3.9)	-5.2 (-7.8 to -2.6)	-3.7 (-6.1 to -1.3; 0.003)	-4.8 (-7.3 to -2.3; <0.001)	-3.9 (-6.4 to -1.3; 0.003)
5% OR	--	--	--	--	6.6 (1.1 to 38.7)	9.7 (1.6 to 57.7)	6.4 (1.1 to 38.9)
10% OR	--	--	--	--	6.7 (0.3 to 151)	10.9 (0.5 to 229.9)	7.8 (0.4 to 176.3)

ALT, alanine aminotransferase; CFB, change from baseline; LSM, least square means; OR, odds ratio

[a]Based on MMRM model.

[b]Based on ANCOVA model
