# Supplementary material for: Safety and efficacy of 24 weeks of pemvidutide in metabolic dysfunction-associated steatotic liver disease: A randomized, controlled clinical trial
Source: JHEP Rep. 2025 Jun 18;7(11):101483. doi: 10.1016/j.jhepr.2025.101483 (PMC12529369; doi:10.1016/j.jhepr.2025.101483)
Supplement: Multimedia component 4 [file mmc4.zip › alt-801-106_protocol_v4_Clean.pdf]

Altimmune, Inc.
910 Clopper Road, Suite 201 S
Gaithersburg, MD 20878
USA

Clinical Study Protocol

**DRUG:**	ALT-801
**STUDY NUMBER:**	ALT-801-106
**PROTOCOL TITLE:**	A 12-Week Extension Study of ALT-801 in Diabetic and Non-Diabetic Overweight and Obese Subjects with Non-alcoholic Fatty Liver Disease
**SPONSOR:**	Altimmune, Inc.

**Original Protocol (Version 1.0)**	22 December 2021
**Amendment 01 (Version 2.0)**	22 February 2022
**Amendment 02 (Version 3.0)**	30 March 2022
**Amendment 03 (Version 4.0)**	06 May 2022

Confidentiality Statement

The information in this document is confidential, and its contents are the property of Altimmune, Inc., and are confidential. Unauthorized copying, distribution, disclosure, or use is prohibited.

CLINICAL PROTOCOL APPROVAL FORM

Protocol Title: A 12-Week Extension Study of ALT-801 in Diabetic and Non--Diabetic Overweight and Obese Subjects with Non-alcoholic Fatty Liver Disease

Study No: ALT-801-106

Protocol Version No: 4.0

Protocol Version Date: 06 May 2022

This study protocol was subject to critical review and has been approved by the Sponsor representative.

M. Scott Harris
Chief Medical Officer
Altimmune, Inc.
910 Clopper Road
Suite 201S
Gaithersburg, MD 20878

Signature and Date: ____

CONFIDENTIALITY AND INVESTIGATOR STATEMENT

PROTOCOL ALT-801-106

A 12-Week Extension Study of ALT-801 in Diabetic and Non-Diabetic Overweight and Obese Subjects with Non-alcoholic Fatty Liver Disease

The information contained in this protocol and all other information relevant to ALT-801 are the confidential and proprietary information of Altimmune, Inc., and except as may be required by federal, state, or local laws or regulation, may not be disclosed to others without prior written permission of Altimmune, Inc.

I have read the protocol and I agree that it contains all of the necessary information for me and my staff to conduct this study as described. I will conduct this study as outlined herein, in accordance with the regulations stated in the Federal Code of Regulations for Good Clinical Practice (GCP) and International Council for Harmonisation (ICH) guidelines and will make a reasonable effort to complete the study within the time designated.

I will provide all study personnel under my supervision copies of the protocol and any amendments, and access to all information provided by Altimmune, Inc., or specified designees. I will discuss the material with them to ensure that they are fully informed about ALT-801 and the study.

____________________________________ _________________________________

Principal Investigator Name (printed) Signature

__________________________ ___________________

Date (dd-mmm-yyyy) Site Number

STUDY SUMMARY

**Sponsor:** Altimmune, Inc.
**Study Title:** A 12-Week Extension Study of ALT-801 in Diabetic and Non-Diabetic Overweight and Obese Subjects with Non-alcoholic Fatty Liver Disease
**Study Number:** ALT-801-106
**Study Phase:** 1
**Study Center:** Up to approximately 15 study centers in the United States
**Number of Subjects Planned:** Up to approximately 90 overweight and obese diabetic and nondiabetic subjects with nonalcoholic fatty liver disease (NAFLD) who completed the Day 85 visit of Study ALT-801-105
**Duration of Subject Participation:**<br>Approximately 4 months, including an 85-day treatment period and a 25-day follow-up period
**Enrollment Period:** TBD
**Test Product:** ALT-801 or placebo administered by subcutaneous (SC) injection once weekly for up to 12 doses
**Study Objectives:**<br>Safety Objective<br>To assess the safety and tolerability of ALT-801 in subjects with NAFLD.<br>Pharmacodynamic (PD) Objectives<br>To evaluate the effects of ALT-801 on:<br>• Liver fat content<br>• Anthropometric parameters, including body composition<br>• Lipid metabolism<br>• Metabolic markers<br>• Inflammatory markers<br>• Fibrosis markers<br>• Lipotoxicity markers<br>Quality of Life (QoL) Objective<br>To evaluate the effects of ALT-801 on QoL using established QoL instruments.

Study Design:

This is a 12-week extension study to assess the safety and effects of a total of 24 weeks of treatment with ALT-801 on liver fat content, anthropometric parameters and body composition, lipid metabolism, and inflammatory, metabolic, fibrosis, and lipotoxicity markers in diabetic and non-diabetic overweight and obese subjects with NAFLD. Subjects who completed the Day 85 visit with no more than 1 missed dose of investigational product (IP) in Study ALT-801-105, and who continue to meet eligibility criteria will receive 12 additional weeks of the same treatment they received in Study ALT-801-105. Treatment will remain blinded, and neither the investigator nor subject will be aware of the treatment being received.

Informed consent will be obtained at the screening visit for Study ALT-801-106 on Day -7 (coincides with the Day 78 visit of Study ALT-801-105) and the first dose of IP will be administered on Day 1 (coincides with the Day 85 visit of Study ALT-801-105), as indicated in Table 1. Subsequent visits will be conducted at the clinic, home, or work through the Day 85 or early termination visit of this study. Subjects will return for a safety follow-up visit on Day 110.

Counseling on diet and exercise, as provided in Study ALT-801-105, will continue to be provided, as indicated in Table 1.

Investigators will follow the decision criteria for the timing and method of intervention in subjects who develop worsening abnormal liver function tests (Appendix 2) during the 12-week treatment period.

Fasting glucose levels will be measured by a glucometer and documented by study staff on Day 1 and prior to each dose. On non-visit days, subjects will also monitor and record their fasting glucose each morning and will contact the study site for a reading > 240 mg/dL or < 70 mg/dL. Subjects will be educated on symptoms and treatment of hypoglycemia and will obtain additional glucometer readings if they experience symptoms suggestive of hypoglycemia, or their glucose is < 70 mg/mL at any reading, as described in Appendix 3. Subjects will record any symptoms of hypoglycemia experienced at home and resulting treatment in a log, which will be reviewed by the Investigator at each visit commencing with Day 8.

Investigators will counsel subjects on how to keep their glucose levels within the limits, including repeated diet counseling, and will follow the decision criteria for the timing and method of intervention in subjects with persistent hyperglycemia during the 12-week treatment period (Appendix 4).

If a significant decrease of glucose (< 50 mg/dL) is repeatedly observed, or a subject requires interventions or external assistance to treat hypoglycemia, the subject may be dropped from the study.

The measures to be taken at each investigative site to minimize the risks of COVID-19 will be communicated to the subject, along with any changes to the risks of study participation that occur as a result of changing local COVID-19 conditions. Study participants will not be prevented from receiving approved or emergency-use authorized COVID-19 vaccines or treatments during their trial participation and will be advised accordingly. If an approved or

authorized COVID-19 vaccine has been administered in proximity to a scheduled IP administration, a ± 2-day window is allowed for IP dosing.

Stopping Rules:

Individual Subject Stopping Rules:

Subjects will be discontinued from receiving treatment for any of the following reasons:

- Any Grade 3 or 4 adverse event (AE) according to Common Terminology Criteria for Adverse Events (CTCAE) that is possibly or probably related to study drug

Subjects who prematurely discontinue study medication will remain in the study for early termination (ET) study assessments, with the date of visit adjusted to correspond to 25 days after the last dose of study medication, where the day of dosing is counted as the first day.

Study Stopping Rules:

The study will be paused, and causality assessed before resumption, if any one of the following three categories of AEs, as defined by CTCAE, occur:

- One fatal (Grade 5) event that is possibly or probably related to study drug
- Two Grade 4 events that are possibly or probably related to study drug
- Three Grade 3 events that are possibly or probably related to study drug

Eligibility Criteria:

The following Inclusion and Exclusion Criteria are applicable to the status of the subject on the screening visit for Study ALT-801-106 on Day -7 (coincides with the Day 78 visit of Study ALT-801-105) and must likewise be met on Day 1 prior to dosing for Study ALT-801-106 (coincides with the Day 85 visit of Study ALT-801-105), unless otherwise specified.

Inclusion Criteria:

1. Written informed consent to participate in this extension study, signed prior to the performance of any study procedures
2. Completion of the Day 85 visit procedures in Study ALT-801-105 with no more than 1 missed dose of IP
3. Female subjects of childbearing potential with negative urine pregnancy (beta human chorionic gonadotropin) test who are not breastfeeding, do not plan to become pregnant during the study, and agree to use highly effective birth control if they have sexual intercourse with a male partner (ie, oral contraceptives; contraceptive patches, implants, injections, and rings; intrauterine devices [IUD], both IUDs hormonally-impregnated and untreated; or sexual abstinence [only if this is in line with the subject's current lifestyle]) throughout the study and for at least 1 month after study completion;

   OR

   Female subjects of non-childbearing potential (ie, surgically [bilateral oophorectomy, hysterectomy, or tubal ligation] or naturally sterile [>12 consecutive months without menses]); or post-menopausal [>12 consecutive months without menses confirmed by follicular stimulating hormone (FSH) in the post-menopausal period];

   OR

Male subjects who have sexual intercourse with a female partner of child-bearing potential from the first dose of study drug until 1 month after study completion must either be surgically sterile (confirmed by documented azoospermia >90 days after the procedure) or have female partners that meet the requirements for female subjects. All male subjects must agree not to donate sperm from the first dose of study drug until 3 months after the last dose of study drug.

Subjects that have same-sex partners or practice sexual abstinence (only if this is in line with the subject's current lifestyle) are also permitted.

Exclusion Criteria:

1. Met any of the exclusion criteria in Study ALT-801-105 at the time of entry into that study
2. Body mass index (BMI) < 23 kg/mg^2
3. Development of any of the following conditions at any time during Study ALT-801-105:
   a. Type 1 diabetes mellitus and/or insulin-dependent Type 2 diabetes mellitus (T2DM), or uncontrolled T2DM requiring rescue therapy for hyperglycemia in Study ALT-801-105
   b. Acute pancreatitis, liver disorder, neoplasm (with the exception of non-melanomatous skin carcinoma or benign cervical neoplasia), acute significant gastrointestinal (GI) disorder (eg, peptic ulcers, severe gastroesophageal reflux disease [GERD]), inflammatory bowel disease, celiac disease or any medical condition that could affect gastric emptying, stool frequency or stool consistency. (Irritable bowel syndrome is permitted provided that bowel frequency and consistency are normal off treatment.)
   c. Undergone any gastrointestinal surgery
   d. Basal calcitonin level > 50 ng/L at the Week 7 visit of Study ALT-801-105
   e. Evidence of conduction abnormality including QT prolongation on electrocardiogram (ECG)
   f. Positive toxicology screening panel, including urine screen for amphetamines, barbiturates, benzodiazepines, cocaine (metabolite), methadone, morphine/opiates, phencyclidine and breath test for alcohol, or evidence of substance abuse or dependency or recreational intravenous drug use (by self-declaration) or excessive alcohol consumption defined as > 21 alcohol units per week for males and as >14 alcohol units per week for females (where 1 unit = half pint of beer, 25 mL of 40% spirit, or 125 mL of wine). Please note, a positive test that in the opinion of the Investigator and Sponsor can be attributed to a prescribed and appropriately used medication will not exclude a subject from study participation.
   g. Use of any of the following medications in the past 12 weeks:
      i. Insulin
      ii. Any glucagon-like peptide 1 (GLP-1) agonist or any other injectable diabetes treatment
      iii. Anti-obesity medications
      iv. Sulfonylureas, thiazolidinediones, or dipeptidyl peptidase-4 (DPP-4) inhibitors
      v. Herbal remedies (such as St. John's Wort)

h. Donated or received any blood or blood products during participation in Study ALT-801-105

i. Received another investigational product

j. Experienced a severe allergic or anaphylactic reaction, hypersensitivity reaction to GLP-1 or glucagon analogues or sensitivity or allergy to any components in the investigational product or a required procedure, including adhesives

k. Interim diagnosis of a clinically significant endocrine (eg, a new diagnosis of hypothyroidism [defined as thyroid-stimulating hormone (TSH) > 6 mIU/L]), neurological, GI, cardiovascular (except controlled hypertension and hypercholesterolemia), hematological, hepatic, immunological, renal, respiratory, or genitourinary abnormalities or diseases, or any medical, psychiatric, or social condition or occupational or other responsibility that in the judgment of the Investigator would interfere with or serve as a contraindication to protocol adherence, assessment of safety, or a subject's ability to give informed consent

4. Vital sign exclusions:

   a. Fever (body temperature >38.0°C)

   b. Poorly controlled hypertension as defined as:

      i. Systolic blood pressure > 150 mm Hg. and/or

      ii. Diastolic blood pressure ≥ 90 mm Hg

      For subjects not meeting these criteria on the Day -7 visit (Day 78 visit of ALT-801-105 study), blood pressure may be re-evaluated on the Day 1 visit (Day 85 visit of Study ALT-801-105) to establish eligibility at the discretion of the Investigator and Medical Monitor.

5. Clinically significant laboratory abnormalities within 2 weeks prior to the Day 78 of Study ALT-801-105 (2 weeks prior to the screening visit for Study ALT-801-106 on Day -7), including:

   a. Impaired renal function (estimated glomerular filtration rate [eGFR] < 60 mL/min/1.73 m^2) as estimated using the Modification of Diet in Renal Disease (MDRD) equation:

      GFR (mL/min/1.73 m^2) = 175 × (SCr/88.4)-1.154 × (Age)-0.203 × (0.742 if female) × (1.212 if African American) (SI units), where SCr is standardized serum creatinine in SI units (μmol/L) and age is in years

   b. Alanine aminotransferase (ALT) or aspartate aminotransferase (AST) laboratory values > 75 U/mL

   c. Alkaline phosphatase values > 2 × upper normal limits

   d. Total bilirubin > 1.3 mg/dL, with the exception of Gilbert Syndrome

   e. International normalized ratio (INR) > 1.3

   f. Hemoglobin < 11.0 g/dL

   g. Platelet count < 150,000/μL

   h. Any other abnormality deemed by the Investigator to exceed normal safety limits for this study or exclude subject participation.

Subjects not meeting any of these criteria may be retested at the discretion of the Medical Monitor

6. Subjects who, in the opinion of the Investigator, are unlikely to comply with the study protocol or would not be a suitable candidate for participation in the study

Endpoints:

Safety Endpoints:

- AEs
- Vital signs and Rate-Pressure Product (RPP calculated as mean heart rate × mean systolic blood pressure)
- Safety labs, including liver function tests and serum glucose
- Urinalysis
- Physical examination
- Immunogenicity (neutralizing antibodies)

Pharmacodynamic Endpoints:

Changes compared to baseline in

- Liver fat content
  - Hepatic fat fraction by MRI-PDFF
- Anthropometric parameters
  - Body weight
  - Waist circumference
  - Body composition by MRI scanning
- Lipid metabolism
  - Total cholesterol (TC)
  - Low density lipoprotein cholesterol (LDL-C)
  - High-density lipoprotein cholesterol (HDL-C)
  - Apolipoprotein A (Apo A) and B (Apo B)
  - Lipoprotein(a)
  - Triglycerides (TG)
- Metabolic markers
  - Hemoglobin A1c (HbA1c)
  - Adiponectin
  - Leptin
- Inflammatory markers
  - Tumor necrosis factor (TNF)

  - High-sensitivity C-reactive protein (hs-CRP)
  - Monocyte chemoattractant protein-1 (MCP-1)
  - Interleukin-6 (IL-6)
  - Plasminogen activator inhibitor-1 (PAI-1)
- Fibrosis markers
  - N-terminal type III collagen propeptide (Pro-C3)
  - Enhanced Liver Fibrosis (ELF) test
  - Fibroscan
  - Corrected T1 (cT1) magnetic resonance imaging (MRI)
- Lipotoxicity markers
  - Specific lipids to be analyzed will be described in a separate analysis plan

Quality of Life Endpoint:

- Changes in Short Form-36 (SF-36) and Impact of Weight on Quality of Life-Lite Clinical Trials version (IWQoL-Lite for CT) compared to baseline

Statistical Methods:

Power and Sample Size Assumptions:

This is an extension study that is being offered to participants of Study ALT-801-105, and no sample size calculation is applicable.

Statistical Analysis:

General:

All subjects who receive at least 1 dose of study medication (Safety Population) will be included in the safety analyses. The assessments of the secondary and PD endpoints will be conducted in the PD population, which consists of all subjects who receive at least 1 dose of study medication and who have results from at least 1 post-baseline PD assessment.

Descriptive statistics, including the numbers and percentages for categorical variables and the numbers, means, standard deviations, medians, minimums and maximums for continuous variables will be provided by dose and treatment, and by day when applicable.

Baseline for this study is defined as data collected on the Day 85 visit of Study ALT-801-105 and prior to the dosing on Day 1 of this study. Two sets of baseline comparisons will be conducted: 1) comparisons to the baseline of Study ALT-801-105; and 2) comparisons to the baseline for this study.

Interim Analysis:

An interim analysis will be conducted after all subjects complete 12 weeks of treatment. The conduct of the interim analysis will be detailed in the SAP.

Safety and Tolerability:

A medical occurrence will be reported as an AE if it represents 1) a new occurrence since the completion of Study ALT-801-105, 2) worsening in the severity of a previously reported AE, or 3) the development of seriousness criteria in a previously reported AE.

Continuous safety data will be summarized with descriptive statistics (arithmetic mean, standard deviation [SD], median, minimum, and maximum) by dose level. Categorical safety data will be summarized with frequency counts and percentages by dose level and day where applicable.

AEs will be coded using the most current Medical Dictionary for Regulatory Activities (MedDRA) version. A by-subject AE data listing, including verbatim term, preferred term, system organ class (SOC), treatment, severity, and relationship to study medication, will be provided. The number of subjects experiencing treatment-emergent AEs (TEAEs) and number of individual TEAEs will be summarized by treatment group, SOC and preferred term. TEAEs will also be summarized by severity and by relationship to study medication.

Laboratory evaluations, including liver function tests and FPG, vital signs (including calculation of RPP), and ECG assessments will be summarized by treatment group, dose levels, and protocol specified collection time point. A summary of change from baseline at each protocol specified time point by treatment group will also be presented.

Changes in physical examinations will be listed for each subject.

Concomitant medications will be listed by subject and coded using the most current version of the World Health Organization (WHO) Drug Dictionary.

Pharmacodynamics:

Descriptive statistics, including the numbers and percentages for categorical variables and the numbers, means, SDs, medians, minimums, and maximums for continuous variables will be provided by dose level and by day when applicable.

Changes from baselines in MRI-PDFF, anthropometric parameters and body composition, lipid metabolism, metabolic markers, fibrosis markers, lipotoxicity markers, inflammation markers, FibroScan, and cT1 (for subjects in whom cT1 was performed) will be listed and summarized by treatment group and strata with descriptive statistics (sample size [N], arithmetic mean, SD, median, minimum, maximum, geometric mean, and geometric coefficient of variation [CV%]). The effects of baseline BMI on PD parameters will be evaluated by covariate analyses.

Inferential statistics will be performed, as applicable. All analyses will be described in a statistical analysis plan (SAP). The changes in weight and other continuous variables will be compared between ALT-801 and placebo groups using the analysis of covariance (ANCOVA) test, where treatment arm as a factor and the stratification of presence or absence of diabetes or the corresponding baseline demographic characteristics (gender, race, BMI) as covariates. The Cochrane Mantel Haenszel test will be applied for secondary endpoints that are categorical in nature, while considering the stratification of presence or absence of diabetes, at a one-sided significance level of 0.025.

Quality of Life:

Changes from baseline in the 2 summary scores for physical health and mental health, and 8 domain scores for SF-36 and the composite score for the IWQoL-Lite for CT will be listed and summarized by treatment group with descriptive statistics (N, arithmetic mean, SD, median, minimum, maximum, geometric mean, and geometric CV%). Inferential statistics applicable to continuous endpoints will be applied, as described above.

TABLE OF CONTENTS

CLINICAL PROTOCOL APPROVAL FORM 2
CONFIDENTIALITY AND INVESTIGATOR STATEMENT 3
STUDY SUMMARY 4
LIST OF ABBREVIATIONS 17
1. INTRODUCTION AND RATIONALE 19
1.1. Background 19
1.2. Nonclinical Studies 19
1.3. Clinical Studies 19
1.4. Study Rationale 20
2. STUDY OBJECTIVES 21
2.1. Safety Objective 21
2.2. Pharmacodynamic Objectives 21
2.3. Quality of Life Objective 21
3. STUDY ENDPOINTS 22
3.1. Safety Endpoints 22
3.2. Pharmacodynamic Endpoints 22
3.3. Quality of Life Endpoint 23
4. STUDY PLAN 24
4.1. Study Design 24
4.2. Rationale for Study Design 25
4.3. Rationale for Study Population 25
4.4. Dose Rationale 25
4.5. Schedule of Assessments 25
5. POPULATION 29
5.1. Number of Subjects 29
5.2. Inclusion Criteria 29
5.3. Exclusion Criteria 30
6. STUDY MEDICATION 32
6.1. Description 32
6.1.1. Formulation 32
6.1.1.1. ALT-801 32

6.1.1.2. Placebo 32
6.1.2. Packaging, Storage, and Handling 32
6.2. Randomization 32
6.3. Dose and Administration 32
6.4. Dosing Modifications 33
6.5. Blinding and Unblinding 33
6.6. Accountability 33
6.7. Prior and Concomitant Therapy 34
6.8. Contraception 35
6.9. Compliance 35
7. PREMATURE DISCONTINUATION 36
7.1. Individual Subjects 36
7.2. Stopping Rules 36
7.2.1. Individual Subject Stopping Rules 36
7.2.2. Study Stopping Rules 36
7.3. Study Termination 36
8. DESCRIPTION OF STUDY PROCEDURES 38
8.1. Pharmacodynamic Assessments 38
8.1.1. Weight, Waist Circumference, and Body Mass Index (BMI) 38
8.1.2. Lipids 38
8.1.3. Metabolic Markers 38
8.1.4. Inflammatory Markers 38
8.1.5. Fibrosis Markers 38
8.1.6. Lipotoxicity Markers 39
8.1.7. Stored Samples for Future Analysis 39
8.1.8. Imaging 39
8.2. Quality of Life Assessments 39
8.3. Immunogenicity Assessment 39
8.4. Dietary and Exercise Counseling 39
8.5. Safety Assessments 40
8.5.1. Adverse Events 40
8.5.2. Safety Laboratory Tests 40

8.5.3. Glucose Monitoring 40
8.5.4. Pregnancy Tests 40
8.5.5. Physical Examination 40
8.5.6. Vital Signs 41
8.5.7. Electrocardiograms 41
9. ADVERSE EVENTS 42
9.1. Definitions 42
9.1.1. Adverse Event 42
9.1.2. Adverse Drug Reaction 42
9.1.3. Unexpected Adverse Drug Reaction 42
9.1.4. Serious Adverse Event 43
9.2. Reporting Responsibilities and Periods 43
9.3. Assessment of Adverse Events 44
9.3.1. Severity 44
9.3.2. Relatedness (Causality) 44
9.4. Safety Laboratory, Physical Examination, Electrocardiogram, and Vital Sign Abnormalities 45
9.5. Pregnancy 45
9.6. Overdose 45
9.7. Procedures for Recording and Reporting Adverse Events 46
9.7.1. Recording Adverse Events 46
9.7.2. Reporting of Serious Adverse Events or Adverse Events Meeting Criteria for Individual Subject Stopping Rules 46
9.7.3. Special Reporting Situations 47
9.7.4. Reporting Pregnancies 47
9.8. Safety Assessment Committee 47
9.9. Medical Monitor 48
10. STATISTICS 49
10.1. General Procedures 49
10.2. Power and Sample Size Assumptions 49
10.3. Analysis Sets 49
10.4. Statistical Methods 49

10.4.1. Safety/Tolerability 49
10.4.2. Pharmacodynamics 50
10.4.3. Quality of Life 50
10.5. Statistical Analysis Plan 50
11. DATA QUALITY ASSURANCE....51
11.1. Data Handling 51
11.2. Computer Systems 51
11.3. Data Entry 51
11.4. Medical Information Coding 51
11.5. Data Validation 51
11.6. Study Monitoring Requirements....51
11.7. Source Document and Case Report Form Completion....52
11.8. Record Retention 52
12. ETHICS 54
12.1. Good Clinical Practice 54
12.2. Institutional Review Board / Independent Ethics Committee 54
12.3. Subject Information and Consent 54
12.4. Subject Confidentiality 54
12.5. Protocol Compliance 55
13. COMPENSATION, INSURANCE, AND INDEMNITY 56
14. PUBLICATION POLICY 57
15. CHANGES TO PROTOCOL 58
16. REFERENCES 60
17. APPENDICES 61
APPENDIX 1. SAFETY LABORATORY TESTS 62
APPENDIX 2. ALGORITHM FOR MONITORING ABNORMAL LIVER FUNCTION TESTS....63
APPENDIX 3. INVESTIGATOR-PROVIDED INSTRUCTIONS TO SUBJECT: IDENTIFICATION AND MANAGEMENT OF HYPOGLYCEMIA 65
APPENDIX 4. RESCUE THERAPY FOR PERSISTENT HYPERGLYCEMIA 66

LIST OF TABLES

Table 1 Schedule of Assessments 26

LIST OF ABBREVIATIONS

Abbreviation	Definition
AE	adverse event
ALP	alkaline phosphatase
ALT	alanine aminotransferase
Apo A, B	apolipoprotein A, B
AST	aspartate aminotransferase
BMI	body mass index
CRA	Clinical Research Associate
CTCAE	Common Terminology Criteria for Adverse Events
CV%	coefficient of variation
DDI	drug-drug interaction
DPP-4	dipeptidyl peptidase-4 inhibitor
ECG	electrocardiogram
eCRF	electronic case report form
eGFR	estimated glomerular filtration rate
ELF	Enhanced Liver Fibrosis test
GCP	Good Clinical Practice
GGT	gamma glutamyl transferase
GI	gastrointestinal
GLP-1	glucagon-like peptide 1
HDL-C	high-density lipoprotein cholesterol
hs-CRP	high sensitivity C-reactive protein
ICH	International Council for Harmonisation
IEC	Independent Ethics Committee
IL-6	interleukin-6
INR	international normalized ratio
IRB	Institutional Review Board
IUD	intrauterine device
IWQoL-Lite for CT	Impact of Weight on Quality of Life-Lite Clinical Trials version
LDL-C	low-density lipoprotein cholesterol
MAD	multiple ascending dose
MCP-1	monocyte chemoattractant protein-1

Abbreviation	Definition
MedDRA	Medical Dictionary for Regulatory Activities
NAFLD	non-alcoholic fatty liver disease
NASH	non-alcoholic steatohepatitis
PAI-1	plasminogen activator inhibitor-1
PD	pharmacodynamic(s)
Pro-C3	N-terminal type III collagen propeptide
PV	Pharmacovigilance
QoL	quality of life
RPP	Rate-Pressure Product
SAD	single ascending dose
SAE	serious adverse event
SAP	statistical analysis plan
SC	subcutaneous
SD	standard deviation
SMP	Safety Management Plan
SOC	system organ class
SAC	Safety Assessment Committee
SF-36	Short Form-36
T2DM	Type 2 diabetes mellitus
TEAE	treatment-emergent adverse event
TG	triglycerides
TNF	tumor necrosis factor
ULN	upper limit of normal
WHO	World Health Organization

1. INTRODUCTION AND RATIONALE

1.1. Background

ALT-801 is a modified 29-amino acid peptide with equipotent dual agonist properties for the glucagon-like peptide 1 receptor (GLP-1R) and glucagon receptor (GCR). ALT-801 is being developed for non-alcoholic steatohepatitis (NASH), a subgroup of non-alcoholic fatty liver disease (NAFLD) where steatosis leads to hepatocyte injury and inflammation (steatohepatitis), with or without fibrosis, and for weight loss.

1.2. Nonclinical Studies

In Good Laboratory Practice (GLP) toxicity studies of up to 13-weeks in duration in rats and monkeys, there were no adverse findings relating ALT-801 to mortality, clinical observations, ophthalmic observations, clinical pathology, organ weights, macroscopic, or microscopic examinations during the dosing or recovery period in either species. Further, in monkeys, no ALT-801-related body condition effects, electrocardiogram (ECG) findings, neurological findings, respiration rates effects, or dermal observations were noted during the course of this study. Observations were limited to those associated with decreased food consumption and body weight loss, which was greater in rats than in monkeys.

Reduced food consumption and weight loss observations were expected on-target effects of glucagon-like peptide 1 (GLP-1) and glucagon agonism. These effects were more pronounced in rats compared to monkeys, possibly related to the more frequent dose cycle (once daily initially) corresponding to the shorter half-life in rats.

Chronic GLP toxicology (26-week/rat and 39-week/cynomolgus monkey studies) to support chronic administration revealed a similar pattern of findings as previous studies. There were no new or unexpected toxicities and NOAELs were based on ALT-801 effects on BW loss and related observations, which are considered on target effects that are desirable, monitorable, and reversible.

No significant cytochrome (either induction or inhibition) or transporter interactions have noted in *in vitro* drug-drug interaction (DDI) studies that have been conducted to date.

Please refer to the Investigator's Brochure for detailed information on the nonclinical program.

1.3. Clinical Studies

Altimmune is completing a Phase 1, first-in-human study (ALT-801-101) in Australia under a clinical trial application. The study consisted of approximately 70 subjects enrolled across single ascending dose (SAD) cohorts of ALT-801 0.4 mg, 1.2 mg, 2.4 mg, 3.6 mg, and 4.8 mg and multiple ascending dose (MAD) cohorts of ALT-801 1.2 mg, 1.8 mg and 2.4 mg administered subcutaneously (SC) at weekly intervals for 12 weeks in a double-blind manner without dose titration. Two additional cohorts (15 subjects per cohort) were added to explore whether use of dose titration might facilitate improved tolerability such that higher doses could be evaluated in subsequent trials. Thus, a total of approximately 100 volunteers are participating in the trial across the 5 SAD and 5 MAD cohorts.

An interim analysis comprised of data from the aforementioned 5 SAD cohorts and each of the 3 MAD cohorts through 12 weeks of weekly dosing in Study ALT-801-101 has been conducted. With regard to safety, single doses of ALT-801 up to 3.6 mg SC in the SAD phase

were deemed tolerated, with a significant incidence in nausea and vomiting (recognized dose-related sequelae of GLP-1 agents) at the 4.8 mg SC dose. Twelve weekly doses of 1.2 mg SC, 1.8 mg SC, and 2.4 mg SC (MAD cohorts 1-3) were deemed tolerated, even without the use of dose titration, with low to moderate rates of nausea, vomiting, diarrhea and constipation, all recognized side-effects of GLP-1 therapies. No grade 3 adverse events (AEs) were identified; no discontinuations due to AEs or serious AEs (SAEs) were reported. Glycemic control appeared unaffected, with no significant changes in fasting blood sugar or hemoglobin A1C and no hyperglycemia AEs observed through 12 weeks of dosing. Sporadic hypoglycemia AEs were reported across ALT-801 treatment arms, with all events resolving rapidly with eating. While hypoglycemic events are common with GLP-1 class agents, there was no evidence that these events were exacerbated in either severity or duration by concomitant glucagon agonism.

Transient elevations in liver function tests in the range of 3-5 × baseline were detected in 2 subjects: one subject who received ALT-801 1.8 mg SC weekly and one subject who received placebo. Both subjects had alanine aminotransferase (ALT) elevations at baseline, as permitted by protocol inclusion and exclusion criteria, which allowed up to 2 × the upper limit of normal (ULN) for study eligibility. ALT elevations resolved during treatment. Aspartate aminotransferase (AST), alkaline phosphatase (ALP), and gamma glutamyl transferase (GGT) were 2 × or less the respective ULNs, and no elevations in bilirubin or international normalized ratio (INR) were observed. ALT abnormalities were not dose related and were not observed in subjects who received ALT-801 at doses of 2.4 mg or higher weekly for 12 weeks. No aggregate or time-related trends in liver tests (ALT, AST, ALP, GGT, bilirubin) were observed at any dose.

Mean weight losses at 12 weeks were 4.9%, 10.3% and 9.0% in 1.2 mg SC, 1.8 mg SC and 2.4 mg SC weekly MAD treatment groups, respectively.

Substantial decreases in systolic and diastolic blood pressure were noted in the absence of significant increases in heart rate. These were accompanied by significant decreases in total cholesterol, LDL cholesterol, and triglycerides over 12 weeks treatment.

Pharmacokinetic modeling indicated a $t_{1/2}$ of approximately 103 to 135 hrs, resulting in an accumulation factor of approximately 1.2 to 1.8 with weekly dosing and steady state concentrations by Dose 3, and time to maximal concentration of approximately 54 to 78 hours following a single dose, with median T_{max} between 54 to 66 hours. No subjects exceeded steady state exposures at the NOAEL in toxicity studies. Overall, these data supported the dose schedule of once weekly administration.

For additional information, please see the ALT-801 Investigators Brochure.

1.4. Study Rationale

This extension study is designed to allow for an additional 12 weeks of treatment with investigational product (IP; ALT-801 or placebo) for subjects who complete Study ALT-801-105 in order to assess the safety and effects of 24 weeks of treatment with ALT-801 compared to placebo on anthropometric parameters, lipid metabolism, and inflammatory, metabolic, fibrosis, and lipotoxicity markers in diabetic and non-diabetic overweight and obese subjects with NAFLD. Thus, the screening visit for Study ALT-801-106 on Day -7 coincides with receipt of the last dose of IP on Day 78 of Study ALT-801-105 and Day 1 for Study ALT-801-106 occurs the following week, coinciding with the Day 85 visit of Study ALT-801-105. This overlap enables uninterrupted dosing for 24 weeks upon completion of Study ALT-801-106.

2. STUDY OBJECTIVES

2.1. Safety Objective

To assess the safety and tolerability of ALT-801 in subjects with NAFLD.

2.2. Pharmacodynamic Objectives

To evaluate the effects of ALT-801 on:

- Liver fat content
- Anthropometric parameters, including body composition
- Lipid metabolism
- Metabolic markers
- Inflammatory markers
- Fibrosis markers
- Lipotoxicity markers

2.3. Quality of Life Objective

To evaluate the effects of ALT-801 on quality of life (QoL) using established QoL instruments.

3. STUDY ENDPOINTS

3.1. Safety Endpoints

- AEs
- Vital signs and Rate-Pressure Product (RPP calculated as mean heart rate × mean systolic blood pressure)
- Safety labs, including liver function tests and serum glucose
- Urinalysis
- Physical examination
- Immunogenicity (neutralizing antibodies)

3.2. Pharmacodynamic Endpoints

- Changes compared to baseline in
  - Liver fat content
    - Hepatic fat fraction by MRI-PDFF
  - Anthropometric parameters
    - Body weight
    - Waist circumference
    - Body composition by MRI scanning
  - Lipid metabolism
    - Total cholesterol (TC)
    - Low density lipoprotein cholesterol (LDL-C)
    - High-density lipoprotein cholesterol (HDL-C)
    - Apolipoprotein A (Apo A) and B (Apo B)
    - Lipoprotein(a)
    - Triglycerides (TG)
  - Metabolic markers
    - Hemoglobin A1c (HbA1c)
    - Adiponectin
    - Leptin
  - Inflammatory markers
    - Tumor necrosis factor (TNF)
    - High-sensitivity C-reactive protein (hs-CRP)

    - Monocyte chemoattractant protein-1 (MCP-1)
    - Interleukin-6 (IL-6)
    - Plasminogen activator inhibitor-1 (PAI-1)
  - Fibrosis markers
    - N-terminal type III collagen propeptide (Pro-C3)
    - Enhanced Liver Fibrosis (ELF) test
    - Fibroscan
    - Corrected T1 (cT1) magnetic resonance imaging (MRI)
  - Lipotoxicity markers
    - Specific lipids to be analyzed will be described in a separate analysis plan

3.3. Quality of Life Endpoint

- Changes in Short Form-36 (SF-36) and Impact of Weight on Quality of Life-Lite Clinical Trials version (IWQoL-Lite for CT) compared to baseline

4. STUDY PLAN

4.1. Study Design

This is a 12-week extension study to assess the safety and effects of a total of 24 weeks of treatment with ALT-801 on liver fat content, anthropometric parameters and body composition, lipid metabolism, and inflammatory, metabolic, fibrosis, and lipotoxicity markers in diabetic and non-diabetic overweight and obese subjects with NAFLD. Subjects who completed the Day 85 visit with no more than 1 missed dose of IP in Study ALT-801-105 and who continue to meet eligibility criteria will receive 12 additional weeks of the same treatment they received in Study ALT-801-105. Treatment will remain blinded, and neither the investigator nor subject will be aware of the treatment being received.

Informed consent will be obtained at the screening visit for Study ALT-801-106 on Day -7 (coincides with the Day 78 visit of Study ALT-801-105) and the first dose of IP will be administered on Day 1 (coincides with the Day 85 visit of Study ALT-801-105), as indicated in Table 1. Subsequent visits will be conducted at the clinic, home, or work through the Day 85 or early termination visit of this study. Subjects will return for a safety follow-up visit on Day 110.

Counseling on diet and exercise, as provided in Study ALT-801-105, will continue to be provided, as indicated in Table 1.

Investigators will follow the decision criteria for the timing and method of intervention in subjects who develop worsening abnormal liver function tests (Appendix 2) during the 12-week treatment period.

Fasting glucose levels will be measured by a glucometer and documented by study staff on Day 1 and prior to each dose. On non-visit days, subjects will also monitor and record their fasting glucose each morning and will contact the study site for a reading > 240 mg/dL or < 70 mg/dL. Subjects will be educated on symptoms and treatment of hypoglycemia and will obtain additional glucometer readings if they experience symptoms suggestive of hypoglycemia, or their glucose is < 70 mg/mL at any reading, as described in Appendix 3. Subjects will record any symptoms of hypoglycemia experienced at home and resulting treatment in a log, which will be reviewed by the Investigator at each visit commencing with Day 8.

Investigators will counsel subjects on how to keep their glucose levels within the limits, including repeated diet counseling, and will follow the decision criteria for the timing and method of intervention in subjects with persistent hyperglycemia during the 12-week treatment period (Appendix 4).

If a significant decrease of glucose (< 50 mg/dL) is repeatedly observed, or a subject requires interventions or external assistance to treat hypoglycemia, the subject may be dropped from the study.

The measures to be taken at each investigative site to minimize the risks of COVID-19 will be communicated to the subject, along with any changes to the risks of study participation that occur as a result of changing local COVID-19 conditions. Study participants will not be prevented from receiving approved or emergency-use authorized COVID-19 vaccines or treatments during their trial participation and will be advised accordingly. If an approved or

authorized COVID-19 vaccine has been administered in proximity to a scheduled IP administration, a ± 2-day window is allowed for IP dosing.

4.2. Rationale for Study Design

The continued effects of ALT-801 on safety and tolerability in subjects with NAFLD will be monitored in this extension study, as noted in Section 4.1. As in Study ALT-801-105, the study will intensively monitor the effects of ALT-801 on glucose, both hyperglycemia and hypoglycemia, and liver function tests will be monitored on a frequent basis. Investigators will be directed to follow the decision criteria for the timing and method of intervention in subjects who develop worsening abnormal liver function tests, if observed (Appendix 2), in response to the sporadic elevations of ALT and AST observed in Study ALT-801-101. The proposed plan will identify and mitigate risk of liver injury and glucose excursions in study participants.

The study will also assess changes in liver fat content, anthropometric parameters and body composition, lipid metabolism, and metabolic, inflammatory, fibrosis and lipotoxicity markers after 24 weeks of ALT-801 treatment compared to placebo.

4.3. Rationale for Study Population

Subjects with body mass index BMI ≥ 23 kg/m^2 at the end of Study ALT-801-105 are allowed to participate in this extension study because these subjects will be able to better tolerate the additional weight loss predicted with continued treatment. Subjects that require insulin, sulfonylureas or DDP-4 inhibitors for control of diabetes have been excluded in this initial assessment in diabetics to provide for a more uniform subject population and minimize the potential for hypoglycemia in early phase clinical trials. Subjects will also be excluded if they meet the criteria for rescue therapy for hyperglycemia in Study ALT-801-105. Exclusions have been instituted that might otherwise affect an accurate assessment of the effects of ALT-801 on safety or pharmacodynamics (PD).

4.4. Dose Rationale

ALT-801 doses in this extension study are the same as those used during the original 12-week treatment period in Study ALT-801-105 and will enable accumulation of safety and pharmacodynamic data over 24 weeks treatment.

4.5. Schedule of Assessments

The schedule for study activities is presented in Table 1.

Table 1 Schedule of Assessments

**Period**	**Screening**	**Treatment Period**													**Follow-up**
**Week**	**(12)[a]**	**(13)/1**	**2**	**3**	**4**	**5**	**6**	**7**	**8**	**9**	**10**	**11**	**12**	**13/ET**	**18**
**Day**	**(78 ±2)[a]/ -7**	**(85 ± 2)[a]/ 1**	**8 ±2**	**15 ±2**	**22 ±2**	**29 ±2**	**36 ±2**	**43 ±2**	**50 ±2**	**57 ±2**	**64 ±2**	**71 ±2**	**78 ±2**	**85 ±2**	**110 ±5**
**Study Drug Administration [b]**	*(X)*[c]	**X**	**X**	**X**	**X**	**X**	**X**	**X**	**X**	**X**	**X**	**X**	**X**		
Eligibility Assessments															
Informed Consent	X														
Inclusion/Exclusion Criteria	X	X													
General Procedures															
Waist Circumference		*X*						X						X	
Body Weight	*X*	*X*	X	X	X	X	X	X	X	X	X	X	X	X	X
Body Mass Index	*X*	*X*	X	X	X	X	X	X	X	X	X	X	X	X	X
Counseling on diet and daily activities [d]	*X*	*X*	X	X	X	X	X	X	X	X	X	X	X	X	
Safety Assessments															
Complete Physical Exam	X														X
Abbreviated Physical Exam	*X*	*X*	X	X	X	X	X	X	X	X	X	X	X	X	
Vitals	*X*	*X*	X	X	X	X	X	X	X	X	X	X	X	X	X
12-Lead ECG [e]	X	*X*						X						X	X
Hypoglycemia log [f]	*X*	*X*	←————————————————————→												
Review Concomitant Medications	*X*	*X*	X	X	X	X	X	X	X	X	X	X	X	X	X
Adverse Events [g]	*X*	*X*	X	X	X	X	X	X	X	X	X	X	X	X	X
Urine human chorionic gonadotropin (females of childbearing potential only)	X	*X*			X			X			X			X	X
Urinalysis [h]	X	*X*						X						X	X
Chemistry [h, i]	X	*X*			X			X			X			X	X
Hematology and coagulation [h]	X	*X*			X			X			X			X	X

**Period**	**Screening**	**Treatment Period**													**Follow-up**
**Week**	**(12)[a]**	**(13)/1**	**2**	**3**	**4**	**5**	**6**	**7**	**8**	**9**	**10**	**11**	**12**	**13/ET**	**18**
**Day**	**(78 ±2)[a]/ -7**	**(85 ± 2)[a]/ 1**	**8 ±2**	**15 ±2**	**22 ±2**	**29 ±2**	**36 ±2**	**43 ±2**	**50 ±2**	**57 ±2**	**64 ±2**	**71 ±2**	**78 ±2**	**85 ±2**	**110 ±5**
**Study Drug Administration [b]**	*(X)[c]*	X	X	X	X	X	X	X	X	X	X	X	X		
Glucometer Glucose Test [i]	*X*	*X*	X	X	X	X	X	X	X	X	X	X	X	X	
Serum samples for biorepository		*X*						X						X	
Specialty Assessments															
Urine Drug Screen	*X*	*X*						X						X	
Alcohol Breath Test	*X*	*X*						X						X	
Calcitonin		*X*						X						X	
FibroScan [i, j]		*X*												X	
MRI-PDFF and cT1 [i, j, k]		*X*												X	
Body Composition by MRI [i, j]		*X*												X	
Lipid Metabolism [i, j]		*X*												X	X
Metabolic Markers [i, j]		*X*												X	
Inflammatory Markers [i, j]		*X*												X	
Fibrosis Markers [i, j]		*X*												X	
Lipotoxicity Markers [i, j]		*X*												X	
PK for ALT-801	*X*	*X*													
PK for metformin [n]	*X*														
Immunogenicity [l]		X													X
SF-36 and IWQoL-Lite [m]		*X*												X	

Abbreviations: ECG = electrocardiogram; ET = early termination; HbA1c = hemoglobin A1c; IWQoL-Lite = Impact of Weight on Quality of Life-Lite; SF-36 Short Form-36
All assessments should be performed predose, unless specified.

[a] Time point in parentheses = time point or visit from Study ALT-801-105. X 's in these columns represent the procedures in Study ALT-801-105, supplemented by the procedures specific to this extension study. Grayed out italicized X's represent procedures conducted and recorded for ALT-801-105.

[b] Study visits that do not require laboratory draws may be conducted at the subject's home/office.

[c] Study drug administered in Study ALT-801-105

[d] Counseling provided in Study ALT-801-105 will continue. The subject's routine diet and daily activities will be established at that time, and the subject will be counseled to maintain these lifestyles over the next 12 weeks of treatment. Counseling will be reinforced at each subsequent visit.

[e] Subjects will be required to be semi-recumbent and abstain from external stimulus for at least 5 minutes during the ECG preparation and collection period. When multiple activities occur at the same timepoint, ECGs should be collected first, followed by other assessments and blood draws. Any clinically relevant changes in ECG findings will be recorded as AEs.

[f] Subjects will record any symptoms of hypoglycemia experienced at home in a log, which will be reviewed by the Investigator at each visit.

[g] All AEs prior to Dose 1 will be collected though Day 85 in Study ALT-801-105; all AE's after Dose 1 will be collected on this study. AEs prior to dose 1 that continue after dose 1 will be listed as ongoing.

[h] See Appendix 1.

[i] To be performed after a minimum 8 hr fast.

[j] See Sections 8.1.2, 8.1.3, 8.1.4, 8.1.5, 8.1.6, 8.1.7, and 8.1.8.

[k] cT1 MRI will only be performed for subjects who had cT1 performed in Study ALT-801-105

[l] If an AE requires follow-up beyond the follow-up visit at the end of the study, additional samples monthly or at resolution, if sooner, will be collected to assess the potential relationship to anti-drug antibodies

[m] See Section 8.2

[n] Metformin PK sample collection within 1 h before ALT-801 administration. The subject or clinical unit reported time, condition (fed or fasted) and dose level of last metformin dose must be recorded.

5. POPULATION

5.1. Number of Subjects

Up to approximately 90 overweight and obese diabetic and nondiabetic subjects with nonalcoholic fatty liver disease (NAFLD) who completed the Day 85 visit of Study ALT-801-105 are planned for enrollment in this study.

5.2. Inclusion Criteria

The following Inclusion and Exclusion Criteria are applicable to the status of the subject on the screening visit of Study ALT-801-106 on Day -7 (coincides with the Day 78 visit of Study ALT-801-105) and must likewise be met on Day 1 prior to dosing for Study ALT-801-106 (coincides with the Day 85 visit of Study ALT-801-105), unless otherwise specified.

1. Written informed consent to participate in this extension study, signed prior to the performance of any study procedures
2. Completion of the Day 85 visit procedures in Study ALT-801-105, with no more than 1 missed dose of IP
3. Female subjects of childbearing potential with negative urine pregnancy (beta human chorionic gonadotropin) test who are not breastfeeding, do not plan to become pregnant during the study, and agree to use highly effective birth control if they have sexual intercourse with a male partner (ie, oral contraceptives; contraceptive patches, implants, injections, and rings; intrauterine devices [IUD], both IUDs hormonally-impregnated and untreated; or sexual abstinence [only if this is in line with the subject's current lifestyle]) throughout the study and for at least 1 month after study completion;

   OR

   Female subjects of non-childbearing potential (ie, surgically [bilateral oophorectomy, hysterectomy, or tubal ligation] or naturally sterile [>12 consecutive months without menses]); or post-menopausal [>12 consecutive months without menses confirmed by follicular stimulating hormone (FSH) in the post-menopausal period];

   OR

   Male subjects who have sexual intercourse with a female partner of child-bearing potential from the first dose of study drug until 1 month after study completion must either be surgically sterile (confirmed by documented azoospermia >90 days after the procedure) or have female partners that meet the requirements for female subjects. All male subjects must agree not to donate sperm from the first dose of study drug until 3 months after the last dose of study drug.

   Subjects that have same-sex partners or practice sexual abstinence (only if this is in line with the subject's current lifestyle) are also permitted.

5.3. Exclusion Criteria

Subjects must NOT meet any of the following exclusion criteria:

1. Met any of the exclusion criteria in Study ALT-801-105 at the time of entry into that study
2. Body mass index (BMI) < 23 kg/m^2
3. Development of any of the following conditions at any time during Study ALT-801-105:
    a. Type 1 diabetes mellitus and/or insulin-dependent Type 2 diabetes mellitus (T2DM), or uncontrolled T2DM requiring rescue therapy for hyperglycemia in Study ALT-801-105.
    b. Acute pancreatitis, liver disorder, neoplasm (with the exception of non-melanomatous skin carcinoma or benign cervical neoplasia), acute significant gastrointestinal (GI) disorder (eg, peptic ulcers, severe gastroesophageal reflux disease [GERD]), inflammatory bowel disease, celiac disease or any medical condition that could affect gastric emptying, stool frequency or stool consistency. (Irritable bowel syndrome is permitted provided that bowel frequency and consistency are normal off treatment.)
    c. Undergone any gastrointestinal surgery
    d. Basal calcitonin level > 50 ng/L at the Week 7 visit of Study ALT-801-105
    e. Evidence of conduction abnormality including QT prolongation on electrocardiogram (ECG)
    f. Positive toxicology screening panel, including urine screen for amphetamines, barbiturates, benzodiazepines, cocaine (metabolite), methadone, morphine/opiates, phencyclidine and breath test for alcohol, or evidence of substance abuse or dependency or recreational intravenous drug use (by self-declaration) or excessive alcohol consumption defined as > 21 alcohol units per week for males and as >14 alcohol units per week for females (where 1 unit = half pint of beer, 25 mL of 40% spirit, or 125 mL of wine). Please note, a positive test that in the opinion of the Investigator and Sponsor can be attributed to a prescribed and appropriately used medication will not exclude a subject from study participation.
    g. Use of any of the following medications in the past 12 weeks:
        i. Insulin
        ii. Any glucagon-like peptide 1 (GLP-1) agonist or any other injectable diabetes treatment
        iii. Anti-obesity medications
        iv. Sulfonylureas, thiazolidinediones, or dipeptidyl peptidase-4 (DPP-4) inhibitors
        v. Herbal remedies (such as St. John's Wort)
    h. Donated or received any blood or blood products during participation in Study ALT-801-105
    i. Received another investigational product
    j. Experienced a severe allergic or anaphylactic reaction, hypersensitivity reaction to GLP-1 or glucagon analogues or sensitivity or allergy to any components in the investigational product or a required procedure, including adhesives

k. Interim diagnosis of a clinically significant endocrine (eg, a new diagnosis of hypothyroidism [defined as thyroid-stimulating hormone (TSH) > 6 mIU/L]), neurological, GI, cardiovascular (except controlled hypertension and hypercholesterolemia), hematological, hepatic, immunological, renal, respiratory, or genitourinary abnormalities or diseases, or any medical, psychiatric, or social condition or occupational or other responsibility that in the judgment of the Investigator would interfere with or serve as a contraindication to protocol adherence, assessment of safety, or a subject's ability to give informed consent

4. Vital sign exclusions:
   a. Fever (body temperature >38.0°C)
   b. Poorly controlled hypertension as defined as:
      i. Systolic blood pressure > 150 mm Hg. and/or
      ii. Diastolic blood pressure ≥ 90 mm Hg

   For subjects not meeting these criteria on the Day -7 visit (Day 78 visit of Study ALT-801-105), blood pressure may be re-evaluated on the Day 1 visit (Day 85 visit of Study ALT-801-105) to establish eligibility at the discretion of the Investigator and Medical Monitor.

5. Clinically significant laboratory abnormalities within 2 weeks prior to the Day 78 of Study ALT-801-105 (2 weeks prior to the screening visit for Study ALT-801-106 on Day -7), including:
   a. Impaired renal function (estimated glomerular filtration rate [eGFR] < 60 mL/min/1.73 m^2) as estimated using the Modification of Diet in Renal Disease (MDRD) equation:

      GFR (mL/min/1.73 m^2) = 175 × (SCr/88.4)-1.154 × (Age)-0.203 × (0.742 if female) × (1.212 if African American) (SI units), where SCr is standardized serum creatinine in SI units (µmol/L) and age is in years
   b. Alanine aminotransferase (ALT) or aspartate aminotransferase (AST) laboratory values > 75 U/mL
   c. Alkaline phosphatase values > 2 × upper normal limits
   d. Total bilirubin > 1.3 mg/dL, with the exception of Gilbert Syndrome
   e. International normalized ratio (INR) > 1.3
   f. Hemoglobin < 11.0 g/dL
   g. Platelet count < 150,000/µL
   h. Any other abnormality deemed by the Investigator to exceed normal safety limits for this study or exclude subject participation

   Subjects not meeting any of these exclusion criteria may be retested at the discretion of the Medical Monitor.

6. Subjects who, in the opinion of the Investigator, are unlikely to comply with the study protocol or would not be a suitable candidate for participation in the study.

6. STUDY MEDICATION

6.1. Description

6.1.1. Formulation

6.1.1.1. ALT-801

ALT-801 is a peptide-based dual GLP-1/glucagon receptor agonist. It is formulated in glass vials in a sterile, buffered aqueous solution to a final concentration of 2.5 mg/mL and total fill volume of 1.2 mL. Further information is provided in the Investigators Brochure and Pharmacy Manual.

ALT-801 is administered by SC injection.

6.1.1.2. Placebo

Normal saline (0.9% NaCl) for SC injection will be supplied by the Sponsor.

6.1.2. Packaging, Storage, and Handling

All supplies are labelled as investigational products in accordance with applicable legal and regulatory requirements. All investigational products will be stored in a secure place under appropriate storage conditions.

Full preparation instructions are detailed in a separate Pharmacy Manual.

6.2. Randomization

Three is no randomization in this extension study. The treatment a subject receives in this study will be dependent on the randomly assigned treatment group from Study ALT-801-105.

6.3. Dose and Administration

Study medication will be administered weekly for 12 weeks. Subjects who received active treatment with ALT-801 in Study ALT-801-105 will continue to receive ALT-801 at the same dose; those that received placebo in Study ALT-801-105 will continue to receive placebo. Thus, treatment groups in this extension study are as follows:

- ALT-801 1.2 mg SC once weekly for 12 weeks
- ALT-801 1.8 mg SC once weekly for 12 weeks
- ALT-801 2.4 mg SC once weekly for 12 weeks
- Placebo SC once weekly for 12 weeks

Each dose of ALT-801 or placebo will be administered as an SC injection in the abdominal region by appropriately trained clinical staff members. The volume of administration will be based on the assigned dose and a concentration of 2.5 mg/mL for the final drug product.

6.4. Dosing Modifications

As weight loss is a desired property of this compound, it will be monitored for efficacy rather than safety. However, study medication may be paused or discontinued in individual subjects if the level of weight loss is considered excessive.

Study medication may also be paused or discontinued in individual subjects if the level of GI AEs is considered excessive and intolerable despite antiemetic treatment (eg, severe GI AEs continue > 24 hours). If there is persistent vomiting a subject may be given an antiemetic. Algorithms for liver function abnormalities and glucose targets are provided in Appendix 2 and Appendix 3. Subjects who miss more than 2 continuous weeks of dosing should permanently discontinue study medications.

Subjects who prematurely discontinue study medication will remain in the study for early termination (ET) study assessments, with the date of visit adjusted to correspond to 25 days after the last dose of study medication, where the day of dosing is counted as the first day.

6.5. Blinding and Unblinding

The Pharmacy staff will be unblinded for the purpose of final drug preparation. The pharmacist will consult the IWRS for dose allocation, which will be determined by the unblinded study statistician. The pharmacy staff will prepare each dose in compliance with the randomization list.

Investigational Product is manufactured at a fixed concentration and volume varies based on randomization arm. Study staff preparing and administering the Investigational Product will be independent and will not take part in any other activity of the study (eg, clinical assessments).

Knowledge of the randomization list will be limited to the persons responsible for creation of the randomization list, pharmacy staff who prepare the study medications, and any unblinded study monitors or auditors, until all data has been entered in the electronic case report form (eCRF), quality control and verification of the eCRF and assignment of subjects to the analysis populations has been completed, the database has been locked, and the study formally unblinded.

Data provided to the Safety Assessment Committee (SAC) will be blinded (See Section 9.8)

If unblinding is required in the interest of the safety of a subject, an Investigator will discuss the matter with the Sponsor before unblinding. In a medical emergency, the Investigator or delegate may unblind via the IWRS for that subject without prior consultation with the Sponsor. In that event, the Investigator or delegate will notify the Sponsor as soon as possible that the randomization code has been broken for the subject. If the blind is broken, the date, time, and reason must be recorded.

Subjects may also be unblinded for the assessment of Stopping Rules and the relationship of study medication to AEs, the processing of serious AEs (SAEs), expedited safety reports, and the emergency unblinding of subjects, as detailed in a separate Safety Management Plan (SMP).

6.6. Accountability

The Investigator (or designee) will maintain an accurate record of the receipt of the study medication as shipped by the Sponsor (or designee), including the date received. In addition, an

accurate study medication disposition record will be kept, specifying the amount dispensed for each subject and the dates of dispensation and any returns.

Sponsor approval is required for on-site destruction of all used study medication and shipment of all unused study medication back to the Sponsor at the completion of the study and once all reconciliation has occurred.

6.7. Prior and Concomitant Therapy

In the interests of safety and acceptable standards of medical care, the Investigator will be permitted to prescribe additional treatment(s) at his/her discretion (eg, for intolerable GI symptoms, antiemetic medication will be allowed per PI discretion, see Section 6.4). However, as noted below, certain restrictions apply.

On the basis of in vitro DDI studies, no effects on CYP induction or inhibition are anticipated, and significant transporter interactions, while possible, are unlikely (Section 1.2).

All medications must be recorded in the subject's electronic case report forms (eCRFs) from the day of the first dose in this study through the follow-up visit (Day 110) or early termination from the study, as indicated in Table 1.

The following medications are prohibited concomitant with the study, through the follow-up visit:

- Insulin
- Any GLP-1 agonist or other any injectable diabetes treatment
- Anti-obesity medications
- Sulfonylureas, pioglitazone, or DPP-4 inhibitors
- Medications that can induce steatosis/steatohepatitis including, but not limited to, chronic systemic corticosteroids, methotrexate, amiodarone, and tamoxifen
- Herbal remedies (such as St. John's Wort)
- Vitamin E
- Medications that may alter gastric emptying or stool frequency, except as prescribed for the treatment of GI AEs such as, but not limited to nausea, vomiting, or diarrhea that occur during treatment

The following medications are allowed concomitant with the study, with restrictions:

- Acetaminophen may affect glucose readings; therefore, the dose must be limited to up to 1000 mg every 6 hours during the study
- Antihypertensive and lipid lowering medications are permitted as long as they are stable and are not expected to change over the course of the study. Any change in dose or schedule must be recorded in eCRF.

6.8. Contraception

Refer to Inclusion Criterion 4 in Section 5.2.

6.9. Compliance

The injections will be administered by study personnel and therefore compliance with study medication dosing is not a concern. The study personnel will be appropriately trained on study medication administration procedures and documentation requirements prior to study start.

Non-compliance with study procedures will be reported to the Sponsor who will decide if persistent non-compliant subjects should be withdrawn from continued study treatment.

7. PREMATURE DISCONTINUATION

7.1. Individual Subjects

Subjects can choose to discontinue study medication or participation in the study at any time, for any reason, without prejudice to their future medical care. Subjects could be discontinued for any of the following reasons:

- Subject request/withdrawal of consent
- Noncompliance with study requirements
- Loss to follow-up
- Adverse event(s), including hyperglycemia, hypoglycemia and/or laboratory abnormalities
- Sponsor request, including termination of the study by the Sponsor

Subjects who prematurely discontinue study medication will remain in the study for early termination (ET) study assessments, with the date of visit adjusted to correspond to 25 days after the last dose of study medication, where the day of dosing is counted as the first day.

7.2. Stopping Rules

7.2.1. Individual Subject Stopping Rules

Subjects will be discontinued from receiving treatment for any of the following reasons:

- Any Grade 3 or 4 AE according to Common Terminology Criteria for Adverse Events [CTCAE, US Department of Health and Human Services 2017] that is possibly or probably related to study drug

Subjects who prematurely discontinue study medication will remain in the study for early termination (ET) study assessments, with the date of visit adjusted to correspond to 25 days after the last dose of study medication, where the day of dosing is counted as the first day.

7.2.2. Study Stopping Rules

The study will be paused, and causality assessed before resumption, if any one of the following three categories of AEs, as defined by CTCAE, occur:

- One fatal (Grade 5) event that is possibly or probably related to study drug
- Two Grade 4 events that are possibly or probably related to study drug
- Three Grade 3 events that are possibly or probably related to study drug

7.3. Study Termination

The Sponsor reserves the right to terminate the study at any time. Should this be necessary, the Sponsor or a specified designee will inform the appropriate regulatory authorities of the termination of the study and the reasons for its termination, and the Investigator will inform the

Institutional Review Board (IRB) / Independent Ethics Committee (IEC) of the same. In terminating the study, the Sponsor and the Investigator will assure that adequate consideration is given to the protection of the subjects’ interests.

Possible reasons for termination are:

- Safety reasons –the incidence of AEs in this or any other study using the same study medication indicates a potential health risk for the subjects
- New scientific knowledge becomes known that makes the objectives of the study no longer feasible/valid
- Unsatisfactory enrollment of subjects
- Sponsor decision

8. DESCRIPTION OF STUDY PROCEDURES

See Section 4.5 for the schedule of study assessments. Signed informed consent will be obtained on the Day 78 visit of Study ALT-801-105, at which time screening for this extension study will commence. The Day 85 visit of Study ALT-801-105 represents the Day 1 visit of this extension study. The scheduled procedures on Days 78 and Day 85 of Study ALT-801-105 will be performed, supplemented by the procedures noted for this extension study.

8.1. Pharmacodynamic Assessments

8.1.1. Weight, Waist Circumference, and Body Mass Index (BMI)

Weight and waist circumference will be measured, and BMI calculated and recorded according to the schedules in Table 1.

Weight measurements should be taken with subjects wearing a gown (or other standard clothing provided by the clinical research unit), undergarments, and socks (no shoes), while fasting and after the subject has been asked to void (ie, empty bladder).

Waist circumference should be taken with the subject wearing a gown. The measurement will be performed at a level midway between the superior aspect of the iliac crests and the lower lateral margin of the ribs. The measurement need not be at the level of the umbilicus. The measuring tape will be kept horizontal.

8.1.2. Lipids

Following a minimum 8 hour fast, blood will be collected for assessment of lipids, including cholesterol (total, HDL, LDL), Apo A and B, lipoprotein(a), and TG, as specified in the schedule of assessments or within ±2 days before the scheduled timepoint (Table 1).

Please refer to the current version of the Laboratory Manual for details.

8.1.3. Metabolic Markers

Blood will be collected for the assessment of metabolic markers, including HbA1c, adiponectin and leptin, as specified in the schedule of assessments (Table 1).

Please refer to the current version of the Laboratory Manual for details.

8.1.4. Inflammatory Markers

Blood will be collected for the assessment of inflammatory markers, including TNF, hs-CRP, MCP-1, IL-6, and PAI-1, as specified in the schedule of assessments (Table 1).

Please refer to the current version of the Laboratory Manual for details.

8.1.5. Fibrosis Markers

Blood will be collected for the assessment of fibrosis markers, including Pro-C3 and ELF, as specified in the schedule of assessments (Table 1).

8.1.6. Lipotoxicity Markers

Blood will be collected for the assessment of lipotoxicity markers, as specified in the schedule of assessments (Table 1). The specific lipids to be analyzed will be described in a separate analysis plan.

8.1.7. Stored Samples for Future Analysis

Blood samples for storage will be collected as indicated in Table 1, to be used for future analyses not specified in the protocol.

8.1.8. Imaging

FibroScan, MRIs for body composition, MRI-PDFF and cT1 will be collected according to the schedule of assessments (Table 1). All imaging will be performed following an overnight fast of at least 8 hours.

FibroScan is an ultrasound-like instrument able to simultaneously measure liver stiffness and steatosis through Vibration-Controlled Transient Elastography (VCTE) and CAP, respectively.

Body composition will also be assessed by MRI scanning. The body composition parameters will be delineated in the MRI Manual.

Corrected T1 is an MRI procedure that assesses the fibro-inflammatory activity of the liver. It applies a correction for liver iron that confounds the T1 image assessment.

Please refer to the current version of the FibroScan®, MRI and cT1 manuals for details.

8.2. Quality of Life Assessments

Quality of life will be assessed on Day 85 or early termination by the SF-36 and IWQoL-Lite for CT. The IWQoL-Lite for CT is a 20-item modified version of a questionnaire tool designed to assess the weight-related quality of life. The SF-36 measures the subject's overall health related quality of life. It is a 36-item generic measure of health status that yields 2 summary scores for physical health and mental health, and 8 domain scores.

8.3. Immunogenicity Assessment

Anti-ALT-801 antibodies will be evaluated in serum samples collected from all subjects at the follow-up visit on Day 110. If an AE requires follow-up beyond the follow-up visit at the end of the study, additional samples will be collected monthly or at resolution, if sooner, to assess the potential relationship to anti-drug antibodies.

Please refer to the current version of the Immunogenicity Manual for details.

8.4. Dietary and Exercise Counseling

Subjects will be instructed how to maintain their normal diets, alcohol consumption and physical activities and not to start any new diets, supplements, or exercise programs at any time while participating in the study. Counseling will be reinforced at each weekly visit during treatment as indicated in Table 1.

8.5. Safety Assessments

8.5.1. Adverse Events

Adverse events will be assessed by direct observation and subject assessments/interviews as specified in the schedule of assessments (Table 1). Details on the definitions, reporting, and management of AEs are provided in Section 9.

8.5.2. Safety Laboratory Tests

Blood samples for hematology, chemistry and coagulation laboratory tests (following a minimum 8 hour fast) and urine samples for urinalysis will be collected according to the schedule of assessments listed in Table 1. Safety laboratory tests are detailed in Appendix 1.

Abnormal laboratory results considered to be clinically significant should be repeated as soon as possible (preferably within 24 to 48 hours). Abnormal liver function tests will be monitored as delineated in Appendix 2.

Urine drug screen, alcohol breath test, and blood samples for calcitonin will be collected as indicated in Table 1.

Please refer to the current version of the Laboratory Manual for details.

8.5.3. Glucose Monitoring

Fasting glucose levels will be measured by a glucometer and documented by study staff prior to each dose as indicated in Table 1. On non-visit days, subjects will also monitor and record glucose levels by glucometer readings each morning and will contact the study site for a reading > 240mg/dL or < 70mg/dL. Subjects will also be educated on symptoms and treatment of hypoglycemia and will obtain a glucometer reading if they experience plasma glucose < 70 mg/mL or symptoms suggestive of hypoglycemia, as described in Appendix 3. Subjects will record any symptoms of hypoglycemia experienced at home in a log, which will be reviewed by the Investigator at each visit.

Investigators will counsel subjects on how to keep their glucose levels within the limits, including repeated diet counseling, and will follow the decision criteria for the timing and method of intervention in subjects with persistent hyperglycemia during the 12-week treatment period (Appendix 4).

If a significant decrease of glucose by laboratory testing or glucometer is repeatedly observed (fasting glucose < 50 mg/dL) or a subject requires interventions or external assistance to treat hypoglycemia, the subject may be dropped from the study.

8.5.4. Pregnancy Tests

Urine pregnancy tests are required according to the schedule of assessments listed in Table 1.

8.5.5. Physical Examination

Physical examinations will be performed by a physician or qualified designee. A complete physical examination will include an examination of general appearance, skin, neck (including

thyroid), eyes, ears, nose, throat, lungs, heart, abdomen, back, lymph nodes, extremities, and basic nervous system evaluation. An abbreviated physical examination is a symptom-targeted physical examination that includes the heart, lungs, plus any other body system directed by the symptoms reported by the subject, if any.

A complete physical examination will be performed at the follow-up visit and abbreviated physical examinations will be performed at other time points, as indicated in Table 1. Additional abbreviated exams may be performed during study if deemed necessary by the Investigator. Any clinically relevant change in physical examination findings will be recorded as AEs.

8.5.6. Vital Signs

Vital signs will be done in the seated or semi-recumbent position and will include blood pressure, heart rate (after at least 5 minutes rest), respiratory rate, and body temperature, and will be assessed per Table 1. At visits with laboratory assessments, vital signs should be measured before any blood sample collection. Any clinically relevant change in vital signs will be recorded as AEs. RPP will be calculated as mean heart rate × mean systolic blood pressure.

8.5.7. Electrocardiograms

Twelve-lead ECGs for safety will be generated on designated days (Table 1).

Subjects will be required to be semi-recumbent and abstain from external stimulus for at least 5 minutes during the ECG preparation and collection period. When multiple activities occur at the same timepoint, ECGs should be collected first, followed by other assessments and blood draws. Any clinically relevant changes in ECG findings will be recorded as AEs.

9. ADVERSE EVENTS

9.1. Definitions

9.1.1. Adverse Event

An AE is defined as any untoward medical occurrence associated with the use of a drug in humans, whether or not considered drug related. An AE can therefore be any unfavorable and unintended sign (including an abnormal laboratory finding), symptom, or disease temporally associated with the use of the investigational product whether or not related to the investigational product. An AE can be any sign, symptom, or diagnosis that appears or changes in intensity during the course of the study.

Unchanged chronic conditions are not AEs and should not be recorded on the AE pages of the eCRF. These medical conditions should be adequately documented on the appropriate page of the eCRF (medical history or physical examination). However, medical conditions present on the first day of treatment that worsen in intensity or frequency during the treatment or post-treatment periods in a manner not consistent with natural disease progression should be reported and recorded as AEs. The Investigator will actively solicit this information and assess the event in terms of severity and relationship to the study treatment regimen.

Anorexia, loss of appetite and weight loss are the intended effects of treatment and will not be characterized as AEs.

An isolated elevation of amylase and/or lipase in the absence of symptoms of pancreatitis will not be reported as an AE.

The term AE is used to include any AE whether serious or not serious.

9.1.2. Adverse Drug Reaction

All noxious and unintended responses to a medicinal product related to any dose should be considered an adverse drug reaction. A reaction means that a causal relationship between a medicinal product and an AE is at least a reasonable possibility, ie, the relationship cannot be ruled out.

9.1.3. Unexpected Adverse Drug Reaction

An unexpected adverse drug reaction is defined as an adverse reaction, the nature or severity of which is not consistent with the reference safety information in the Investigator's Brochure.

For the purpose of expedited reporting of unexpected serious adverse drug reactions (ie, Suspected Unexpected Serious Adverse Reactions [SUSARs]), only possibly or probably related and unexpected SAEs (Section 9.3.2) will be considered serious adverse drug reactions.

9.1.4. Serious Adverse Event

An AE or suspected adverse reaction is considered serious (an SAE) if, in the view of either the Investigator or Sponsor, it results in any of the following outcomes:

- Death
- Life-threatening
- Inpatient hospitalization or prolongation of existing hospitalization
- A persistent or significant incapacity or substantial disruption of the ability to conduct normal life functions
- A congenital anomaly/birth defect

Life-threatening means that the patient or subject was at immediate risk of death at the time of the SAE; it does not refer to a serious AE that hypothetically might have caused death if it were more severe. Hospitalization does not include same day surgery, elective surgery, optional admission not associated with a precipitating AE (ie, elective cosmetic surgery), or hospitalization planned before the start of the study for a pre-existing condition that has not worsened. Persistent or significant disability or incapacity means that there is a substantial disruption of a person's ability to carry out normal life functions.

Important medical events that may not result in death, be life-threatening, or require hospitalization may be considered serious when, based upon appropriate medical judgment, they may jeopardize the patient or subject and may require medical or surgical intervention to prevent one of the outcomes listed in this definition. Examples of such medical events include allergic bronchospasm requiring intensive treatment in an emergency room or at home, blood dyscrasias or convulsions that do not result in inpatient hospitalization, or the development of drug dependency or drug abuse.

9.2. Reporting Responsibilities and Periods

It is the responsibility of the Investigator or Sub-investigator(s) to perform periodic assessment of AEs. AEs spontaneously reported by the subject or reported in response to an open question from the study personnel (eg, 'Have you had any health problems since the previous visit/you were last asked?') or revealed by observation will be recorded.

All AEs and concomitant medications will be recorded from the time of informed consent through the Follow-up Visit. The AE term, date of AE onset, date of AE resolution (if applicable), severity, causality, action taken for the AE, outcome and whether or not the AE is an SAE will be recorded.

AEs must be monitored until they are resolved or stabilized, are clearly determined to be due to a subject's stable or chronic condition or intercurrent illness(es), or follow-up is no longer possible. Data describing AEs will be recorded in the subject's medical record and, as appropriate, an SAE report form. SAEs or Grade 3 or higher AEs will be reported to the Sponsor as described in Section 9.7.

Any SAE that the Investigator considers to be related to study medication and occurs at any time after completion of the study must be reported to the Sponsor or designee. If at the time the Investigator initially reports an SAE, the event has not resolved, the Investigator must provide a follow-up report as soon as it resolves (or upon receipt of significant information if the event is still ongoing).

9.3. Assessment of Adverse Events

9.3.1. Severity

The Investigator should assess the severity of each AE. The AE will be recorded at its initial severity level. The initial AE will be considered ended, and a new AE will be recorded if the event changes in severity.

The severity of all AEs, both serious and non-serious, including injection site reactions, will be assessed by assigning a grade of 1, 2, 3, or 4 using the CTCAE.

When an AE cannot be graded by according to the above grading scales, the following severity grading may be used:

- Grade 1 (Mild): awareness of sign or symptom, but easily tolerated
- Grade 2 (Moderate): discomfort enough to cause interference with usual activity
- Grade 3 (Severe): incapacitating with inability to work or do usual activity
- Grade 4 (Potentially Life-Threatening): refers to an event in which the subject was, in the view of the Investigator, at risk of death at the time of the event. (This category is not to be used for an event that hypothetically might have caused death if it were more severe.)
- Grade 5 (Death): refers to any subject death during study participation. An AE that is assessed as severe should not be confused with an SAE. Severity is a category for rating the intensity of an event, and both non-serious AEs and SAEs can be assessed as severe. An event will be defined as serious when it meets one of the criteria described in Section 9.1.4.

9.3.2. Relatedness (Causality)

The assessment of causality will be based on the information available and may be changed upon receipt of additional information.

Causality should be assessed using the following categories:

- Unlikely related: clinical event with an incompatible time relationship to investigational agent administration, and that could be explained by underlying disease or other drugs or chemicals or is incontrovertibly not related to the investigational agent

- Possibly related: clinical event with a reasonable time relationship to investigational agent administration, and that is unlikely to be attributed to concurrent disease or other drugs or chemicals
- Probably related: clinical event with plausible time relationship to investigational agent administration, and that cannot be explained by concurrent disease or other drugs or chemicals

9.4. Safety Laboratory, Physical Examination, Electrocardiogram, and Vital Sign Abnormalities

Any abnormal laboratory result, physical examination finding, ECG interpretation, or vital sign measurement considered clinically significant by the Investigator will be recorded as an AE. A clinically significant laboratory abnormality is a confirmed abnormality (by repeat test) that is changed sufficiently from baseline so that in the judgment of the Investigator a change in management is warranted. This alteration may include monitoring the laboratory test further, initiating other diagnostic tests or procedures, changing ongoing treatment, or administering new treatment.

Whenever possible, the underlying medical diagnosis (eg, anemia) will be recorded as the AE term. Repeated additional assessments required to establish the significance and etiology of an abnormal result should be obtained when clinically indicated.

9.5. Pregnancy

Pregnancy itself is not regarded as an AE unless there is a suspicion that the study medication may have interfered with the effectiveness of a contraceptive medication. Pregnancy in a subject's partner is not considered an AE. Congenital abnormalities/birth defects and spontaneous miscarriages should be reported and handled as SAEs. Elective abortions without complications should not be handled as AEs. The outcome of a pregnancy will be followed-up and documented even if the subject was withdrawn from the study. See Section 9.7.4 for further information on reporting of pregnancy.

An induced elective abortion to terminate a pregnancy without medical reason is not regarded as an AE. However, an induced therapeutic abortion to terminate a pregnancy because of complications or medical reasons must be reported as an SAE. The underlying medical diagnosis for this procedure should be reported as the SAE term. A spontaneous abortion in a study subject is always considered an SAE.

9.6. Overdose

Any instance of overdose (suspected or confirmed) and irrespective of whether or not it involved any study medication must be communicated to the Sponsor or a specified designee within 24 hours. Details of any signs or symptoms and their management should be recorded including details of any antidote(s) administered. An overdose in and of itself will not be treated as an SAE. If an SAE occurs due to an overdose, that will be reported as a SAE. An overdose of study medication is not expected, as it is administered via SC injection. Should subjects receive a

higher dose than the allocated dose, this should be reported in the eCRF, and the Sponsor should be informed. Any deviations from the assigned dose will be handled as a protocol deviation.

9.7. Procedures for Recording and Reporting Adverse Events

9.7.1. Recording Adverse Events

To improve the quality and precision of AE data, Investigators should observe the following guidelines:

- Whenever possible, use recognized medical terms when recording AEs on the AE page of the eCRF. Do not use colloquialisms, jargon, or abbreviations.
- If known, record the diagnosis (ie, disease or syndrome) rather than component signs and symptoms on AE pages of the eCRF (eg, record “congestive heart failure” rather than “dyspnea”, “rales”, and “cyanosis”). However, signs and symptoms that are considered unrelated to an encountered syndrome or disease should be recorded as individual AEs on the eCRF page. For example, if congestive heart failure and severe headache are observed at the same time, each event should be recorded as an individual AE.
- Adverse events occurring secondary to other events (ie, sequelae) should be identified by the primary cause. A primary AE, if clearly identifiable, generally represents the most accurate clinical term to record on the AE page of the eCRF. If a primary SAE is recorded on an AE eCRF page, events occurring secondary to the primary event should be described in the narrative description of the event.
- Laboratory abnormalities, physical examination finding, ECG interpretation, or vital signs that are identified by the Investigator as clinically significant are to be considered AEs and recorded on the AE eCRF page.

9.7.2. Reporting of Serious Adverse Events or Adverse Events Meeting Criteria for Individual Subject Stopping Rules

All SAEs as defined in Section 9.1.4 and Grade 3 or higher AEs as defined in Section 9.3.1, require reporting within 24 hours, regardless of the relationship of the event to the study treatment regimen.

If the SAE is fatal or life-threatening, notification to the sponsor designee must be made immediately, irrespective of the extent of available AE information.

The SAE Form should be completed and signed by the Principal Investigator (with support from site staff) as thoroughly as possible with all available details of the event, including a determination of causality (even if preliminary).

The Investigator will complete an initial SAE Form with information available and notify the sponsor designee. The Investigator should contact the sponsor designee if there are questions regarding the reporting of an SAE or if any information needs to be transmitted that cannot be recorded on the SAE forms (eg, medical records, discharge summaries, laboratory reports).

For SUSARs, [blinded] reports will be disseminated and provided to Investigators at each study site. When required and according to local law and regulations, [unblinded] SUSARs or other SAEs will be reported by a[n unblinded] designee to the IRB/IEC and regulatory authorities.

If not all information regarding an SAE is initially available, the Investigator should not wait to receive additional information before completing the AE eCRF and SAE forms. For initial SAE reports, the Investigator should record all case details that can be garnered on the SAE form and the AE eCRF page. Relevant follow-up information is to be submitted on updated SAE forms/entered on the AE eCRF page as soon as it becomes available.

9.7.3. Special Reporting Situations

Death

Death is an outcome of an event. The event that resulted in death should be recorded and reported on the SAE form and the AE eCRF page.

Surgical or Diagnostic Procedures

The illness leading to a surgical or diagnostic procedure is to be recorded as an AE/SAE, not the procedure itself. The procedure is to be captured in the case narrative as part of the action taken in response to the illness.

9.7.4. Reporting Pregnancies

Pregnancy itself is not considered an AE. If a **subject becomes pregnant** during the study or **within 1 month of discontinuing** any study medication or the **partner of a subject** participating in the study becomes pregnant during the study or **within 1 month of discontinuing** any study medication, the Investigator should report the pregnancy on a separate Pregnancy Report Form provided to the study site. Only pregnancies occurring from the time of first study medication dose administered to the subject will be reported and documented. However, any pregnancy complication, spontaneous or elective abortion, still birth, neonatal death, or congenital anomaly will be recorded as an AE or SAE, and reported, as applicable.

The pregnancy must be reported within the same timeframe and following the same process that applies to SAEs, using the Pregnancy Report Form included in the SMP and sending the completed Pregnancy Reporting Form to: PVsafety@prosciento.com

9.8. Safety Assessment Committee

The Safety Assessment Committee (SAC) will conduct regular reviews of all AEs and monitor trends in laboratory abnormalities across this study and pooled aggregate data of all studies involving the use of ALT-801. The responsibilities of the SAC are delineated in the Sponsor's SOP REG-002.

Based on the pharmacology and safety experience with GLP-1 and GLP-1 and glucagon dual agonists, the frequency and severity of GI (nausea and vomiting) AEs will be monitored closely. Liver function tests will be monitored in aggregate for trends suggesting hepatotoxicity, and glucose homeostasis will also be evaluated according to the severity and incidence of hyperglycemia and hypoglycemia.

9.9. Medical Monitor

Medical Monitors will provide full-time coverage for medical issues pertaining to this study, including but not limited to Inclusion and Exclusion Criteria, protocol deviations, and issues affecting the safety of study participants.

10. STATISTICS

10.1. General Procedures

Baseline for this study is defined as data collected on the Day 85 visit of Study ALT-801-105 and prior to the dosing on Day 1 of this study. Descriptive statistics, including the numbers and percentages for categorical variables and the numbers, means, standard deviations, medians, minimums and maximums for continuous variables will be provided by dose and treatment, and by day when applicable.

Two sets of baseline comparisons will be conducted: 1) comparisons to the baseline of Study ALT-801-105; and 2) comparisons to the baseline for this study. Further details will be provided in the Statistical Analysis Plan (SAP).

10.2. Power and Sample Size Assumptions

This is an extension study that is being offered to participants of Study ALT-801-105, and no sample size calculation is applicable.

10.3. Analysis Sets

Safety Population:	All subjects who receive at least 1 dose of study medication.
PD Population:	All subjects who receive at least 1 dose of study medication and who have results for at least 1 post-baseline PD assessment.

10.4. Statistical Methods

10.4.1. Safety/Tolerability

A medical occurrence will be reported as a treatment-emergent AE (TEAE) if it represents 1) a new occurrence since the first dose of study medication on Day 1, 2) worsening in the severity of a previously reported AE in this study or Study ALT-801-105, or 3) the development of seriousness criteria in a previously reported AE. Adverse events that occur before the first dose of study medication on Day 1 will be accrued to Study ALT-801-105; those that occur after the first dose of study medication on Day 1 will be accrued to this study.

Continuous safety data will be summarized with descriptive statistics (arithmetic mean, standard deviation [SD], median, minimum, and maximum) by dose level. Categorical safety data will be summarized with frequency counts and percentages by dose level and day where applicable.

AEs will be coded using the most current Medical Dictionary for Regulatory Activities (MedDRA) version. A by-subject AE data listing, including verbatim term, preferred term, system organ class (SOC), treatment, severity, and relationship to study medication, will be provided. The number of subjects experiencing treatment-emergent AEs (TEAEs) and number of individual TEAEs, and injection site reactions will be summarized by treatment group, SOC, and preferred term. TEAEs will also be summarized by severity and relationship to study medication.

Laboratory evaluations, including liver function tests and FPG, vital signs (including calculation of RPP), and ECG parameters will be summarized by treatment group, dose level, and protocol

specified collection time point. A summary of change from baseline at each protocol specified time point by treatment group will also be presented.

Changes in physical examinations from baseline will be listed for each subject.

Concomitant medications will be listed by subject and coded using the most current World Health Organization (WHO) Drug Dictionary.

10.4.2. Pharmacodynamics

Descriptive statistics, including the numbers and percentages for categorical variables and the numbers, means, SDs, medians, minimums, and maximums for continuous variables will be provided by dose and by day when applicable.

Changes from baselines in MRI-PDFF, anthropometric parameters and body composition, lipid metabolism, metabolic markers, fibrosis markers, lipotoxicity markers, inflammation markers, FibroScan, and cT1 (in subjects for whom cT1 was performed) will be listed and summarized by treatment group and strata with descriptive statistics (sample size [N], arithmetic mean, SD, median, minimum, maximum, geometric mean, and geometric coefficient of variation [CV%]). The effects of baseline BMI on PD parameters will be evaluated by covariate analyses.

10.4.3. Quality of Life

Changes from baseline in the 2 summary scores for physical health and mental health, and 8 domain scores for SF-36 and the composite score for the IWQoL-Lite for CT will be listed and summarized by treatment group with descriptive statistics (sample size [N], arithmetic mean, SD, median, minimum, maximum, geometric mean, and geometric coefficient of variation [CV%]). Inferential statistics applicable to continuous endpoints will be applied, as described in Section 10.4.2.

10.5. Statistical Analysis Plan

A formal SAP will be developed and finalized prior to the database lock. This plan will confirm the analysis sets used in the analysis, outline all data handling conventions, and specify all statistical methods to be used for all analyses, including an interim analysis conducted after all subjects complete 12 weeks of treatment. The SAP will supersede the protocol with respect to analyses specified, although the primary analysis will remain the same. It is anticipated that analyses other than those specified in the protocol may be pre-specified in the SAP.

11. DATA QUALITY ASSURANCE

Accurate, consistent, and reliable data will be ensured through the use of standard practices and procedures. These are described in the following sections.

11.1. Data Handling

Data will be recorded at the site on source documents and reviewed by the Clinical Research Associate (CRA) during monitoring visits. The CRA will verify data recorded in the eCRF system with source documents. All corrections or changes made to any study data must be appropriately tracked in an audit trail in the eCRF system. The eCRFs will be considered complete when all missing, incorrect, and/or inconsistent data have been reconciled.

11.2. Computer Systems

Data will be processed using a validated computer system conforming to regulatory requirements.

11.3. Data Entry

Data must be recorded using the eCRF system as the study is in progress. All study site personnel must log into the system using their secure username and password in order to enter, review, or correct study data. These procedures must comply with 21 CFR Part 11 and other appropriate international regulations. All passwords will be strictly confidential.

11.4. Medical Information Coding

For coding medical information, the current versions of MedDRA will be used for AEs and medical histories and WHO Drug Dictionary for concomitant medications.

11.5. Data Validation

Validation checks programmed within the eCRF system, as well as supplemental validation performed via review of the downloaded data, will be applied to the data in order to ensure accurate, consistent, and reliable data. Data identified as erroneous, or data that are missing, will be referred to the investigative site for resolution through data queries.

The eCRFs must be reviewed and electronically signed by the Investigator who signed the protocol.

11.6. Study Monitoring Requirements

It is the responsibility of the Investigator to ensure that the study is conducted in accordance with the protocol, Declaration of Helsinki, ICH GCP guidelines, and applicable regulatory requirements, and that valid data are entered into the eCRFs.

The Investigator will permit the Sponsor or their designee to monitor the study as frequently as deemed necessary to determine that data recording and protocol adherence are satisfactory. Monitoring will include on-site review of the eCRFs for completeness and clarity, cross-checking with source documents, and clarification of administrative matters. The review of

medical records will be performed in a manner to ensure that subject confidentiality is maintained. All monitoring activities will be reported and archived. In addition, monitoring visits will be documented at the investigational site by CRA signature and date on the study-specific monitoring log and the completion of a detailed monitoring report.

The CRA will ensure that the investigation is conducted according to protocol design and regulatory requirements by frequent communications with the investigational site (monitoring visits, letter, telephone, email, and fax).

All unused study medication and other study materials are to be returned to the Sponsor or designee after the clinical phase of the study has been completed.

Regulatory authorities, the IRB/IEC, and/or the Sponsor's clinical quality assurance group may request access to all source documents, eCRFs, and other study documentation for on-site audit or inspection. Direct access to these documents must be guaranteed by the Investigator, who must provide support at all times for these activities.

11.7. Source Document and Case Report Form Completion

Source data is defined as all information in original records and certified copies of original records of clinical findings, observations, or other activities in a clinical study necessary for the evaluation and reconstruction of the clinical study. Source data are contained in source documents (ie, original records or certified copies). Source documents and the eCRFs will be completed for each study subject. It is the Investigator's responsibility to ensure the accuracy, completeness, and timeliness of the data reported in the subject's source document/eCRF. The source document/eCRF should indicate the subject's participation in the study and should document the dates and details of study procedures, AEs, and subject status.

The Investigator, or designated representative, should complete the source document/eCRF as soon as possible after information is collected, preferably on the same day that a study subject is seen for an examination, treatment, or any other study procedure. Any outstanding entries must be completed immediately after the final examination. An explanation should be given for all missing data.

The Investigator must sign and date the Investigator's Statement at the end of the source document/eCRF to endorse the recorded data.

The Investigator will retain all completed source documents. A site-specific eCRF archive and audit trail will be provided at the close of the study to each Investigator. The Sponsor or designee will retain the eCRF archive and audit trail for the investigative site.

11.8. Record Retention

Records of subjects, source documents, monitoring visit logs, eCRFs, inventories of study product, regulatory documents, and other correspondence pertaining to the study must be kept in the appropriate study files at the site. The Investigator will maintain all study records according to ICH GCP and applicable regulatory requirements. Records will be retained for at least 2 years after the last marketing application approval or 2 years after formal discontinuation of the clinical development of the investigational product or according to applicable regulatory

requirements. If the Investigator withdraws from the responsibility of keeping the study records, custody must be transferred to a person willing to accept the responsibility. The Sponsor must be notified in writing if a custodial change occurs.

12. ETHICS

12.1. Good Clinical Practice

The study will be conducted in accordance with the protocol, GCP, the relevant ICH guidelines, the applicable regulatory requirements, and the ethical principles that have their origins in the Declaration of Helsinki. As required by US FDA (21 CFR 56) and the Declaration of Helsinki, the study protocol, amendments, and informed consent form will be reviewed and approved, according to ICH E6, by each study center's IRB or IEC.

12.2. Institutional Review Board / Independent Ethics Committee

The IRB/IEC will review all appropriate study documentation in order to safeguard the rights, safety, and wellbeing of subjects. Federal/local regulations and ICH GCP guidelines require that approval be obtained from an IRB/IEC prior to participation of subjects in research studies. The study will only be conducted at a site where IRB/IEC approval has been obtained. The protocol, Investigator's Brochure, informed consent form, advertisements (if applicable), written information given to the subjects, safety updates, annual progress reports, and any revisions to these documents will be provided to the IRB/IEC by the Investigator.

No drug will be released to the site for dosing until written IRB/IEC authorization has been received by the Sponsor.

12.3. Subject Information and Consent

The informed consent form and any changes to the informed consent form made during the course of the study must be agreed to by the Sponsor or their designee and the IRB/IEC prior to its use and must be in compliance with ICH GCP guidelines, local regulatory requirements, and legal requirements.

The Investigator must ensure that each study subject is fully informed about the nature and objectives of the study and possible risks associated with participation and must ensure that the subject has been informed of his/her rights to privacy. The Investigator will obtain written informed consent from each subject before any study-specific activity is performed and will document in the source documentation that consent was obtained prior to enrollment in the study. The original signed copy of the informed consent form must be maintained by the Investigator and is subject to inspection by the Sponsor, their representatives, auditors, the IRB/IEC, and/or regulatory agencies. A copy of the signed informed consent form will be given to the study subject.

If significant new findings are developed during the course of research which may affect the willingness of subjects to continue study participation, the Sponsor will notify each Investigator of the findings via letter or telephone.

12.4. Subject Confidentiality

To maintain subject privacy, all eCRFs, study medication accountability records, study reports and communications will identify the subject by initials and the assigned subject number. The

Investigator will grant monitor(s) and auditor(s) from the Sponsor or its designee and regulatory authority(ies) access to the subject's original medical records for verification of data gathered on the eCRFs and to audit the data collection process. The subject's confidentiality will be maintained and will not be made publicly available to the extent permitted by the applicable laws and regulations.

12.5. Protocol Compliance

The Investigator will conduct the study in compliance with the protocol provided by the Sponsor, the approval/favorable opinion of the IRB/IEC and the appropriate regulatory authority(ies). Modifications to the protocol should not be made without agreement of both the Investigator and the Sponsor. Changes to the protocol will require written IRB/IEC approval/favorable opinion prior to implementation, except when the modification is needed to eliminate an immediate hazard(s) to subjects. The IRB/IEC may provide, if applicable regulatory authority(ies) permits, expedited review and approval/favorable opinion for minor change(s) in ongoing studies that have the approval/favorable opinion of the IRB/IEC. The Sponsor will submit all protocol modifications to the regulatory authority(ies) in accordance with the governing regulations.

When immediate deviation from the protocol is required to eliminate an immediate hazard(s) to subjects, the Investigator will contact the Sponsor, if circumstances permit, to discuss the planned course of action. Any departures from the protocol must be fully documented in the eCRF and source documentation and reported to the appropriate study monitor or staff in a timely fashion.

13. COMPENSATION, INSURANCE, AND INDEMNITY

The Sponsor has retained an insurance policy covering, in its terms and provisions, its legal liability for injuries caused to participating persons and arising out of this research performed strictly in accordance with the scientific protocol as well as with applicable law and professional standards. The subject will be appropriately treated or compensated, or both, for any health or other problems arising from participation in this study.

14. PUBLICATION POLICY

All information provided regarding the study, as well as all information collected/documented during the course of the study, will be regarded as confidential. The Investigator agrees not to disclose such information in any way without prior written permission from the Sponsor.

Any publication of the results, either in part or in total, including articles in journals or newspapers, oral presentations, or abstracts, by the Investigator(s) or their representative(s), shall require prior approval, notification, and review within a reasonable time frame by the Sponsor and cannot be made in violation of the Sponsor's confidentiality restrictions or to the detriment of the Sponsor's intellectual property rights.

It is anticipated that the results of this study will be presented at scientific meetings and/or published in a peer reviewed scientific or medical journal. A Publications Committee, comprised of the Investigators participating in the study and representatives from the Sponsor, as appropriate, will be formed to oversee the publication of the study results, which will reflect the experience of all participating study centers. Subsequently, individual Investigators may publish results from the study in compliance with their agreement with the Sponsor. A pre-publication manuscript is to be provided to the Sponsor at least 60 days prior to the submission of the manuscript to a publisher. Similarly, the Sponsor will provide any company prepared manuscript to the Investigators for review at least 30 days prior to submission to a publisher.

15. CHANGES TO PROTOCOL

Amendment 01 (Version 2.0)

1. Modified objectives to include liver fat content and body composition
2. Modified endpoints to include liver fat content by MRI-PDFF and body composition by MRI
3. Eligibility criteria modified to clarify that urine and not serum pregnancy testing will be used to exclude pregnancy in women of child-bearing potential
4. Schedule off Assessments formatting adjusted to distinguish between assessments conducted for ALT-801-105 and current study ALT-801-106 on the days that overlap for each study (Day 78/screening and Day 85/Day 1).
5. Removed calcitonin from Day 78 assessment on Schedule of Assessments Table
6. Added MRI-PDFF, body composition by MRI and cT1 imaging (for those who previously underwent cT1 imaging in ALT-801-105) to Day 85 visit on Schedule of Assessments Table
7. Modified exclusion criteria to:
    a. Indicate that calcitonin levels would be checks at Week 7 visit of Study ALT-801-105
    b. Correct a typo to sub-bullet excluding use of other GLP-1 agonists or other injectable diabetes treatment
    c. Clarify that receipt or donation of blood products will only be exclusionary if they occur during prior study ALT-801-105
8. Harmonized language in Study Summary and Section 10.2 with intent of protocol and as specified elsewhere, which is to maintain study blinding from Study ALT-801-105.

Amendment 02 (Version 3.0)

1. Clarified that subjects had to meet the exclusion criteria for Study ALT-801-105 at the time of entry into that study.
2. Revised the exclusion criteria for glucose control to exclude subjects only if they met rescue criteria in Study ALT-801-105.
3. Deletion of the exclusion criterion for elevated triglycerides at the time of entry into this study.
4. Clarified that an asymptomatic elevation in amylase or lipase will not be considered an AE.
5. Clarified that an interim analysis will be conducted after all subjects complete 12 weeks of treatment.
6. Minor editorial changes for clarification and consistency.

Amendment 03 (Version 4.0)

1. Inclusion criterion clarified that if one dose of IP is missed in Study ALT-801-105, the subject should still be deemed eligible for participation in this study.

2. The number of study participants was increased to approximately 90.

3. Edits to language to clarify timing of study Day -7 and Day 1 for Study ALT-801-106 coincide with study Day 78 and Day 85 for Study ALT-801-105.

4. Edits made to clarify that subjects will receive the same treatment during Study ALT-801-106 as they received in Study ALT-801-105 and this study will evaluate the effects of 24 weeks of IP (ALT-801 vs placebo).

5. Exclusion criteria clarified that for subjects not meeting the vital signs criteria on Day 78, blood pressure may be reevaluated on Day 85 to establish eligibility at that discretion of the Investigator and Medical Monitor.

16. REFERENCES

US Department for Health and Human Services, Common Terminology Criteria for Adverse Events (CTCAE), Version 5.0, November 27, 2017, https://ctep.cancer.gov/protocolDevelopment/electronic_applications/docs/CTCAE_v5_Quick_Reference_5x7.pdf

17. APPENDICES

APPENDIX 1. SAFETY LABORATORY TESTS

Chemistry	Hematology (CBC) and Coagulation	Dipstick Urinalysis [a] (UA)
Albumin	Hemoglobin	Color and appearance
Alanine aminotransferase (ALT)	Hematocrit	pH and specific gravity
Alkaline phosphatase (ALP)	Mean corpuscular hemoglobin (MCH)	Bilirubin
Amylase	Mean corpuscular hemoglobin concentration (MCHC)	Blood
Aspartate aminotransferase (AST)	Mean corpuscular volume (MCV)	Glucose
Bicarbonate	Platelet count	Ketones
Calcium	Red blood cell count	Leukocytes
Chloride	White blood cell count	Nitrates
Creatinine	White blood cell differential<br>- Eosinophils (% & absolute)<br>- Basophils (% & absolute)<br>- Neutrophils (% &absolute)<br>Monocytes (% & absolute)	Protein
Creatinine Kinase (CK)	International normalized ratio (INR)	Urobilinogen
Direct bilirubin (only if total is elevated)		
Estimated glomerular filtration rate (eGFR)		
Gamma glutamyl transferase (GGT)		
Glucose		
Lipase		
Phosphate		
Potassium		
Sodium		
Total bilirubin		
Total protein		
Blood urea nitrogen (BUN)		

APPENDIX 2. ALGORITHM FOR MONITORING ABNORMAL LIVER FUNCTION TESTS

Subjects with Normal Liver Transaminases and Bilirubin at Baseline of Study ALT-801-105

Drug-induced liver injury monitoring in subjects with normal liver transaminases and bilirubin at baseline should be performed throughout the study according to the procedures summarized below.

- If subjects with normal baseline liver indices develop elevations in ALT of >5 × ULN during the study, repeat testing should be performed within 2 - 5 days from receipt of results.
  - If there are persistent elevations (ALT > 5 x ULN or TBL > 2 × ULN) upon repeat testing, close observation (testing and physical examination 2 to 3 times per week) should be implemented. An important purpose of the close observation is to gather additional clinical information to seek other possible causes of the observed liver test abnormalities, such as one of the following: acute viral hepatitis, alcoholic and autoimmune hepatitis, hepatobiliary disorders, cardiovascular causes, or concomitant treatments. Discontinuation of investigational medicinal product should be considered.
- Study drug should be discontinued, and the subject should be followed until resolution of signs or symptoms, in the following situations:
  - ALT >8 × ULN
  - ALT >5 × ULN and TBL >2 × ULN
  - ALT >5 × ULN with the appearance of fatigue, nausea, vomiting, right upper quadrant pain or tenderness, fever, rash, and/or eosinophilia (>5%) as assessed by the PI to indicate hepatic injury

For any subjects who present with a constellation of syndromes indicative of liver disease (ie, fatigue, nausea, vomiting, right upper quadrant pain or tenderness, fever, rash, and/or eosinophilia [> 5%]), perform liver function tests to determine if liver disease is worsening.

Re-initiation of investigational medicinal product may be considered after consultation with the Medical Monitor.

Subjects with Elevations in Liver Transaminases or Bilirubin at Baseline of Study ALT-801-105

Drug-induced liver injury monitoring in subjects with elevations in liver transaminases or bilirubin at baseline should be performed throughout the study according to the procedures summarized below.

- If subjects with abnormal baseline liver indices develop elevations of ALT >3 × baseline or ≥300 U/L (whichever occurs first) during the study, repeat testing should be performed within 2 - 5 days.

  - If there are persistent elevations (ALT >3 × baseline, or ≥300 U/L) upon repeat testing, then close observation (testing and physical examination 2 to 3 times per week) should be implemented, and discontinuation of study drug should be considered.
- Discontinue the study drug if any of the following occur:
  - ALT increases to >5 × baseline measurements or ≥400 U/L (whichever occurs first).
  - ALT increase >3 × baseline measurements or ≥300 U/L (whichever occurs first) AND the increase is accompanied by a concomitant increase in TBL to > 2 × ULN.
  - ALT increase >3 × baseline measurements or ≥300 U/L (whichever occurs first) with the appearance of fatigue, nausea, vomiting, right upper quadrant pain or tenderness, fever, rash, and/or eosinophilia (> 5%)

For any subjects who present with a constellation of syndromes indicative of liver disease (ie, fatigue, nausea, vomiting, right upper quadrant pain or tenderness, fever, rash, and/or eosinophilia [> 5%]), perform liver function tests to determine if liver disease is worsening.

Re-initiation of investigational medicinal product may be considered after consultation with the Medical Monitor.

Close Observation for Suspected Drug-Induced Liver Injury

For all subjects, close observation for suspected drug-induced liver injury includes the following within 72 hours of suspected drug-induced liver injury:

- Repeating liver enzyme (ALT, AST, and alkaline phosphatase) and serum bilirubin tests 2 or 3 times weekly.
- The frequency of repeat testing can decrease to once a week or less if abnormalities stabilize or the study drug has been discontinued and the subject is asymptomatic.
- Obtaining a more detailed history of symptoms and prior or concurrent diseases.
- Obtaining a history of concomitant drug use (including nonprescription medications and herbal and dietary supplement preparations), alcohol use, recreational drug use, and special diets.
- Ruling out acute viral hepatitis types A, B, C, D, and E; autoimmune or alcoholic hepatitis; hypoxic/ischemic hepatopathy; and biliary tract disease.
- Obtaining a history of exposure to environmental chemical agents.
- Obtaining additional tests to evaluate liver function, as appropriate (eg, INR, direct bilirubin).
- Considering gastroenterology or hepatology consultations.

APPENDIX 3. INVESTIGATOR-PROVIDED INSTRUCTIONS TO SUBJECT: IDENTIFICATION AND MANAGEMENT OF HYPOGLYCEMIA

The following discussion is adapted from instructions provided by the American Diabetes Association on Hypoglycemia (Low Blood Glucose) (https://www.diabetes.org/healthy-living/medication-treatments/blood-glucose-testing-and-control/hypoglycemia).

Symptoms

Hypoglycemia has been observed with GLP-1 agonists and could be exacerbated by the glucagon component of ALT-801, because glucagon reduces liver glycogen and hepatic gluconeogenesis. Therefore, in addition to monitoring daily fasting glucose, subjects should be educated on symptoms that can be experienced with hypoglycemia, including the following:

- Feeling shaky
- Being nervous or anxious
- Sweating, chills and clamminess
- Irritability or impatience
- Confusion
- Fast heartbeat
- Feeling lightheaded or dizzy
- Hunger
- Nausea
- Color draining from the skin (pallor)
- Feeling sleepy
- Feeling weak or having no energy
- Blurred/impaired vision
- Tingling or numbness in the lips, tongue or cheeks
- Headaches
- Coordination problems, clumsiness
- Nightmares or crying out during sleep
- Seizures

Management

If subjects experience symptoms suggestive of hypoglycemia, they should measure their glucose level with their glucometer. For a blood glucose of < 70 mg/dL, they should eat or drink at least 15 grams of carbohydrate and retest their blood glucose level in 15 minutes. They should repeat this process until their blood glucose has normalized (ie, a value of > 70 mg/dL). Once their hypoglycemia has resolved, **they should contact their study site and record the event in their hypoglycemia logs.**

Examples of food containing approximately 15 grams of carbohydrate:

- 4 ounces (1/2 cup) of juice or regular soda (not diet)
- 1 tablespoon of sugar, honey, or corn syrup
- Hard candies, jellybeans or gumdrops—see food label for how many to consume

Information to record in the hypoglycemia logs:

- Date, time, and glucose value for each test performed
- Associated symptoms
- Fasting or non-fasting
- Interventions taken

APPENDIX 4. RESCUE THERAPY FOR PERSISTENT HYPERGLYCEMIA

An additional therapeutic intervention should be considered in subjects if average fasting glucose level is over 180 mg/dL in non-diabetic subjects and over 240 mg/dL (13.3 mmol/L) in diabetic subjects and occurs over at least a 2-week period. To make this determination, at least 4 values/week must be available, with at least 1 confirmed by a laboratory measurement).

If this condition is met, then subjects may begin treatment with or add another antihyperglycemic agent as determined by their physician. If treatment with antihyperglycemic agents is initiated or augmented, then study drug should be permanently discontinued. Subjects may remain in the study for safety follow-up, but the date at which they begin rescue therapy will be the last date for collection of efficacy measures.
