# Supplementary material for: Safety and efficacy of 24 weeks of pemvidutide in metabolic dysfunction-associated steatotic liver disease: A randomized, controlled clinical trial
Source: JHEP Rep. 2025 Jun 18;7(11):101483. doi: 10.1016/j.jhepr.2025.101483 (PMC12529369; doi:10.1016/j.jhepr.2025.101483)
Supplement: Multimedia component 4 [file mmc4.zip › ALT-801-106_SAP Clean V2.pdf]

Statistical Analysis Plan

A 12-Week Extension Study of ALT-801 in Diabetic and Non-Diabetic Overweight and Obese Subjects with Non-alcoholic Fatty Liver Disease

Protocol Number: ALT-801-106

SAP Version and date: *Final 1.0 21-November-2022*

STUDY DRUG:
ALT-801

PREPARED FOR:

Altimmune, Inc.

910 Clopper Road, Suite 201 S
Gaithersburg, MD 20878 USA

PREPARED BY:

Allucent
2000 Centregreen Way Suite 300
Cary, North Carolina 27513

Approval Signature Page

Allucent (formerly CATO SMS)

Document Author: Beatrice Vlas-Irimia<br>Senior Biostatistician II	Date
Document Reviewer: Adrienne Groulx MS<br>Director, Biostatistics	Date

Altimmune, Inc.

Jay Yang, Ph.D<br>Vice President of Biostatistics and Data Management<br>,	Date
Scott Harris, MD<br>Chief Medical Officer	Date

Revision History

SAP Revision Chronology:
Not Applicable

Contents

1 Introduction 9
1.1 Changes to the Protocol Planned Analysis 9
2 Study Objectives and Endpoints 10
3 Study Design 12
3.1 General Description 12
3.2 Randomization and Blinding 12
3.3 Sample Size 13
3.4 Study Committees - Safety Assessment Committee 13
4 Analysis Populations 14
4.1 Enrolled Population 14
4.2 Safety Population 14
4.3 modified Intent-to-Treat (mITT) Population 14
4.4 Pharmacodynamic (PD) Population 14
5 General Considerations 15
5.1 General Data Handling 15
5.2 General Definitions 18
5.3 Data Imputation Rules 20
5.3.1 Sensitivity analyses imputations methods 20
5.4 Visit Windows 20
6 Analysis Methods 21
6.1 Study Subject Data 21
6.1.1 Subject Enrollment and Disposition 21
6.1.2 Protocol Deviations 21
6.1.3 Demographic and Extension Baseline Characteristics 22
6.1.4 Medical History 23
6.1.5 Prior and Concomitant Medication 23
6.1.6 Extension Study Drug Exposure and Compliance 24
6.2 Pharmacodynamics 25
6.2.1 Hepatic Fat Fraction and Liver Volume 25
6.2.2 Anthropometric Parameters 27
6.2.3 Lipid Metabolism 29
6.2.4 Metabolic Markers at 24 Weeks for Combined 105/106 31

6.2.5 Inflammatory Markers at 24 Weeks for Combined 105/106 32
6.2.6 Fibrosis Markers at 24 Weeks for Combined 105/106..... 32
6.2.7 Lipotoxicity markers 33
6.2.8 Multiplicity..... 33
6.3 Quality of Life 34
6.3.1 36-Item Short Form Health Survey (SF-36)..... 34
6.3.2 Impact of Weight on Quality of Life-Lite Clinical Trials version (IWQoL-Lite for CT) 35
6.4 Pharmacokinetics..... 36
6.5 Safety..... 37
6.5.1 Adverse Events..... 37
6.5.2 Injection Site Reactions..... 38
6.5.3 Clinical Laboratory Evaluations..... 38
6.5.4 Blood Glucose by Glucometer 39
6.5.5 Vital Signs and RPP 40
6.5.6 Electrocardiogram (ECG)..... 40
6.5.7 Physical Examinations 41
6.5.8 Immunogenicity..... 41
6.6 Timing of Analyses 41
6.7 Interim Analysis 42
7 APPENDICES..... 43
7.1 Appendix 1: Partial Date Conventions 43
7.2 Appendix 2: Laboratory Parameters..... 45

ABBREVIATIONS

**Abbreviation**	**Definition**
AE	adverse event
ALT	alanine aminotransferase
ANCOVA	analysis of covariance
Apo A, B	apolipoprotein A, B
AST	aspartate aminotransferase
ATC	anatomic therapeutic chemical
BMI	body mass index
BP	bodily pain
CAP	controlled attenuation parameter
cT1	corrected T1 MRI scanning
CTCAE	Common Terminology Criteria for Adverse Events
CV%	coefficient of variation
ECG	electrocardiogram
eCRF	electronic case report form
EDC	electronic data capture
eGFR	estimated glomerular filtration rate
ELF	Enhanced Liver Fibrosis
ET	early termination
FPG	fasting plasma glucose
FSH	follicle stimulating hormone
GH	general health
GI	gastrointestinal
HDL-C	high-density lipoprotein cholesterol
hs-CRP	high sensitivity C-reactive protein
ICH	International Council for Harmonisation
IL-6	interleukin-6
INR	international normalized ratio
IP	investigational product
IRB	institutional review board
ISR	injection site reaction
IWQoL-Lite for CT	Impact of Weight on Quality of Life-Lite Clinical Trials version
IWRS	interactive web response system
LDL-C	low-density lipoprotein cholesterol

Abbreviation	Definition
LOCF	last observation carried forward
LSM	liver stiffness measurement
LSM	least square means
MCP-1	monocyte chemoattractant protein-1
MedDRA	Medical Dictionary for Regulatory Activities
MH	mental health
MRI	magnetic resonance imaging
MRI-PDFF	magnetic resonance imaging derived proton density fat fraction
NAFLD	non-alcoholic fatty liver disease
PAI-1	plasminogen activator inhibitor-1
PD	pharmacodynamic(s)
PF	physical functioning
PK	pharmacokinetic(s)
PRO-C3	N-terminal type III collagen propeptide
QoL	quality of life
QW	once weekly
RF	role function-emotional
RP	role function-physical
RPP	rate-pressure product
SAC	Safety Assessment Committee
SAE	serious adverse event
SAP	statistical analysis plan
SAT	Subcutaneous Adipose Tissue
SC	subcutaneous
SD	standard deviation
SE	Standard error
SF	social functioning
SF-36	Short Form-36
SI	International System of Units
SOC	system organ class
SOP	standard operating procedure
TC	total cholesterol
TG	triglycerides
TNF	tumor necrosis factor

Abbreviation	Definition
TRMV	thigh-region muscle volume
ULN	upper limit of normal
US	United States
VAT	Visceral Adipose Tissue
VCTE	Vibration-Controlled Transient Elastography
VT	vitality
WHO	World Health Organization
WHODD	World Health Organization Drug Dictionary

1 INTRODUCTION

The statistical analysis plan (SAP) details the planned statistical analysis methods required to address the study objectives as described in Altimmune's protocol ALT-801-106.

This extension study is designed to allow for an additional 12 weeks of treatment with investigational product (IP; ALT-801 or placebo) for subjects who complete Study ALT-801-105.

This SAP should be read in conjunction with the study protocol, electronic case report form (eCRF), and any other applicable study documents.

This version of the SAP is based on the protocol ALT-801-106, Amendment 03 (Version 4.0) dated 06 May 2022 and eCRF version 4.0 23 June 2022 and is an extension to the ALT-801-105 SAP Version 1.0 dated 29 July 2022. Changes to these documents may result in subsequent changes to the SAP. The final, sponsor-approved version of the SAP must occur prior to database lock.

1.1 Changes to the Protocol Planned Analysis

The changes incorporated in this document will align with the some of the changes implemented in ALT-801-105 SAP version 1.0 dated 29 July 2022.

The analysis will add one additional study populations:

- modified Intent -to-Treat (mITT) defined as all randomized subjects who receive at least 1 dose of study medication (for MRI and lipids parameters)

Magnetic resonance imaging derived proton density fat fraction (MRI-PDFF) assessment of hepatic fat fraction (percent), liver volume (L) (liters), corrected T1 MRI scanning (cT1), anthropometric parameters (including body weight (kg), BMI (kg/m^2) and body composition by MRI), and lipid parameters will be examined with line plots, with the model-based LSMEAN estimates with 95% confidence intervals by treatment groups.

If conflicts between the SAP and protocol are identified, the language of the SAP will supersede the language of the protocol.

2 STUDY OBJECTIVES AND ENDPOINTS

**Objectives**	**Endpoints**
**Safety**	
• To assess the safety and tolerability of ALT-801 in subjects with NAFLD	• AEs<br>• Vital signs and Rate-Pressure Product (RPP calculated as mean heart rate × mean systolic blood pressure)<br>• Safety labs, including liver function tests and serum glucose<br>• Urinalysis<br>• Physical examination<br>• Injection site reactions<br>• Immunogenicity (neutralizing antibodies)
**Pharmacodynamic**	
• To evaluate the effects of ALT-801 on:<br>hepatic fat fraction and liver volume (L) by MRI-PDFF	• Changes compared to baselines in:<br>• Liver fat content<br>• Hepatic fat fraction by MRI-PDFF
• To evaluate the effects of ALT-801 on:<br>anthropometric parameters	• Changes compared to baselines in:<br>• Body weight<br>• Waist circumference<br>• Body composition by MRI scanning
• To evaluate the effects of ALT-801 on: lipid metabolism	• Changes compared to baselines in:<br>• Total cholesterol (TC)<br>• Low density lipoprotein cholesterol (LDL-C)<br>• High-density lipoprotein cholesterol (HDL-C)<br>• Apolipoprotein A (Apo A) and B (Apo B)<br>• Lipoprotein(a)<br>• Triglycerides (TG)
• To evaluate the effects of ALT-801 on:<br>metabolic markers	• Changes compared to baselines in:<br>• Hemoglobin A1c (HbA1c)<br>• Adiponectin<br>• Leptin
• To evaluate the effects of ALT-801 on:<br>inflammatory markers	• Changes compared to baselines in:<br>• Tumor necrosis factor (TNF)<br>• High-sensitivity C-reactive protein (hs-CRP)<br>• Monocyte chemoattractant protein-1 (MCP-1)<br>• Interleukin-6 (IL-6)<br>Plasminogen activator inhibitor-1 (PAI-1)

• To evaluate the effects of ALT-801 on: fibrosis markers	• Changes compared to baselines in:<br>• N-terminal type III collagen propeptide (PRO-C3)<br>• Enhanced Liver Fibrosis (ELF) test<br>• Fibroscan<br>• Liver inflammatory-fibrotic activity by corrected T1 (cT1) MRI scanning
• To evaluate the effects of ALT-801 on: lipotoxicity markers	• Specific lipids (to be analyzed will be described in a separate analysis plan)
**Quality of Life**	
• To evaluate the effects of ALT-801 on quality of life (QoL) using established QoL instruments.	• Changes in Short Form-36 (SF-36) and Impact of Weight on Quality of Life-Lite Clinical Trials version (IWQoL-Lite for CT) compared to baseline

3 STUDY DESIGN

3.1 General Description

This extension study is designed to allow for an additional 12 weeks of treatment with investigational product (IP; ALT-801 or placebo) for subjects who complete Study ALT-801-105 in order to assess the safety and effects of 24 weeks of treatment with ALT-801 compared to placebo on anthropometric parameters, lipid metabolism, and inflammatory, metabolic, fibrosis, and lipotoxicity markers in diabetic and non-diabetic overweight and obese subjects with nonalcoholic fatty liver disease (NAFLD).

Subjects who completed the Day 85 visit with no more than 1 missed dose of IP in Study ALT-801-105 and who continue to meet eligibility criteria will receive 12 additional weeks of the same treatment they received in Study ALT-801-105. Treatment will remain blinded, and neither the investigator nor subject will be aware of the treatment being received.

Informed consent will be obtained at the screening visit for Study ALT-801-106 on Day -7 (coincides with the Day 78 visit of Study ALT-801-105) and the first dose of IP will be administered on Day 1 (coincides with the Day 85 visit of Study ALT-801-105), as indicated in protocol Table 1. Subsequent visits will be conducted at the clinic, home, or work through the Day 85 or early termination visit of this study. Subjects will return for a safety follow-up visit on Day 110.

Study medication will be administered weekly for 12 weeks. Subjects who received active treatment with ALT-801 in Study ALT-801-105 will continue to receive ALT-801 at the same dose; those that received placebo in Study ALT-801-105 will continue to receive placebo. Thus, treatment groups in this extension study are as follows:

- ALT-801 1.2 mg SC once weekly for 12 weeks
- ALT-801 1.8 mg SC once weekly for 12 weeks
- ALT-801 2.4 mg SC once weekly for 12 weeks
- Placebo SC once weekly for 12 weeks

Each dose of ALT-801 or placebo will be administered as an SC injection in the abdominal region by appropriately trained clinical staff members. The volume of administration will be based on the assigned dose and a concentration of 2.5 mg/mL for the final drug product

Subjects will receive the first dose of study medication on Day 1 (Day 85 ALT-801-105). Subsequent visits will be conducted weekly, at the clinic, home or work, through Day 85/ET or early termination of ALT-801-106.

Subjects will return for a safety Follow-Up visit on Day 110.

3.2 Randomization and Blinding

There is no randomization in this extension study. The treatment a subject receives in this study will be dependent on the treatment group from Study ALT-801-105.

The Pharmacy staff will be unblinded for the purpose of final drug preparation. The pharmacist will consult the interactive web response system (IWRS) for dose allocation, which will be determined by the unblinded study statistician. The pharmacy staff will prepare each dose in compliance with the randomization list.

Knowledge of the randomization list will be limited to the persons responsible for creation of the randomization list, pharmacy staff who prepare the study medications, and any unblinded study monitors or auditors, until all data has been entered in the eCRF, quality control and verification of the eCRF and assignment of subjects to the analysis populations has been completed, the database has been locked, and the study formally unblinded.

3.3 Sample Size

Up to approximately 90 overweight and obese diabetic and nondiabetic subjects with NAFLD who completed the Day 85 visit of Study ALT-801-105 are planned for enrollment in this study.

As this is an extension study being offered to participants of Study ALT-801-105, no sample size calculation is applicable.

3.4 Study Committees - Safety Assessment Committee

The Safety Assessment Committee (SAC) will conduct regular blinded reviews of all AEs and monitor trends in laboratory abnormalities across this study and pooled aggregate data of all studies involving the use of ALT-801. The responsibilities of the SAC are delineated in the Sponsor's Standard Operating Procedure (SOP) REG-002. SAC analyses are not covered in this analysis plan.

4 ANALYSIS POPULATIONS

4.1 Enrolled Population

Defined as all subjects who sign informed consent for participating in extension protocol ALT-801-106.

The Enrolled Population will be used for summaries of subject disposition, and protocol deviations.

4.2 Safety Population

Defined as all randomized subjects who receive at least one dose of study medication in the extension ALT-801-106 protocol.

4.3 modified Intent-to-Treat (mITT) Population

Defined as all randomized subjects who receive at least 1 dose of study medication in the extension ALT-801-106 protocol. The mITT Population will be the primary population for in MRI-PDFF parameters, anthropometric parameters and lipid metabolism parameters and will summarize subjects based on actual treatment received.

4.4 Pharmacodynamic (PD) Population

Defined as all randomized subjects who receive at least one dose of study medication and who have results from baseline and at least one post-baseline PD assessment.

For the PD analysis population, two sets of baseline comparisons are planned:

1) Comparisons to the baseline of Study ALT-801-105; The population will reference the data presented as Combined 105/106

2) Comparisons to the baseline for this extension study ALT-801-106. The population will reference the data presented as Extension

If mITT population is identical with PD population, the analysis will be reported for the mITT population.

In case PD population is significantly lower in sample size than the mITT population, the analysis will be reported for both populations. This will be decided post-hoc on a table-by-table basis among PD endpoints: MRI-PDFF parameters, anthropometric parameters, and lipid metabolism parameters.

5 GENERAL CONSIDERATIONS

5.1 General Data Handling

All analyses will be conducted based on SAS 9.4 or higher. Mock shells for the summary tables, figures, and data listings will be available separately.

Study data will be recorded in eCRFs via IBM Clinical Development for all enrolled. The EDC vendor for this study is MMS Holdings Inc., and data collected will be provided in SAS 9.4 format.

The pharmacodynamic data (metabolic markers, inflammatory markers, fibrosis markers, lipids markers) will be provided by LabConnect in CSV format.

The Safety Lab (hematology, chemistry, lipids, calcitonin, PT/INR, pregnancy test (serum), FSH, urinalysis,) will be provided by LabConnect in CSV format.

The Immunogenicity (neutralizing antibodies) data will be collected in this study and provided by LabCorp as detailed in a separate analysis plan.

The MRI (Liver fat by MRI-PDFF (percent), liver volume (L) (liters) and body composition SAT, VAT, MMRV) data will be provided by Antaros Medical in ASCII format.

cT1 parameters will be provided Perspectum.

Analysis tables will be presented by assigned treatment group using the following groupings of subjects:

- ALT-801 1.2 mg
- ALT-801 1.8 mg
- ALT-801 2.4 mg
- Placebo
- ALT-801 Overall (demographics and safety only)

Comparisons to the baseline of Study ALT-801-105- will be conducted for the mITT population endpoints (and ***if warranted*** by a significantly lower sample size for the PD population on same PD endpoints), *where noted in the relevant sections*.

- Hepatic Fat Fraction and Liver Volume
- Anthropometric Parameters (including sensitivity analyses)
- Weight Loss Responder Analysis
- Lipid Metabolism Parameters
- Metabolic Markers
- Proportion of Subjects with No Diabetes, Pre-Diabetes and Diabetes at 12/24 Weeks
- Inflammatory Markers

Fibrosis Markers Comparisons to the baseline of this study (referenced as extension baseline) will be conducted for the safety parameters analyses.

Three sets of tables will be provided by Diabetic Status [ie. All data, Presence of Diabetes strata (Diabetic), Absence of Diabetes strata (Non-diabetic)] for these selected parameters, *where noted in the relevant sections*

- Anthropometric Parameters- sensitivity analyses
- Summary of Treatment-Emergent Adverse Events Safety
- TEAEs by System Organ Class and Preferred Term
- Clinical Chemistry – Observed Results and Change from Extension Baseline (Fasting Glucose only)
- Clinical Chemistry – Shifts from Extension Baseline to Each Visit (Fasting Glucose only)
- Fasting Plasma Glucose by Glucometer – Observed Results and Change from Extension Baseline

For the comparisons to the baseline of Study ALT-801-105, integrated analyses will require the re-mapping of the study day visits for the extension part of the study, with the visits from main protocol ALT-801-105 remaining unchanged. For extension analyses, Day 85 visit of Study ALT-801-105 is mapped to Day 1 of this study ALT-801-106, and consequently all visits' days will be labelled to reflect the planned visit as Day 1 + 84 days (for all visits performed up to and including Study Day 85 of this study ALT-801-106).

For the comparisons to the extension baseline for this study, visits will be reported as originally collected with no additional label changed.

The table below reflects the reporting for both types of analyses:

**Combined 105/106 Analysis Visit Label**		**Extension 106 Analysis Visit Label**	
**105 Name**	**Mapped Name**	**105 Name**	**Mapped Name**
Day xx (1 to 78)	Day xx (1 to 78)- no change		
Day 85 [1]	Day 85	Day 85 Via 105 [1]	Day 1
**106 Name**		**106 Name**	
Day 1 [2]	Day 85	Day 1	Day 1- no change
Day xx (from 8 to 78)	Day xx +84 (92 to 162)	Day xx (8 to 78)	Day xx (8 to 78) - no change
Day 85	Day 169	Day 85	Day 85- no change

[1] applies to lab and vital sign datasets and analyses
[2] applies to exposure and dosing datasets and analyses

In the analysis description the mapped names will be referenced as timepoints in the model.

Diabetes status at baseline will be used for Combined 105 /106 analyses and extension analyses.

All data in the database will be presented in by-subject data listings. Unless otherwise stated, all listings will be sorted by treatment group, center ID, subject number, and assessment date (and time, if available).

Unless otherwise stated, continuous data will be summarized by treatment group based on n, mean, median, standard deviation (SD), minimum value, and maximum value.

Unless otherwise stated, categorical data will be summarized by treatment group using frequency counts and percentages. Where applicable, 95% confidence intervals (CIs) will be provided. Unless otherwise stated, the denominator of percentages will be the number of participants with non-missing data in the defined population/ treatment group.

- The number of missing values will be presented as a separate category with no percentage, but only if one or more subjects are missing data.
- Counts of zero will be presented without percentages.

Relative to the number of digits after the decimal in the raw data, summary statistics will have the following number of digits after the decimal:

- Minimum and Maximum: same number of significant digits as the raw data
- Mean, Median: one additional significant digit than the Minimum and Maximum
- SD, CV%, two additional significant digits than the Minimum and Maximum
- Percentages <100% will be reported to one decimal place and percentages of 100% will be reported with no decimal place.
- If applicable P-values will be reported to four decimal places. If the value is below 0.0001 it will be noted as < 0.0001; if the value is above 0.9999 it will be noted as > 0.9999.
- Summary statistics will not exceed four digits after the decimal. Some laboratory parameters or other data may require judicious deviation from this rule in accordance with the raw data.

Unless otherwise noted, statistical inference will be based on a two-sided 5% significance level (i.e., 95% confidence intervals will be produced).

All data up to the time of study completion/withdrawal from study will be included in the analysis, regardless of duration of treatment. Numbering for data displays will be based on ICH E3.

5.2 General Definitions

Variable	Definition
Screening date	Defined as the eCRF provided date on which a subject was screened for trial entry.
Main Treatment start date	Defined as the date of first dose of study drug under protocol ALT-801-105 (Day 1).
Main Treatment end date	Defined as the date of last dose of study drug under protocol ALT-801-105.
Extension Treatment start date	Defined as the date of first dose of study drug under protocol ALT-801-106 (extension Day 1)
Extension Treatment end date	Defined as the date of last dose of study drug under protocol ALT-801-106.
Study Day	Based on treatment start date as a reference date.<br>• Study Day = date of interest – reference date + 1, when the date of interest >= reference date;<br>• otherwise, Study Day = date of interest – reference date.<br>Study day will either have a negative value if collected before dosing, or a positive value if collected on or after the day of drug dosing; there will be no study day zero.<br>Note: if either day is missing, reference date calculations will not be performed. Should imputation be performed, then Study Day may be computed, where appropriate as identified in section 5.3
Age (yr)	Age is auto calculated and collected in the EDC system and uses the informed consent date as its reference date.
Main Day 1	Defined as day of first dose of study treatment in Study ALT-801-105
Extension Day 1	Defined as day of first dose of study treatment in this study (coincides with the Day 85 visit of Study ALT-801-105)
Main Baseline	Defined as the last non-missing value collected prior to receiving the first dose of study treatment of Study ALT-801-105 (based on date and time of administration).
Extension Baseline	Defined as last non-missing value collected prior to receiving the first dose of study treatment on Day 1 of this study or as otherwise indicated nominal Day 85 value from ALT-801-105 for MRI-PDFF, Fibrosis Markers including Fibroscan and CT1 parameters, regardless of timing to Extension Day 1.
Post baseline	Defined as values collected after receipt of the first dose of study treatment in Study ALT-801-105 (based on date and time of administration)
Post- Extension Baseline	Defined as values collected after receipt of the first dose of study treatment in Study ALT-801-106 (based on date and time of

	administration) or as otherwise indicated nominal visit value for MRI-PDFF, Fibroscan and CT1 parameters,
Change from Baseline	Defined as: Post-baseline value – Baseline value
Change from Extension Baseline	Defined as: Extension Post-baseline value – Extension Baseline value
Percent Change from Baseline	Defined as: (Post-baseline value – Baseline value)/Baseline value x 100.<br>Note: To compute percent change from baseline, the baseline value cannot be equal to zero.
Percent Change from Extension Baseline	Defined as: (Extension Post-baseline value – Extension Baseline value)/ Extension Baseline value x 100.<br>Note: To compute percent change from baseline, the baseline value cannot be equal to zero.
Most Extreme Change	The most extreme change will be based on be the maximum grade change. The maximum grade change is defined as the largest change from baseline, in either direction, positive or negative.<br>This calculation will consider assessments collected during the on-therapy period and assessed with CTCAE grading, scheduled or unscheduled. Defined for this study only
Minimum Extreme Change	Defined as: the lowest post-baseline value. All post-baseline assessments were considered, scheduled and unscheduled.
Minimum Extreme Change Extension	Defined as: the lowest extension post-baseline value. All extension post-baseline assessments were considered, scheduled and unscheduled.
Maximum Extreme Change	Defined as: the highest post-baseline value; All post-baseline assessments were considered, scheduled and unscheduled.
Maximum Extreme Change Extension	Defined as: the highest extension post-baseline value; All extension post-baseline assessments were considered, scheduled and unscheduled.
Duration on Study (in days)	Defined as the End of this study date – randomization date (study ALT-801-105) + 1
Duration on Extension Study (in days)	Defined as the End of this study date – first dosing date in this study + 1 (based on date and time of drug administration)
Duration of Exposure (days)	Defined as Treatment end date– Treatment start date + 1(based on date and time of administration
Duration of Extension Exposure (days)	Defined as Extension Treatment end date– Extension Treatment start date in this study + 1(based on date and time of drug administration)
Duration of Adverse Event (in days)	• Defined for full start date/time and full stop date/time for this study

	• Stop date of event – start date of event + 1 if time is not collected.<br>• (Stop date/time of event – start date/time of event)/24, if time is collected.
Reporting in Months	Divide number of days by 30.4375
Reporting in Years	Divide number of days by 365.25
Reporting in Weeks	Divide number of days by 7

5.3 Data Imputation Rules

All attempts will be made by the Data Management team to ensure completeness of data. Generally, missing data will not be imputed, and will be presented as collected in the study database.

In cases where adverse event (AE) or medication dates are missing, the imputation methods described in Appendix 1 will be used to determine flags for treatment-emergent events, and concomitant medications.

Other missing data methods may be proposed within the respective analysis section, as needed.

For interim analyses reporting purposes or ongoing reporting needs, when applicable, study end dates will be imputed as the earliest of the data cutoff date, date of death, or date of study withdrawal.

For MRI-PDFF, anthropometric parameters and lipid metabolism analyses using mITT population (and PD population, if deemed necessary by Sponsor), missing endpoints will use any imputations that were performed during the ALT-801-105 data collection schedule for final analysis. Results from this study will be added as a timepoint to these data, with no further imputation performed.

5.3.1 Sensitivity analyses imputations methods

For anthropometric parameters and lipid metabolism analyses using the mITT population (and PD population, if deemed necessary by Sponsor) missing endpoints may be imputed using the methods described in the ALT-801-105 SAP, as applicable.

5.4 Visit Windows

For safety analyses, the data will be summarized using the recorded nominal visit values.

For multiple laboratory collection dates within a nominal visit window, the earliest assessment occurring within a visit window will be used for that visit.

Unscheduled visits will not be summarized in tables but will be presented in data listings and included for minimum, maximum and most extreme change calculations.

6 ANALYSIS METHODS

6.1 Study Subject Data

6.1.1 Subject Enrollment and Disposition

A summary of subject enrollment will be presented by treatment group for pooled data for subjects consenting to ALT-801-106 protocol.

Disposition data will be summarized for all enrolled subjects in the extension study ALT-801-106. The number all enrolled subjects, subjects enrolled but not treated, number of subjects in each analysis population, number of subjects completing the extension study, number of subjects who discontinued treatment, including discontinuation of treatment, due to the use of rescue medication and the number of subjects who discontinued the extension study, including reasons for discontinuation of the study, will be summarized.

A by-subject listing of enrollment and disposition data, Informed consent date, under which protocol version, treatment group, reference extension Day 1 date, completion date, discontinuation date, including reason for discontinuation (if applicable), analysis population flags, will be presented for all subjects in the extension study. Subjects enrolled but not treated will be identified in a separate listing.

The following reasons for treatment and study discontinuations will be summarized as reported in eCRF

- Adverse event (or SAE)
- Protocol deviation,
- Lost to follow-up
- Voluntary withdrawal of consent
- Discretion of Investigator
- Pregnant or breast feeding
- Study discontinuation by Sponsor
- Other

As indicated in protocol Appendix 4, if treatment with another antihyperglycemic agent is instituted, then study drug should be permanently discontinued while subject may remain in the study for safety follow-up, however the date at which they begin rescue therapy will be the last date for collection of efficacy measures. Therefore, summary of subject disposition is to include the number of subjects who permanently discontinued study drug due to administration of rescue medication.

A by-subject listing of inclusion/exclusion criteria will also be produced for all subjects.

6.1.2 Protocol Deviations

Protocol deviations will be listed for all enrolled subjects and will include the following details as reported in the eCRF:

- Category (i.e., “Informed Consent”, “Eligibility (Inclusion/Exclusion Criteria)”, “Study Procedures/Assessments”, “Investigational Product (IP) Administration”, “Other”)
- Subcategory (as per eCRF)
- Date identified
- Deviation date
- Description (text)
- Date IRB informed (if applicable)
- Designation of deviation (i.e., “Important Deviation”, “Not Important Deviation”, “Important Deviation Due to COVID-19 Pandemic”, or “Not Important Deviation Due to COVID-19 Pandemic”)
- Comments, if any

Protocol deviations will be identified and classified as important or not important (violations) before the database is locked.

Protocol deviations will be summarized by treatment group and overall, for all subjects during the course of the Extension study.

6.1.3 Demographic and Extension Baseline Characteristics

Subject demographics and extension baseline characteristics will be summarized for the Safety Population. These data will include:

- Age (years)
- Gender (Male / Female)
- Fertility status (Childbearing potential / Post-menopause / Surgically sterile)
- Ethnicity (Hispanic or Latino / Not Hispanic or Latino)
- Race (American Indian or Alaska Native / Asian / Black or African American / Native Hawaiian or Pacific Islander / White / Other)
- Screening height (cm)
- Screening weight (kg)
- Screening BMI (kg/m^2)
- Screening waist circumference (cm)

A second table will summarize the following study disease extension baseline characteristics:

- Diabetes status (present, absent) at baseline and extension baseline. Note: all by-strata tables will present based on results from baseline, as noted in section 5.1.
- Pre-diabetes (present, absent) (will not include patients with diabetes)
- Metabolic syndrome (present, absent)
- Metformin use. Note: as this use is not explicitly collected on the CRF, it will be flagged programmatically. A confirmatory use of Metformin will require a verbatim term from the ALT-801-106 prior/concomitant medication page to contain “METFORMIN” on a record with a start date occurring prior to the first dose of ALT-801-106.
- Serum Fasting Glucose test (mg/dL)
- HbA1c(%)

- Baseline lipids (total cholesterol (mg/dL), HDL-C (mg/dL), LDL-C (mg/dL), triglycerides (mg/dL))
- Controlled Attenuation Parameter (CAP) via FibroScan® (dB/m)
- Liver Stiffness Measurement (LSM) via FibroScan® at Screening (kPa)
- ALT/AST (IU/mL)
- Diastolic / Systolic Blood Pressure (mm Hg)

Age (years) and BMI(kg/m^2) will be reported as collected in the clinical database.

Demographics and extension baseline characteristics will be summarized by treatment group for pooled data.

A listing of all demographic characteristics, including fertility status for female subjects, will be provided for all subjects in the Safety Population.

6.1.4 Medical History

All medical history data will be presented in data listings for the Safety Population and will include the categorical body system as identified in the eCRF, the verbatim term for the medical history (diagnosis /procedure), and the coded system organ class (SOC) and preferred term (PT) according to the most current Medical Dictionary for Regulatory Activities (MedDRA) version.

6.1.5 Prior and Concomitant Medication

Concomitant medications will be recorded from the time of informed consent to the extension protocol ALT-801-106 through the Day 110 Follow-up Visit and will be coded using the most current WHO Drug Dictionary.

The incidence of medication use will be summarized by WHO Drug Dictionary (WHODD) anatomic therapeutic chemical (ATC) Level 2 classification (i.e., therapeutic main group) and preferred name. A subject will be counted only once at each level of reporting.

Concomitant medication will be reported for data collected during the extension study ALT-801-106.

Prior medications are those which have been identified to have been discontinued prior to the extension study treatment start datetime (Day 85 ALT-801-105 / Day 1 ALT-801-106). Concomitant medications are those which have been identified to have been taken at any point during extension study after treatment start date (Day 85 ALT-801-105 / Day 1 ALT-801-106. Prior and concomitant medication use will be summarized separately and presented by treatment group and Overall for Safety Population.

All prior and concomitant medication data will be listed including the verbatim and preferred drug name and ATC Level 2 for the Safety Population

Missing start and end dates and times will be imputed as described in Appendix 1. Should a missing start date, start time, end date, or end time led to ambiguity in whether a medication is prior or concomitant, the medication will be considered concomitant.

6.1.5.1 Prohibited Medications and Procedures

The medications that are prohibited during the study are described in protocol section 6.7. If reported, prohibited medication will be included in the protocol deviation listing as recorded in eCRF.

6.1.5.2 Rescue Medications for Persistent Hyperglycemia

Rescue therapy with another antihyperglycemic agent for persistent hyperglycemia is allowed in subjects enrolled in the extension study who meet criteria described in protocol Appendix 4.

If treatment with another antihyperglycemic agent is instituted, then study drug should be permanently discontinued. Subjects may remain in the study for safety follow-up, but the date at which they begin rescue therapy will be the last date for collection of efficacy measures.

The number of subjects who permanently discontinued study drug due to administration of rescue medication will be reported in subjects disposition table, and rescue medications for persistent hyperglycemia will be listed separately, including the verbatim and preferred drug name and ATC Level 2 for Safety Population.

6.1.6 Extension Study Drug Exposure and Compliance

Study drug exposure and compliance will be summarized by treatment group, for pooled subjects, for the Safety Population, **during the extension study**.

The **duration of study drug exposure (days)** defined as:

Duration of Study Drug Exposure (days) = Extension Study Drug Stop Date – Extension Study Drug Start Date [Day 1] + 1

will be summarized as a continuous variable.

Per protocol ALT-801-106 dosing for subject is planned as 12 doses (1 dose QW for 12 weeks) as done in protocol ALT-801-105. Study drug exposure will be summarized first as a categorical variable with frequency and percentage of subjects for number of doses administered.

In addition, number of doses taken, number of doses missed (out of the expected 12 doses planned on-study) will be summarized also as a continuous variable.

The **total dose (mg)** will be derived from by summing the doses of all injections received on Extension study as follows:

- ALT-801 1.2 mg once weekly for 12 weeks (ie. total dose planned (mg) = 14.4mg)
- ALT-801 1.8 mg once weekly for 12 weeks (ie. total dose planned (mg) = 21.6mg)
- ALT-801 2.4 mg once weekly for 12 weeks (ie. total dose planned (mg) = 28.8mg)

Mean daily dose for the extension study ALT-801-106 will be calculated as:

Mean daily dose (mg) = Total dose (in mg) / Duration of Extension Study drug exposure (days)

Both total dose and mean daily dose will be summarized descriptively.

For the time subject is on study (including post study drug discontinuation in case rescue medication is administered) the Overall compliance (%) will be determined as:

Overall compliance (%) = (Total dose taken (in mg) / Total dose planned (in mg)) * 100

Overall compliance (%) will be summarized.

For all subjects, listings of drug administration, whether drug was received, reasons subjects not dosed, dosing dates and times, and dosing location will be produced for the Safety Population.

6.2 Pharmacodynamics

MRI-PDFF parameters, anthropometric parameters, lipid metabolism parameters, metabolic markers, fibrosis markers, inflammatory markers analysis Anthropometric Parameters- sensitivity analyses will be reported by diabetes status stratum at baseline and pooled data using mITT Population.

The remaining Pharmacodynamics analysis (MRI-PDFF parameters, lipid metabolism parameters, metabolic markers, fibrosis markers, inflammatory markers) will be performed using mITT population (and PD population if deemed necessary by Sponsor) pooled data.

6.2.1 Hepatic Fat Fraction and Liver Volume

6.2.1.1 Relative and Absolute % Change for at 24 Weeks for Combined 105/106

MRI-PDFF, a quantitative imaging biomarker will assess the hepatic fat fraction over the entire liver during screening, following a minimum 8 hour fast. The following parameters will be measured:

- PDFF Liver fat (%) or hepatic fat fraction
- Liver volume (L)

Liver fat (%) and liver volume (L) will be summarized by treatment group for pooled data for the mITT population (and PD population, if deemed necessary by Sponsor).

Observed liver fat (%), liver volume (L), at baseline, Day 43, Day 85 and Day 169 (ALT-801-105/ALT-801-106), Change from baseline and % Change from baseline will be summarized by treatment group for pooled data with descriptive statistics (sample size [N], arithmetic mean, SD, median, minimum, and maximum).

The absolute and relative Change from baseline in hepatic fat fraction (%) by MRI-PDFF and in liver volume (L) will be compared between ALT-801 and Placebo groups using the analysis of covariance (ANCOVA) at each timepoint.

The model will be fit with MRI-PDFF parameter Change from baseline as dependent variable and treatment group and the stratum (diabetic, non-diabetic) as factors, baseline value as covariate and

the corresponding baseline demographic characteristics (gender, race, BMI) as covariates. The least square means and 95% CI of LSM will be reported for variables analyzed with the ANCOVA model. Tests for statistical difference on the Change from baseline in Liver fat (%) and liver volume (L) between each ALT-801 treatment group and Placebo group will be conducted at each timepoint. The p-values from significance tests will be obtained at a two-sided significance level of 0.05 using Dunnett's test for the comparison of change differences.

MRI-PDFF parameters will be examined by treatment groups with **line plots** representing the ANCOVA model based LSMEAN estimates for change from baseline with 95% confidence intervals. The plot will identify the treatment that the subject was in by color lines (ALT-801 doses vs. Placebo) .

6.2.1.1.1 Sensitivity analyses

Sensitivity analyses **may be performed** for MRI-PDFF parameters liver fat (%), liver volume (L), for the mITT population (and PD population, if deemed necessary by Sponsor).

To evaluates the treatment effect of ALT-801 treatments relative to placebo if all patients had adhered to treatment(compliance) and didn't receive rescue therapy an efficacy estimand may be defined, with resulting missing values (discarded after the use of rescue medication and/or treatment discontinuation, or unobserved) being handled by the MMRM under the assumption of missing at random (MAR). Another sensitivity analysis may use a pattern mixture model (PMM) under the assumption of missing not at random (MNAR).

A mixed-effects model for repeated measures (MMRM) **may also be performed** on the **Change from baseline** in MRI-PDFF parameters liver fat (%), liver volume (L), on mITT population (and PD population, if deemed necessary by Sponsor). The MMRM model will include the fixed effects of treatment doses, timepoint (Day 43, Day 85 and Day 169 (ALT-801-105/ALT-801-106)), and treatment-by-timepoint interaction. The model will include the parameters' baseline value, and baseline BMI as continuous covariates, with diabetes stratum, gender, and race as factors. The model will employ an unstructured within-subject covariance matrix and a restricted maximum likelihood estimation (REML) method. If convergence is not met using the unstructured covariance matrix, a variance components structure will be used.

The MMRM model would be fit under the MAR (ALT-801-105 SAP section 5.3.1.3) and MNAR (ALT-801-105 SAP section 5.3.1.4.) assumptions.

All MRI-PDFF parameter will be listed for the extension part of the study.

6.2.1.2 Proportion of Subjects Achieving 30%, 40% and 50% Relative Reductions in Hepatic Fat Fraction at 24 Weeks for Combined 105/106

The proportion of subjects [n(%)] who achieve a 30%, 40% and 50% relative reductions from baseline in hepatic fat fraction from baseline at each visit, Day 43, Day 85 and Day 169 (ALT-801-105/ALT-801-106) will be summarized and presented by treatment groups for pooled data. 95% CI computed using the Clopper-Pearson method together with P-values for comparing each ALT-801 treatment group to Placebo will be computed using Cochran-Mantel-Haenszel (CMH)

test controlling for stratification factor (diabetic, non-diabetic), at a two-sided significance level of 0.05.

6.2.1.3 Proportions of subjects with normalization of liver fat (%)

The normalization of liver fat (%) is defined as subject having defined a liver fat (%) content below 5% at Day 85 ALT-801-106 visit when compared to Extension Baseline for the PD Population.

The proportion of subjects [n(%)] who achieve normalization by MRI-PDFF at Day 85 ALT-801-106 visit will be summarized and presented by treatment group forpooled data. P-values together with 95% CI computed using the Clopper-Pearson method for comparing each ALT-801 treatment group to Placebo will be computed using Cochrane Mantel Haenszel (CMH) test controlling for stratification factor (diabetic, non-diabetic), at a two-sided significance level of 0.05.

6.2.2 Anthropometric Parameters

The following parameters will be measured:

- VAT (Visceral Adipose Tissue) - liters (L) via MRI-PDFF
- SAT (Subcutaneous Adipose Tissue) - liters (L) via MRI-PDFF
- TRMV (Thigh-region Muscle Volume) - liters (L) via MRI-PDFF
- Waist circumference (cm),
- Weight (kg), and
- BMI (kg/m^2)

Anthropometric parameters and all sensitivity analyses will be presented by treatment group for each stratum (diabetic, non-diabetic) and pooled data (Anthropometric parameters summaries only) for the mITT population (and PD population, if deemed necessary by Sponsor).

6.2.2.1 Body Composition at 24 Weeks for Combined 105/106

Observed values of anthropometric parameters, Body composition by MRI scanning (VAD (L), SAT (L), TRMV (L)), at baseline, Day 43, Day 85 and Day 169 (ALT-801-105/ALT-801-106) with Change from baseline and % Change from baseline will be summarized by treatment group for pooled data with descriptive statistics (sample size [N], arithmetic mean, SD, median, minimum, and maximum).

Weight (kg), Waist circumference (cm) and BMI (kg/m^2) baseline and weekly (i.e., Days 8, 15, 22, 29, 36, 43, 50, 57, 64, 71, 78, 85, 92, 99, 106, 113, 120, 127, 134, 141, 148, 155, 162, 169 (ALT-801-105/ALT-801-106)) observed values together with Change and % Change from baseline will be summarized in the same manner.

The **Change from baseline** in all anthropometric parameters will be compared between each ALT-801 groups and Placebo group using the analysis of covariance (ANCOVA) at each timepoint. The model will be fit with anthropometric parameter **Change from baseline** as dependent variable and treatment group and the stratum (diabetic, non-diabetic) as factors, **baseline** value as covariate and the corresponding **baseline** demographic characteristics (gender, race, BMI) as covariates. The least square means and 95% CI of LSM will be reported for variables analyzed with the ANCOVA model. Tests for statistical difference on the Change from baseline in anthropometric parameters

between each ALT-801 treatment group and Placebo group will be conducted at each timepoint. The p-values from significance tests will be obtained at a two-sided significance level of 0.05 using Dunnett's test for the comparison of change differences.

6.2.2.1.1 Sensitivity analyses

Sensitivity analyses **may be performed** by stratum (diabetic /non-diabetic) and pooled data for Body composition by MRI scanning (VAD (L), SAT (L), TRMV (L)), Waist circumference (cm), Weight (kg), and BMI (kg/m2) for the mITT population (and PD population, if deemed necessary by Sponsor).

To evaluate the treatment effect of ALT-801 treatments relative to placebo irrespective of adherence to investigational product ALT-801 or introduction of rescue, a treatment regimen estimand will be defined, with missing endpoint data imputed using method of multiple imputation (MI) based on placebo arm (ALT-801-105 SAP section 5.3.1.2.). Another sensitivity analysis will use last observation carried forward (LOCF) imputation (ALT-801-105 SAP section 5.3.1.1). The same ANCOVA model comparisons (sections 6.2.2.1) will be performed for data all visits for these two sensitivity analyses.

To evaluates the treatment effect of ALT-801 treatments relative to placebo if all patients had adhered to treatment(compliance) and didn't receive rescue therapy an efficacy estimand may be defined, with resulting missing values (discarded after the use of rescue medication and/or treatment discontinuation, or unobserved) being handled by the MMRM under the assumption of missing at random (MAR). Another sensitivity analysis may use a pattern mixture model (PMM) under the assumption of missing not at random (MNAR).

A mixed-effects model for repeated measures (MMRM) **may also be performed** on the **Change from baseline** in Body composition by MRI scanning VAD (L), SAT (L), TRMV (L), on mITT population (and PD population, if deemed necessary by Sponsor). The MMRM model will include the fixed effects of treatment doses, timepoint (Day 43, Day 85 and Day 169 (ALT-801-105/ALT-801-106)), and treatment-by-timepoint interaction. The model will include the parameters' baseline value, and baseline BMI as continuous covariates, with diabetes stratum, gender, and race as factors. The model will employ an unstructured within-subject covariance matrix and a restricted maximum likelihood estimation (REML) method. If convergence is not met using the unstructured covariance matrix, a variance components structure will be used.

The MMRM model would be fit under the MAR (ALT-801-105 SAP section 5.3.1.3) and MNAR (ALT-801-105 SAP section 5.3.1.4.) assumptions.

A separate mixed-effects model for repeated measures (MMRM) will also be performed on the change from baseline in body Weight (kg), and BMI (kg/m^2) based on mITT population (and PD population, if deemed necessary by Sponsor). The MMRM model will include the fixed effects of treatment doses, timepoint (i.e., Days 8, 15, 22, 29, 36, 43, 50, 57, 64, 71, 78, 85, 92, 99, 106, 113, 120, 127, 134, 141, 148, 155, 162, 169 (ALT-801-105/ALT-801-106)) and treatment-by-timepoint interaction. The model will include the baseline weight /BMI as a continuous covariate. The model will employ an unstructured within-subject covariance matrix and a restricted maximum likelihood

estimation (REML) method. If convergence is not met using the unstructured covariance matrix, a variance components structure will be used.

Anthropometric parameters including VAD (L), SAT (L), TRMV (L), Waist circumference (cm), Weight (kg), and BMI (kg/m^2) will be examined by treatment groups with line plots representing the MMRM-under MAR model based LSMEAN estimates for change from baseline with 95% confidence intervals. The plot will identify the treatment by color lines (ALT-801 doses vs. Placebo) and a different line type (solid, dashed) for each stratum (diabetic, non-diabetic) that the subject was in.

All anthropometric parameters will be listed for the extension part of the study.

6.2.2.2 Weight Loss Responder and Weight Loss Over Time at 24 Weeks for Combined 105/106 - Additional Analysis

The proportion of subjects [n(%)] who achieve a 5%, 10% relative reductions in Weight (kg) from baseline at visits Days 8, 15, 22, 29, 36, 43, 50, 57, 64, 71, 78, 85, 92, 99, 106, 113, 120, 127, 134, 141, 148, 155, 162, 169 (ALT-801-105/ALT-801-106) will be summarized and presented by treatment groups for pooled data. 95% CI computed using the Clopper-Pearson method together with P-values for comparing each ALT-801 treatment group to Placebo will be computed using Cochran-Mantel-Haenszel (CMH) test controlling for stratification factor (diabetic, non-diabetic), at a two-sided significance level of 0.05.

The Weight Loss at 24 Weeks will be examined by treatment groups with line plots representing the LSMEAN estimates with 95% confidence intervals from the MMRM model below. The plot will identify the treatment by color lines (ALT-801 doses vs. Placebo) and a different line type (solid, dashed) for each stratum (diabetic, non-diabetic) that the subject was in.

A mixed-effects model for repeated measures (MMRM) will also be performed on the **% Change from baseline in body weight**. The MMRM model will include the fixed effects of treatment doses, timepoint (i.e. Days 8, 15, 22, 29, 36, 43, 50, 57, 64, 71, 78, 85, 92, 99, 106, 113, 120, 127, 134, 141, 148, 155, 162, 169 (ALT-801-105/ALT-801-106)), and treatment-by-timepoint interaction. The model will employ an unstructured within-subject covariance matrix and a restricted maximum likelihood estimation (REML) method. If convergence is not met using the unstructured covariance matrix, a variance components structure will be used.

6.2.3 Lipid Metabolism

Blood samples for analysis of lipid metabolism will be collected at Screening, Day 1, Day 85 ALT-801-105 / Day 1 ALT-801-106, Day 85 and Day 110/Follow-Up ALT-801-106 and includes the following parameters

- Total cholesterol (TC)
- Low-density lipoprotein cholesterol (LDL-C)
- High-density lipoprotein cholesterol (HDL-C)
- Apolipoprotein A1 (Apo A) and B (Apo B)
- Lipoprotein(a) and Apolipoprotein B/A1 ratio
- Triglycerides (TG)

Lipids parameters will be summarized by treatment group for pooled data for the mITT population (and PD population, if deemed necessary by Sponsor).

6.2.3.1 Lipid Parameters at 24 Weeks for Combined 105/106

Observed values at baseline, Day 85, 169 (ALT-801-105/ALT-801-106) with Change from baseline will be summarized by treatment group pooled data with descriptive statistics (sample size [N], arithmetic mean, SD, median, minimum, and maximum).

The **Change from baseline** in lipids will be compared between each ALT-801 groups and Placebo group using the analysis of covariance (ANCOVA) at each timepoint. The model will be fit with lipid parameter **Change from baseline** as dependent variable and treatment group and the stratum (diabetic, non-diabetic) as factors, baseline value as covariate and the corresponding **baseline** demographic characteristics (gender, race, BMI) as covariates. The least square means and 95% CI of LSM will be reported for variables analyzed with the ANCOVA model. Tests for statistical difference on the Change from baseline in lipids between each ALT-801 treatment group and Placebo group will be conducted at each timepoint. The p-values from significance tests will be obtained at a two-sided significance level of 0.05 using Dunnett's test for the comparison of change differences.

Lipids parameters will be examined by treatment groups with line plots representing the ANCOVA model based LSMEAN estimates for Change from baseline with 95% confidence intervals. The plot will identify the treatment that the subject was in by color lines (ALT-801 doses vs. Placebo

All lipid metabolism parameters will be listed for the extension part of the study.

6.2.3.2 Diabetes Status at 12 Weeks and 24 Weeks

The proportions of subjects with no diabetes, pre-diabetes and diabetes will be summarized in a shift from baseline at Day 85 (12 weeks of treatment) and Day 169 (24 weeks of treatment) for mITT population (and PD population, if deemed necessary by Sponsor).

6.2.3.3 A fasting glucose level of 99 mg/dL or lower is considered normal (no diabetes), 100 to 125 mg/dL indicates pre-diabetes status, and 126 mg/dL or higher indicates diabetes status.Metabolic Syndrome

The proportions of subjects with metabolic syndrome at Day 85 ALT-801-106 (12 weeks of treatment) will be compared to the proportions of subjects at **Extension baseline** for mITT population (and PD population, if deemed necessary by Sponsor).

Metabolic syndrome is defined by the presence of 3 of the 5 characteristics (per the ATP III classification) at timepoint evaluated (Extension Baseline, and Day 85 ALT-801-106):

- Waist circumference > 102 cm in men and 88 cm in women
- Triglycerides ≥ 150 mg/dL or on treatment. The subjects on treatment for triglycerides may have hypertriglyceridemia, hyperlipidemia, or dyslipidemia, or take fibrates, statins, or omega-3 fatty acids
- HDL cholesterol < 40 mg/dL in men and < 50 mg/dL in women

- Systolic blood pressure ≥ 130 mmHg or diastolic blood pressure > 85 mmHg or on treatment. The treated subjects may have hypertension or high blood pressure and may take many classes of antihypertensives and many drugs within those classes such as ACE inhibitors, Ace receptor blockers, Beta blockers, Calcium channel blockers, diuretics (thiazides (hydrochlorothiazide) and loop diuretics (furosemide)), and alpha blockers.
- Fasting glucose ≥ 100 mg/dL based on safety laboratory at baseline.

If a subject has less than 3 of these characteristics at either Screening or baseline or has 3 or more of these characteristics at Screening but less than 3 after 12 weeks of treatment, that subject will not or no longer be considered to have metabolic syndrome.

The proportions of subjects [n(%)] with metabolic syndrome at Extension Baseline and at Day 85/ET ALT-801-106 will be summarized and presented by treatment groups. P-values together with 95% CI computed using the Clopper-Pearson method for comparing each ALT-801 treatment group to Placebo will be computed using Cochrane Mantel Haenszel (CMH) test controlling for stratification factor (diabetic, non-diabetic), at a two-sided significance level of 0.05.

For the subjects without diabetes present at **extension baseline** stratum, only the proportion of subjects with pre-diabetes at Day 85/ET ALT-801-106 will be summarized and presented by treatment groups. The associated two-sided 95% CI for will be computed using exact binomial Clopper-Pearson CI. P-values are from a Fisher's exact test comparing the proportion of subjects with pre-diabetes after 12 weeks of treatment in each ALT-801 treatment group to subjects in the Placebo group.

All Metabolic syndrome defining criteria will be listed for the extension part of the study and include gender and original and standardized units and values.

6.2.4 Metabolic Markers at 24 Weeks for Combined 105/106

Blood will be collected for the assessment of metabolic markers at Screening, Day 1, Day 85 ALT-801-105 / Day 1 ALT-801-105, and Day 85/ET ALT-801-106 and includes the following parameters:

- Hemoglobin A1c (HbA1c) (%)
- Adiponectin (ug/mL)
- Leptin (ng/mL)

Observed values at baseline, Day 85, Day 169 (ALT-801-105/ALT-801-106), with Change from baseline will be summarized by treatment group for pooled data with descriptive statistics (sample size [N], arithmetic mean, SD, median, minimum, and maximum).

The **Change from baseline** metabolic markers will be compared between each ALT-801 groups and Placebo group using the analysis of covariance (ANCOVA) at each timepoint. The model will be fit with metabolic markers **Change from baseline** as dependent variable and treatment group and the stratum (diabetic, non-diabetic) as factors, baseline value as covariate and the corresponding **baseline** demographic characteristics (gender, race, BMI) as covariates. The least square means and 95% CI of LSM will be reported for variables analyzed with the ANCOVA

model. Tests for statistical difference on the Change from baseline in metabolic markers between each ALT-801 treatment group and Placebo group will be conducted at each timepoint. The p-values from significance tests will be obtained at a two-sided significance level of 0.05 using Dunnett's test for the comparison of change differences.

All metabolic markers parameters will be listed for the extension part of the study.

6.2.5 Inflammatory Markers at 24 Weeks for Combined 105/106

Blood will be collected for the assessment of inflammatory markers at Day 1, Day 85 ALT-801-105 / Day 1 ALT-801-105, and Day 85/ET ALT-801-106 and includes the following parameters:

- Tumor necrosis factor (TNF)
- High-sensitivity C-reactive protein (hs-CRP)
- Monocyte chemoattractant protein-1 (MCP-1)
- Interleukin-6 (IL-6)
- Plasminogen activator inhibitor-1 (PAI-1)

Observed values at baseline, Day 85 and Day 169 (ALT-801-105/ALT-801-106) with Change from baseline will be summarized by treatment group for pooled data with descriptive statistics (sample size [N], arithmetic mean, SD, median, minimum, and maximum).

The **Change from baseline** in inflammatory markers will be compared between each ALT-801 groups and Placebo group using the analysis of covariance (ANCOVA) at each timepoint. The model will be fit with inflammatory markers **Change from baseline** as dependent variable and treatment group and the stratum (diabetic, non-diabetic) as factors, **baseline** value as covariate and the corresponding **baseline** demographic characteristics (gender, race, BMI) as covariates. The least square means and 95% CI of LSM will be reported for variables analyzed with the ANCOVA model. Tests for statistical difference on the Change from baseline in inflammatory markers between each ALT-801 treatment group and Placebo group will be conducted at each timepoint. The p-values from significance tests will be obtained at a two-sided significance level of 0.05 using Dunnett's test for the comparison of change differences.

All inflammatory markers parameters will be listed for the extension part of the study.

6.2.6 Fibrosis Markers at 24 Weeks for Combined 105/106

Blood will be collected for the assessment of fibrosis markers, at Day 1, Day 85 ALT-801-105 / Day 1 ALT-801-106, and Day 85/ET ALT-801-106 following an overnight fast of at least 8 hours and includes the following parameters:

- Enhanced Liver Fibrosis (ELF)
- N-terminal type III collagen propeptide (PRO-C3)
- Liver inflammatory-fibrotic activity by corrected T1 MRI scanning (cT1)
- Fibroscan (steatosis- CAP, liver stiffness - LSM)
- Hyaluronic Acid (ng/mL)(HA)
- Procollagen III Amino-terminal Peptide (ng/mL) (PIIINP)
- Tissue Inhibitor of Metalloproteinase (ng/mL) (TIMP1)

FibroScan, an ultrasound-like instrument able to simultaneously measure liver stiffness and steatosis through Vibration-Controlled Transient Elastography (VCTE) and controlled attenuation parameter (CAP) (kPa.) will be administered during screening, Day 85 ALT-801-105 / Day 1 ALT-801-105, and Day 85/ET ALT-801-106, following an overnight fast of at least 8 hours, to assess changes in liver fat and fibrotic activity. Elasticity measurements will be reported by liver stiffness measurement (LSM) (kPa).

Observed values at baseline, Day 85 and Day 169 (ALT-801-105/ALT-801-106) with Change from baseline in fibrosis markers will be summarized by treatment group for pooled data with descriptive statistics (sample size [N], arithmetic mean, SD, median, minimum, and maximum).

The **Change from baseline** in fibrosis markers will be compared between each ALT-801 groups and Placebo group using the analysis of covariance (ANCOVA) at each timepoint. The model will be fit with fibrosis markers **Change from baseline** as dependent variable and treatment group and the stratum (diabetic, non-diabetic) as factors, **baseline value** as covariate and the corresponding **baseline** demographic characteristics (gender, race, BMI) as covariates. The least square means and 95% CI of LSM will be reported for variables analyzed with the ANCOVA model. Tests for statistical difference on the Change from baseline in fibrosis markers between each ALT-801 treatment group and Placebo group will be conducted at each timepoint. The p-values from significance tests will be obtained at a two-sided significance level of 0.05 using Dunnett's test for the comparison of change differences.

All Fibroscan, ELF, PRO-C3 and liver inflammatory-fibrotic activity by corrected T1 (cT1) MRI scanning will be listed for the extension part of the study.

6.2.6.1 - cT1 Model-Based LSMEAN Estimates Plots

For the Corrected T1 MRI scanning (cT1), Change from baseline **baseline will** be examined with **line plots** from the model-based LSMEAN estimates with 95% confidence intervals presented by treatment groups and by stratum.

The plot will identify the treatment that the subject was in by color lines (ALT-801 doses vs. Placebo).

6.2.7 Lipotoxicity markers

Blood will be collected for the assessment of lipotoxicity markers, at Day 85 ALT-801-105 / Day 1 ALT-801-106, and Day 85/ET ALT-801-106.

Lipotoxicity and specific lipids to be analyzed will be described in a separate analysis plan.

6.2.8 Multiplicity

Multiple comparison adjustment for the p-values and confidence limits for the differences of LSM will be done using Dunnett's test. Formal statistical analysis will be performed as described for each endpoint. All p-values displayed will be nominal in nature.

6.3 Quality of Life

Quality of life will be assessed during Screening, Day 85 ALT-801-105 / Day 1 ALT-801-106, and Day 85/ET ALT-801-106 by the 36-Item Short Form Health Survey (SF-36) v2 and Impact of Weight on Quality of Life-Lite Clinical Trials version (IWQoL-Lite for CT).

Data will be summarized for the Safety Population.

6.3.1 36-Item Short Form Health Survey (SF-36)

The quality of life will be assessed using the 36-Item Short Form Health Survey (SF-36) v2 validated questionnaire (Ware JE, 2005).

The SF-36 is a multi-item scale measuring the functional status and well-being of the subject with respect to the following 8 health domains:

- (PF) Physical functioning (Q3-Q12),
- (RP) Role limitations due to physical health (Q13-Q16),
- (BP) Pain (Q21-Q22),
- (GH) General health (Q1, Q33-Q36),
- (VT) Energy/ fatigue (Q23, Q27, Q29, Q31),
- (SF) Social functioning (Q20, Q32),
- (RF) Role limitations due to emotional problems (Q17-Q19) and
- (MH) Emotional well-being (Q24-Q26, Q28, Q30).

The eight SF-36 domains will be derived using the algorithm of (Ware JE, 2005).

In addition, the SF-36 includes a general health-reporting item (Q2) which asks subjects the amount of change in their health in general over a one-year period. Item 2 is analyzed and reported independently from General Health scale.

The domain scores will be derived according to sponsor-provided scoring instructions. Each item is recorded in the eCRF with an original item value. All Items are recoded, according to the RAND 36-Item Health Survey scoring reference. All questions are scored on a scale from 0 to 100, with 100 representing the highest level of functioning possible. Aggregate scores are compiled as a percentage of the total points possible, using the RAND scoring table (STEP I chart). The scores from those questions that address each specific area of functional health status (STEP II chart) are then averaged together, for a final score within each of the 8 dimensions measured. (eg pain, physical functioning etc.).

All 8 domains are scored in the same way.

Observed values at extension baseline and Day 85 ALT-801-106 with Change from extension baseline will be summarized by treatment group for each stratum (diabetic, non-diabetic) and pooled data with descriptive statistics (sample size [N], arithmetic mean, SD, median, minimum, and maximum).

The Change from extension baseline at Day 85 ALT-801-106 in the eight domains scores and two summary scores will be compared between each ALT-801 groups and Placebo group using the analysis of covariance (ANCOVA) at each timepoint. The model will be fit with each parameter's **Change from extension baseline** as dependent variable and treatment group and the stratum (diabetic, non-diabetic) as factors, **extension baseline value** as covariate and the corresponding **extension baseline demographic** characteristics (gender, race, BMI) as covariates. The least square means and 95% CI of LSM will be reported for variables analyzed with the ANCOVA model. Tests for statistical difference on the Change from extension baseline in reported scores between each ALT-801 treatment group and Placebo group will be conducted at each timepoint. The p-values from significance tests will be obtained at a two-sided significance level of 0.05 using Dunnett's test for the comparison of change differences.

6.3.2 Impact of Weight on Quality of Life-Lite Clinical Trials version (IWQoL-Lite for CT)

The IWQoL-Lite for CT is a 20-item modified version of a questionnaire tool designed to assess the weight-related quality of life. Items are scored with 5-point graded response scales (1 = never, 2 = rarely, 3 = sometimes, 4 =usually, 5 = always; or 1 = not at all true, 2 = a little true, 3 = moderately true, 4 = mostly true, 5= completely true).

Scores are obtained on 3 composite scales (Physical, Physical Function, and Psychosocial), as well as total score.

- Physical (seven items Q1-Q5, Q16-Q17) (including Physical Function)
- Physical Function (five items Q1-Q3, Q16-Q17)
- Psychosocial (13 items Q6-Q15, Q18-Q20)
- IWQOL-Lite-CT Total = Items 1-20

The IWQOL-Lite-CT are reverse scored, and the composites rescaled to range between 0 and 100, such that higher composite scores indicate higher levels of functioning. The composite scores will be derived according to sponsor-provided scoring instructions.

Observed values at extension baseline and Day 85/ET ALT-801-106 with **Change from extension** baseline will be summarized by treatment group for each stratum (diabetic, non-diabetic) and pooled data with descriptive statistics (sample size [N], arithmetic mean, SD, median, minimum, and maximum).

The **Change from extension baseline** in the composite scores and total score will be compared between each ALT-801 groups and Placebo group using the analysis of covariance (ANCOVA) at each timepoint. The model will be fit with each parameter's **Change from extension baseline** as dependent variable and treatment group and the stratum (diabetic, non-diabetic) as factors, **extension baseline value** as covariate and the corresponding **extension baselin**e demographic characteristics (gender, race, BMI) as covariates. The least square means and 95% CI of LSM will be reported for variables analyzed with the ANCOVA model. Tests for statistical difference on the Change from extension baseline in reported scores between each ALT-801 treatment group and Placebo group will be conducted at each timepoint. The p-values from significance tests will be obtained at a two-sided significance level of 0.05 using Dunnett's test for the comparison of change differences.

The IWQoL-Lite is sensitive to the degree of obesity and responsive to weight loss and weight gain. Scores range from 0 to 100 (with lower scores indicating greater impairment).

To explore the relationship between weight change and IWQOL-Lite total score, scatterplots will be provided to display the percent weight change from extension baseline at Day 85 along with the change in IWQoL from extension baseline. A best-fit line of regression may be included in the plot.

Data will be listed for safety population.

6.4 Pharmacokinetics

No PK samples are being collected in the extension study.

6.5 Safety

All safety analysis reporting will be based on the Safety Population, by treatment groups .

6.5.1 Adverse Events

Adverse events (AEs) will be recorded from the time of informed consent through the Follow-up visit (protocol section 9.2).

All ongoing Adverse Events from ALT-801-105 will be entered on Adverse Event Details form of ALT-801-106 study. Site should enter data for Adverse events forms for ALT-801-106 study only.

An integrated analysis of all adverse events reported in studies ALT-801-105 and ALT-801-106 will be presented

Reconciliation of the potential duplicate records between the two databases will not be in the scope of this analysis. A medical occurrence will be reported as a treatment-emergent AE (TEAE) if it 1) was defined as a TEAE within the ALT-801-105 study, 2) is a new occurrence since the first dose of study medication on Day 1 of extension Study ALT-801-106, 3) worsening in the severity of a previously reported AE in this study or Study ALT-801-105, or 4) the development of seriousness criteria in a previously reported AE.

Dates and times for AEs will be imputed as described in Appendix 1. AEs with a missing start date or time that leads to ambiguity about whether the AE is treatment emergent will be considered treatment emergent.

Missing severity grades or missing relatedness assessments will not be imputed. Adverse events will be coded based on the latest version of Medical Dictionary for Regulatory Affairs (MedDRA) for reporting by system organ class (SOC) and preferred term (PT).

The number of individual TEAEs and the incidence of TEAEs will be summarized overall by system organ class (SOC) and PT. For each level of SOC and PT, a subject will be counted only once for the purpose of summarization.

The Overall Summary of Treatment-Emergent Adverse Events and the Summary of TEAEs by System Organ Class and Preferred will be presented by stratum (diabetic / non-diabetic) and pooled data.

The incidence of treatment-emergent SAEs, TEAEs leading to study discontinuation, and TEAEs related to study drug (Possibly, probably) will be summarized by SOC and PT.

Separate summaries will be presented for TEAE resulting in study drug interruption or discontinuation by SOC and PT.

In the summary tables (except the summaries of the TEAEs reported by maximum severity) the number and percentage of subjects reporting at least one TEAE will be presented, For each SOC, the number and percentage of subjects reporting one or more TEAEs within that SOC will be reported. Then, each PT within that SOC will be reported, showing number and percentage of subjects reporting at least one TEAE classified as that particular PT. The summary will be

produced at the SOC level according to the MedDRA coding, and then within the SOC by descending frequency at the PT level.

For the summary of TEAE by maximum severity grade, if a subject has more than one event with different severities within a given PT, then the most severe event will be included in the table. Within each SOC, each TEAE listed by PT will have up to five associated rows, one for each observed severity grade (Grade 1 – Grade 5). The number and percentage of subjects who have at least one TEAE with that PT at that greatest severity will be shown.

A comprehensive listing of all treatment-emergent and non-treatment-emergent AEs will be provided by treatment group, and subject for AEs accrued under current protocol only.

- All AEs
- all TEAE
- Serious AEs (SAEs)
- TEAEs related to study drug
- TEAEs leading to study withdrawal

6.5.2 Injection Site Reactions

Injection site reaction (ISR) will be identified in the eCRF AE page and may include the following:

- Pain
- Tenderness
- Erythema/Redness
- Induration/Swelling
- Other

ISRs will be summarized overall by system organ class (SOC) and PT. For each level of SOC and PT, a subject will be counted only once for the purpose of summarization.

A listing of all injection site reactions will be provided by treatment group, and subject for the extension part of the study.

6.5.3 Clinical Laboratory Evaluations

Laboratory tests will be collected as specified in the SOA in the Protocol Table 1.

Laboratory tests will be reported based on the International System of Units (SI) and are listed in Appendix 2.

Observed values at extension baseline, Day 22, Day 43, Day 64, Day 85 and Day 110 ALT-801-106 and changes from extension baseline for hematology and coagulation, clinical chemistry, and continuous urinalysis will be summarized at each visit by treatment group with descriptive statistics (N, arithmetic mean, SD, CV%, median, minimum, and maximum) for the Safety Population for the extension part of the study.

For the safety labs and urinalysis, the most extreme change from extension baseline will also be summarized. These maximum and minimum extreme changes from extension baseline will be based on the largest extension post-baseline value and the smallest extension post-baseline value, using all available extension post-baseline assessments, including unscheduled assessments for the Safety Population for the extension part of the study.

Laboratory data will also be summarized in shift from extension baseline to each visit and most extreme change from extension baseline based on range categories of low (below lower limit of normal [LLN], normal, and high [above upper limit of normal [ULN]) for the Safety Population.

The summary of observed results and change from extension baseline and shifts from extension baseline to each visit for fasting glucose only will be presented by stratum (diabetic /non-diabetic).

Laboratory tests will be reported for data collected during the extension study ALT-801-106.

All laboratory parameters will be provided in subject data listings for the extension part of the study and values that are outside normal ranges will be flagged for the Safety.

Additionally, results from tests performed at Screening, FSH (post-menopausal women only), serum and urine pregnancy tests, will be presented in listings for Safety Population for the extension part of the study.

Calcitonin data will be summarized separately from safety laboratory tests using descriptive statistics (N, arithmetic mean, SD, median, minimum, and maximum) and will be expressed in ng/L. The summary will be done for the Safety population for the extension part of the study.

The following imputation rule will be applied to the lab values that are too small or too big to detect meaningful signals. These lab values are displayed in a character format with less than (i.e., <) or bigger than (i.e., >) symbols.

- If a lab value is less than a reference number (e.g., <10), which is within a normal test range (e.g., from 0 to 9), then that lab value will be replaced by a half of that reference number (e.g., 5=10/2).
- If a lab value is greater than a reference number (e.g., > 900), which is abnormal or out of normal range (e.g., from 0 to 900), then that lab value will be replaced by the reference number (e.g., 900).

6.5.4 Blood Glucose by Glucometer

The primary analysis of fasting glucose will be based on the safety laboratories.

Fasting glucose (FPG) levels will also be measured by a glucometer and documented by study staff at baseline and prior to each dose as indicated in extension protocol Table 1. On non-visit days, subjects will also monitor and record FPG each morning and will contact the study site for a reading >240mg/dL or <70mg/dL. Subjects will also be educated on symptoms and treatment of hypoglycemia and will obtain a glucometer reading if they experience plasma glucose <70 mg/mL or symptoms suggestive of hypoglycemia, as described in protocol Appendix 3. Subjects will

record any symptoms of hypoglycemia experienced at home in a log, which will be reviewed by the Investigator at each visit.

Observed values and changes from **extension baseline** in FPG by glucometer glucose test will be summarized at each visit (as recorded by the site at Day 8, 15, 22, 29, 36, 43, 50, 57, 64, 71, 78, 85, 110 ALT-801-106) by treatment group with descriptive statistics (N, arithmetic mean, SD, CV%, median, minimum, and maximum) for Safety Population.

Blood glucose by glucometer will be reported for data collected during the extension study ALT-801-106 by stratum (diabetic /non-diabetic) and pooled data.

For the values collected at the site and self-monitored reported values, the most extreme change from extension baseline will also be summarized. These maximum and minimum extreme changes from extension baseline will be based on the largest extension post-baseline value and the smallest extension post-baseline value, using all available extension post-baseline assessments, including unscheduled assessments.

Blood glucose by glucometer will be reported for data collected during the extension study ALT-801-106.

6.5.5 Vital Signs and RPP

Vitals signs are collected at each dosing day (Week1-Week12), Day 85/ET and Day 110 Follow-Up of the extension study ALT-801-106 for the following parameters:

- Respiratory Rate (breaths/min);
- Heart Rate (beats/min);
- Temperature (ºC);
- Systolic and Diastolic Blood Pressure (mmHg);
- Rate Pulse Product (bpm * mmHg) expressed as the product of mean heart rate and mean systolic blood pressure.

Observed values at extension baseline and Day 8, 15, 22, 29, 36, 43, 50, 57, 64, 71, 78, 85, 110 ALT-801-106 and changes from extension baseline for vital signs will be summarized at each visit by treatment group with descriptive statistics (N, arithmetic mean, SD, median, minimum, and maximum) for the Safety population, Vital signs will be reported for data collected during the extension study ALT-801-106.

Additionally, all vital signs results will be presented in subject data listings for the Safety population.

6.5.6 Electrocardiogram (ECG)

ECG is performed at Screening, Day 1, Day 43, Day 85/ET and Day 110 Follow-Up of the extension study ALT-801-106 and the following parameters are reported:

- Heart rate (breaths/min)
- QT interval (ms)

- PR interval (ms)
- QRS interval (ms)
- RR interval (ms)

Observed values at extension baseline, Day 43, Day 85/ET and Day 110/Follow-up ALT-801-106, and **changes from extension baseline** for ECG parameters will be summarized at each visit by treatment group with descriptive statistics (N, arithmetic mean, SD, CV%, median, minimum, and maximum) for the Safety population.

ECG parameters will be reported for data collected during the extension study ALT-801-106.

ECG's clinical significance will be interpreted using the following categories: within normal limits, abnormal without clinical significance, or abnormal with clinical significance. A shift table from extension baseline to each extension post-baseline visit in the ECG's clinical interpretation will be presented for the Safety population.

Additionally, all ECG results will be presented in subject data listings.

6.5.7 Physical Examinations

Physical examinations will be presented in subject data listings.

6.5.8 Immunogenicity

The analysis of immunogenicity will be described in a separate analysis plan.

Listings will be produced for the Safety population extension.

6.6 Timing of Analyses

An interim analysis will be conducted after all subjects complete 12 weeks of treatment (Day 85 visit of this study). The timing of this interim is expected to occur post-lock, following unblinding of the database.

Analyses to be conducted are summarized in Section 6.7.

6.7 Interim Analysis

An interim analysis will be conducted after all subjects complete 12 weeks of treatment. The interim analysis will include the following summary tables produced for the mITT population (and PD population, if deemed necessary by Sponsor) from Combined 105/106 study data (24 weeks of treatment).

- Table 14.2.1.1: MRI- PDFF hepatic fraction and Liver Volume
- Table 14.2.2.1: proportions of subjects with 30%, 40%, 50% relative reduction
- Table 14.2.4.1: anthropometric parameters
- Table 14.2.5.1: body weight including the summary of subjects whose weight loss percent from baseline greater than 5% or 10%
- Table 14.2.6.1 lipids parameters

Line plots will be provided for the following parameters over time for the mITT population (and PD population, if deemed necessary by Sponsor) Combined 105/106 study data:

- Figure 14.2.1.1: Hepatic Fat Fraction and Liver Volume via MRI-PDFF
- Figure 14.2.4.1.1: anthropometric parameters
- Figure 14.2.5.1.1: body weight loss
- Figure 14.2.6.1: lipids parameters

Interim analysis summary tables and figures are provided in shells document and are identified by an asterisk.

7 APPENDICES

7.1 Appendix 1: Partial Date Conventions

Algorithm for Treatment Emergence of Adverse Events (extension study only for AE onset after Day 1 Study ALT-801-106):

START DATE	STOP DATE	ACTION
Known	Known/Partial/ Missing	If start date/time < study drug start date/time, or start date > Follow-Up (week 18) date, then not TEAE<br>If start date/time >= study drug start date/time and start date <= Follow-Up date, then TEAE
Partial, but known components show that it cannot be on or after study drug start date	Known/Partial/ Missing	Not TEAE
Partial, could be on or after study drug start date	Known	If stop date /time< study drug start date/time, then not TEAE<br>If stop date >= study drug start date, then TEAE
	Partial	Impute stop date as latest possible date (i.e., last day of month if day unknown or 31st December if day and month are unknown), then:<br>If stop date < study drug start date, then not TEAE<br>If stop date >= study drug start date, then TEAE
	Missing	Assumed TEAE
Missing	Known	If stop date/time < study drug start date/time, then not TEAE<br>If stop date >= study drug start date, then TEAE
	Partial	Impute stop date as latest possible date (i.e., last day of month if day unknown or 31st December if day and month are unknown), then:<br>If stop date < study drug start date, then not TEAE<br>If stop date >= study drug start date, then TEAE
	Missing	Assumed TEAE

Algorithm for Treatment Emergence of Concomitant Medication (extension study only if reported after Day 1 Study ALT-801-106):

**START DATE**	**STOP DATE**	**ACTION**
Known	Known	If stop date < study drug start date, assign as prior<br>If stop date >= study drug start date and start date <= end of treatment + 30 days, assign as concomitant
	Partial	Impute stop date as latest possible date (i.e., last day of month if day unknown or 31st December if day and month are unknown), then:<br>If stop date < study drug start date, assign as prior<br>If stop date >= study drug start date and start date <= end of treatment + 30 days, assign as concomitant
	Missing	If "Ongoing" is flagged, assign as concomitant<br>If "Ongoing" is not flagged and stop date is missing, assign as concomitant<br>If start date <= end of treatment + 30 days, assign as concomitant
Partial	Known	Impute start date as earliest possible date (i.e., first day of month if day unknown or 1st January if day and month are unknown), then:<br>If stop date < study drug start date, assign as prior<br>If stop date >= study drug start date and start date <= end of treatment + 30 days, assign as concomitant
	Partial	Impute start date as earliest possible date (i.e., first day of month if day unknown or 1st January if day and month are unknown) and impute stop date as latest possible date (i.e., last day of month if day unknown or 31st December if day and month are unknown), then:<br>If stop date < study drug start date, assign as prior<br>If stop date >= study drug start date and start date <= end of treatment + 30 days, assign as concomitant
	Missing	If "Ongoing" is flagged, assign as concomitant<br>Impute start date as earliest possible date (i.e., first day of month if day unknown or 1st January if day and month are unknown), then:<br>If stop date is missing, assign as concomitant<br>If start date <= end of treatment + 30 days, assign as concomitant
Missing	Known	If stop date < study drug start date, assign as prior<br>If stop date >= study drug start date, assign as concomitant
	Partial	Impute stop date as latest possible date (i.e., last day of month if day unknown or 31st December if day and month are unknown), then:<br>If stop date < study drug start date, assign as prior<br>If stop date >= study drug start date, assign as concomitant
	Missing	If "Ongoing" is flagged, assign as concomitant<br>If "Ongoing" is not flagged, assign as concomitant

7.2 Appendix 2: Laboratory Parameters

Chemistry	Hematology (CBC) and Coagulation	Dipstick Urinalysis [a] (UA)
Albumin	Hemoglobin	Color and appearance
Alanine aminotransferase (ALT)	Hematocrit	pH and specific gravity
Alkaline phosphatase (ALP)	Mean corpuscular hemoglobin (MCH)	Bilirubin
Amylase	Mean corpuscular hemoglobin concentration (MCHC)	Blood
Aspartate aminotransferase (AST)	Mean corpuscular volume (MCV)	Glucose
Bicarbonate		Ketones
Calcium	Platelet count	Leukocytes
Chloride	Red blood cell count	Nitrates
Creatinine	White blood cell count	Protein
Creatinine Kinase (CK)	White blood cell differential<br>- Eosinophils (% & absolute)<br>- Basophils (% & absolute)<br>- Neutrophils (% &absolute)<br>- Monocytes (% & absolute)	Urobilinogen
Direct bilirubin (only if total is elevated)	International normalized ratio (INR)	
Estimated glomerular filtration rate (eGFR)		
Gamma glutamyl transferase (GGT)		
Glucose		
Lipase		
Phosphate		
Potassium		
Sodium		
Total bilirubin		
Total protein		
Blood urea nitrogen (BUN)		
